# Discovery and In Vivo Efficacy of Trace Amine-Associated Receptor 1 (TAAR1) Agonist 4-(2-Aminoethyl)-*N*-(3,5-dimethylphenyl)piperidine-1-carboxamide Hydrochloride (AP163) for the Treatment of Psychotic Disorders

**DOI:** 10.3390/ijms231911579

**Published:** 2022-09-30

**Authors:** Mikhail Krasavin, Anatoly A. Peshkov, Alexey Lukin, Kristina Komarova, Lyubov Vinogradova, Daria Smirnova, Evgeny V. Kanov, Savelii R. Kuvarzin, Ramilya Z. Murtazina, Evgeniya V. Efimova, Maxim Gureev, Kirill Onokhin, Konstantin Zakharov, Raul R. Gainetdinov

**Affiliations:** 1 Department of Medicinal Chemistry, Institute of Chemistry, Saint Petersburg State University, Saint Petersburg 199034, Russia; 2 Lomonosov Institute of Fine Chemical Technologies, MIREA—Russian Technological University, Moscow 119454, Russia; 3 Institute of Translational Biomedicine, Saint Petersburg State University, Saint Petersburg 199034, Russia; 4 Center of Bio- and Chemoinformatics, Sechenov First Moscow State Medical University, Moscow 119435, Russia; 5 Accellena Research and Development Inc., 88A Sredniy pr. V.O., Saint Petersburg 199106, Russia

**Keywords:** trace amine-associated receptor 1, agonists, schizophrenia, psychotic disorders, antipsychotic, biogenic amine mimetics, dopamine transporter knockout rats, hyperlocomotion, molecular modeling

## Abstract

Starting from a screening hit, a set of analogs was synthesized based on a 4-(2-aminoethyl)piperidine core not associated previously with trace amine-associated receptor 1 (TAAR1) modulation in the literature. Several structure–activity relationship generalizations have been drawn from the observed data, some of which were corroborated by molecular modeling against the crystal structure of TAAR1. The four most active compounds (EC_50_ for TAAR1 agonistic activity ranging from 0.033 to 0.112 μM) were nominated for evaluation in vivo. The dopamine transporter knockout (DAT-KO) rat model of dopamine-dependent hyperlocomotion was used to evaluate compounds’ efficacy in vivo. Out of four compounds, only one compound (AP163) displayed a statistically significant and dose-dependent reduction in hyperlocomotion in DAT-KO rats. As such, compound AP163 represents a viable lead for further preclinical characterization as a potential novel treatment option for disorders associated with increased dopaminergic function, such as schizophrenia.

## 1. Introduction

Approximately 1% of the human population suffers from schizophrenia, a chronic psychiatric disorder characterized by positive and negative symptoms as well as cognitive deficits [1]. All clinically used antipsychotic drugs used for the treatment of schizophrenia symptoms have a common mechanism of action, which involves the antagonism of dopamine D2 receptors. Older generation or typical antipsychotics more potently block dopamine D2 receptors that cause pronounced side effects including extrapyramidal symptoms (EPS) and hyperprolactinemia. Newer generation or atypical antipsychotic drugs not only block dopamine D2 receptors, but also antagonize serotonin 5-HT2A receptors, which results in a somewhat different side effect profile involving metabolic symptoms such as weight gain and diabetes [2]. Both typical and atypical antipsychotic drugs can effectively reduce psychosis and other positive symptoms of schizophrenia, but they affect negative symptoms much less and essentially do not ameliorate cognitive deficits. Thus, significant effort is being made at the identification of new antipsychotic drugs that would offer therapeutic benefits on all symptoms of schizophrenia without the side effects characteristic of current treatments [3].

Over the last several decades, numerous novel molecular targets have been investigated in the search for antipsychotic drugs for the treatment of schizophrenia symptoms. However, so far, clinical efficacy was shown only in D2 dopamine receptor antagonists. Only recently, Phase 2 clinical study revealed the antipsychotic action of an agonist of novel molecular target for the treatment of schizophrenia—trace amine-associated receptor 1 (TAAR1)—that has no direct antagonistic action on D2 dopamine receptors [4]. TAAR1 is a member of the family of mammalian monoaminergic G protein-coupled receptors (GPCRs) with some members of the family activated by trace amines [5,6]. Endogenous trace amines are decarboxylated derivatives of amino acids that are structurally and functionally close to classical monoamines such as dopamine and serotonin but are present in trace concentrations in the major monoaminergic structures of the brain. In humans, six functional TAAR receptors are known, with TAAR1 being the most investigated member of this receptor family [6]. TAAR1 is expressed in brain areas that are linked to the emergence of schizophrenia symptoms, e.g., the limbic structures, the frontal cortex and dorsal raphe nucleus. Several lines of preclinical studies indicate that activation of TAAR1 can indirectly modulate the activity of dopamine, serotonin and glutamate, whose dysregulations are associated with schizophrenia symptoms. Particularly, it was found that TAAR1 agonists can counteract aberrant behavioral manifestations in various animal models relevant to schizophrenia, including hyperdopaminergic dopamine transporter knockout (DAT-KO) rodents [7].

At present, two independently developed TAAR1 agonists, Ulotaront (SEP-363856, SEP-856, Sunovion Pharmaceuticals Inc.) and Ralmitaront (RG-7906, RO-6889450, F. Hoffmann-La Roche AG), are being tested clinically for the treatment of schizophrenia [6] with the selective partial TAAR1 agonist Ralmitaront undergoing Phase 2 clinical trials and Ulotaront (full TAAR1 agonist with serotonin 5-HT1A agonist activity) being investigated at Phase 3 clinical studies [8,9]. Based on the promising results of Phase 2 clinical studies, where Ulotaront showed significant effects on both positive and negative symptoms of schizophrenia with a safety and tolerability profile comparable to placebo, Ulotaront received a Breakthrough Therapy designation by the US Food and Drug Administration (FDA). Ulotaront lacks a direct antagonistic effect on dopamine D2 receptors [8] and, thus, emerges as the first antipsychotic drug effective clinically in schizophrenia without side effects caused by the blockade of D2 dopamine receptors, such as increased weight, elevated prolactin and glucose or development of EPS [9]. Furthermore, based on the results of preclinical studies it is expected that activation of TAAR1 may exert antianxiety [10], antidepressant [10,11], antiaddictive [12] and anti-compulsive [13] actions, thereby promoting this molecular target as a novel multimodal treatment opportunity for several neuropsychiatric disorders. 

While TAAR1 is emerging as a hot target in psychopharmacology, a relatively small number of potent and selective TAAR1 ligands are known. The majority of preclinical studies with TAAR1 agonists involved either imidazole (e.g., RO5073012) [14] or 2-aminooxazoline (e.g., RO5166017) [15] derivatives. TAAR1 agonists of other chemical classes, such as biguanides derivatives [16] and 2-(2-aminoethyl) triazoles [17], were investigated only through in silico and in vitro activity studies. Thus, in the present study, we attempted to (1) identify novel potent TAAR1 agonists with previously unknown chemical properties, and to (2) demonstrate their effects in vivo in an animal model relevant to schizophrenia. 

**Figure 1:**
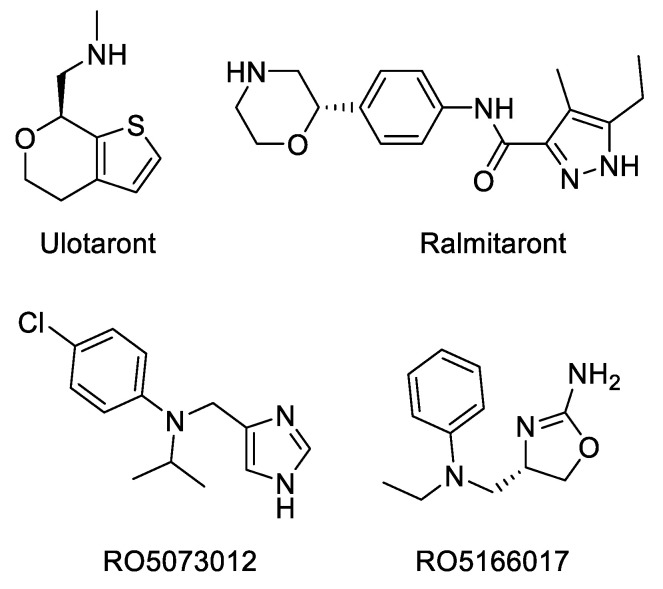
Advanced TAAR1 agonists at various stages of investigation: Ulotaront (phase III clinical trials), Ralmitaront (phase II clinical trials), RO5073012 (preclinical investigation) and RO5166017 (preclinical investigation).

**Figure 2:**
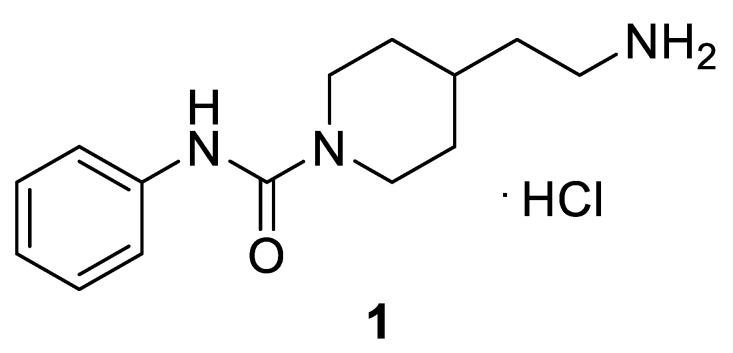
The structure of screening hit **1** selected as a starting point for the current program.

The present study commenced with high-throughput screening. In order to identify new compounds which do not belong to the chemotypes depicted in Figure 1, we performed screening of a focused in-house library of approximately 1000 compounds comprising various heterocyclic motifs in combination with structural fragments similar to β-phenethylamine (PEA) or tyramine, well established ligands of TAAR1. Such a strategy had been reported to deliver higher hit rates in screening a corporate compound collection containing compounds from historic biogenic amine-based drug discovery programs [17]. This hit-finding effort identified a submicromolar agonist of TAAR1 (**1** or 4-(2-aminoethyl)-*N-*phenylpiperidine-1-carboxamide dihydrochloride) which displayed a dose-dependent activation of the receptor and was based on a 4-(2-aminoethyl)piperidine core not associated previously with TAAR1 modulation in the literature (Figure 2). The compound already had a rather promising level of potency (EC_50_ = 0.507 μM) with 65% agonism at TAAR1 relative to 1 μM of tyramine hydrochloride used as a positive control. Moreover, it lacked any stereocenters, which was seen as an advantage as chirality could be introduced later on, at the lead optimization stage, to increase the affinity to the target. Hence, we deemed it as a valid starting point for a hit expansion program aimed at identifying even more potent compounds which could be further evaluated in an animal model of schizophrenia. Herein, we present our discoveries made in the course of fulfilling this goal. 

## 2. Results and Discussion

### 2.1. Compounds Synthesis

Our initial goal was to synthesize a number of aromatic urea analogs in order to improve the starting-point potency of the hit molecule (EC_50_ = 0.507 μM) and, thus, be able to nominate a more potent compound for animal efficacy evaluation. To this end, we sought to synthesize a suitably protected common precursor **2** which would be coupled with various aromatic isocyanates and then deprotected to furnish testable compounds. Likewise, common precursor **2** would be suitable for reacting with other bulky aromatic reagents (such as arenesulfonyl chlorides or benzoyl chlorides) as well as smaller alkyl groups (such as methyl) leading to deeper evaluation of the structure–activity relationships around this novel TAAR1-agonistic chemotype.

**Scheme 1:**
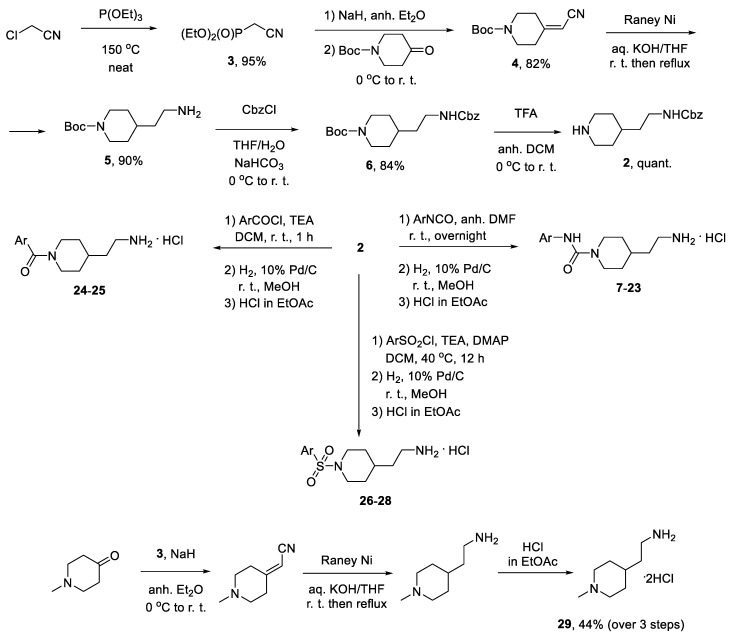
Synthesis of compounds **7**–**29**.

In order to synthesize common core building block **2**, the following synthetic strategy was developed. The Arbuzov reaction between chloroacetonitrile and triethylphosphine furnished cyanide **3** in 95% yield. Deprotonation of the latter with sodium hydride generated an ylide whose reaction with *N*-Boc-piperidone gave nitrile **4** in 82% yield. Reduction of **4** with Raney Ni gave primary amine **5** in 90% yield. Protection of the primary amine with CbzCl gave compound **6** in 84% yield. Finally, treatment of the latter compound with TFA gave the desired core building block **2** in 92% yield. This building block was elaborated in three different ways. Firstly, reaction with aromatic isocyanates followed by reductive removal of the Cbz and generation of a hydrochloride salt furnished ureas **7**–**23** (as well as re-synthesized hit molecule **1**). *N*-Benzoylation gave compounds **24**–**25** and treatment of **2** with arenesulfonyl chlorides furnished compounds **26**–**28**. As a reference molecule, not bearing any bulky aromatic group at the piperidine nitrogen atom, 2-(1-methylpiperidin-4-yl)ethan-1-amine dihydrochloride (**29**) was synthesized from *N*-methylpiperidin-4-one in three steps and overall yield of 44% (Scheme 1).

### 2.2. Agonistic Activity against TAAR1 In Vitro

Compounds **7**–**29** synthesized as described above (as well the initial hit compound **1** used as a comparator) were tested in the Bioluminescence Resonance Energy Transfer approach (BRET) assay [18] using HEK-293 cells transiently transfected with cDNA for TAARs as well as cAMP BRET biosensor (EPAC) protein. The compounds were initially tested at 1 μM or 10 μM concentrations and those producing <20% activation of TAAR1 (relative to 1 μM tyramine hydrochloride) were not pursued further. The active compounds were further evaluated in a dose–response mode in order to determine their EC_50_ values. These data are presented in Table 1. 

As it follows from the data summarized in Table 1, the agonistic activity of the urea series (**1**, **7**–**23**) is quite sensitive to the substitution pattern in the aromatic portion. Even a single methyl substitution (*cf.* **1** vs. **7**, **9** or **10**) could render compounds inactive (**7** and **10**) or improve the activity ten-fold (**9**). Monofluorination also had a tractable effect on the agonistic potency with *m*-fluoro (**8**) and *p*-fluoro (**11**) compounds having no activity and submicromolar potency, respectively. Likewise, various bis-fluorinated aromatic versions varied greatly in activity—from active 2,5-difluoro (**12**) to inactive 2,4-difluoro (**13**) and 3,5-difluoro (**14**). Curiously, the mono- and dimethyl substituted subset (compounds **9**, **16**–**19**) delivered the most potent compounds in the urea series. Of this set, four compounds (**9**, **17**–**19**) were scaled up for in vivo efficacy tests as described below. Another similarly active candidate molecule (**21**) was not progressed due to poor solubility. Compounds **22** and **23** present two interesting structure–activity relationship points, as compound **22** demonstrates two orders of magnitude drop in potency on introduction of just one oxygen atom (*cf.* compound **19**). Inactive compound **23** similarly contrasts to its similarly substituted yet active counterparts, compounds **9** and **21** (and yet is similarly inactive as its direct fluoro analog **8**).

In terms of substitutes for ureas, benzamides **24** and **25** did not show much promise. While simple unsubstituted compound **24** was similar in potency to screening hit **1**, *m*-methyl substitution did not do nearly as good a job at improving the potency of **24** as did, for instance, similar *m*-methyl substitution in the urea series (*cf.* compound **9**). Similarly, none of the sulfonamides **26**–**28** proved workable surrogates for the lead urea series. The inactivity of compound **29** was expected in a way considering the mandatory mimicry of biogenic amines such as tyramine by the bulky aryl(carbamoyl) groups in the active series.

### 2.3. Molecular Modeling

In reviewing the structure–activity relationships summarized in Table 1, we became particularly puzzled by the following two isomeric triads: **7** (inactive)/**9** (0.052 μM)/**10** (inactive) and **12** (0.273 μM)/**13** (inactive)/**14** (inactive). In order to rationalize the structure–activity behavior within these three triads, we undertook docking of these compounds into a model of TAAR1 receptor (Uniprot database Q96RJ0) as the receptor’s crystal structure was not available in the Protein Data Bank (see Experimental Procedures).

**Figure 3:**
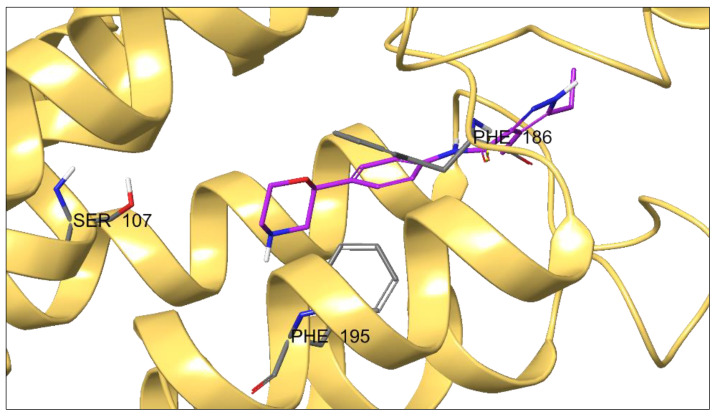
Ralmitaront bound to TAAR1 receptor model.

Unfortunately, when using the molecular docking alone, the resulting scoring functions did not allow us to reliably differentiate between active and inactive compounds in these triads. We found an alternative solution in calculating the Gibbs free energy of binding using the Molecular Mechanics with Generalized Born and Surface Area solvation (MM-GBSA) method [19]. To our delight, in all cases, the active compound within each triad displayed the lowest ΔG value in series, as calculated using this method. Moreover, visual inspection of the ligand binding poses in the active cavity of TAAR1 receptor and comparison of this binding with that of the reference ligand Ralmitaront (Figure 3) revealed that, more importantly, the inactive compounds could not correctly reproduce Ralmitaront’s binding mode, vide infra (Table 2).

**Figure 4:**
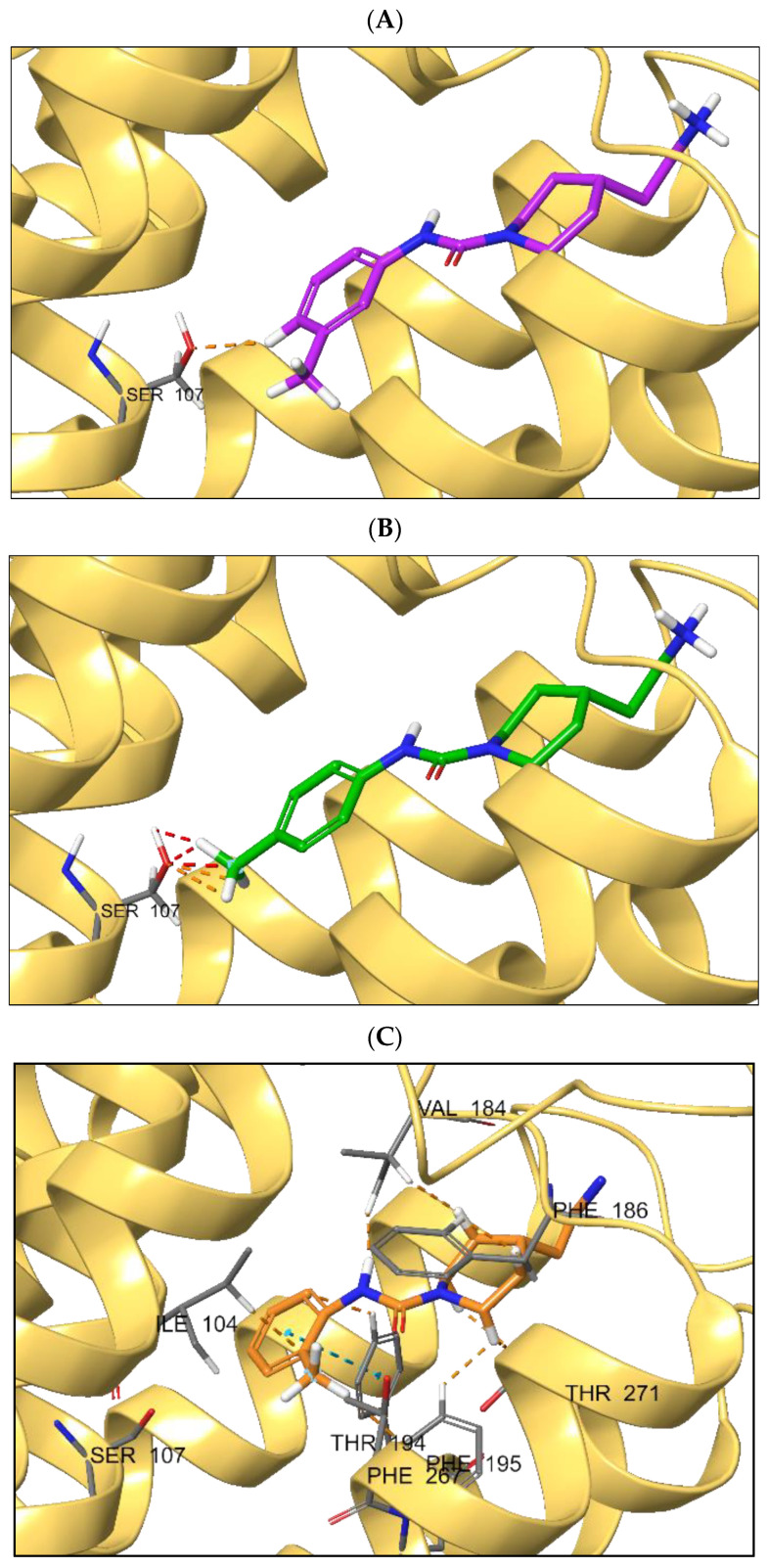
(**A**) Unconstrained docking pose of compound **9**; (**B**) forcibly docked compound **7** with the clash with Ser107 residue; (**C**) forcibly docked compound **10** with a network of unfavorable clashes. Dashed lines of orange and red color—strained protein–ligand contacts (clashes).

Close examination of the ligand binding modes revealed that within the triad **7**/**9**/**10**, only compound **9** could effectively interact with TAAR1 (Figure 4A). If we forcibly dock compounds **7** and **10** in the same manner as **9** was binding, obvious clashes with Ser107 residue (for **7**) or an entire network of clashes with Thr194, Phe186/195/267, Ile104, Thr194/271 residues (for **10**) became evident (Figure 4B,C). Overall, ligand–target contacts of this kind are energetically unfavorable, which is the likely cause of the observed loss of TAAR1 affinity.

**Figure 5:**
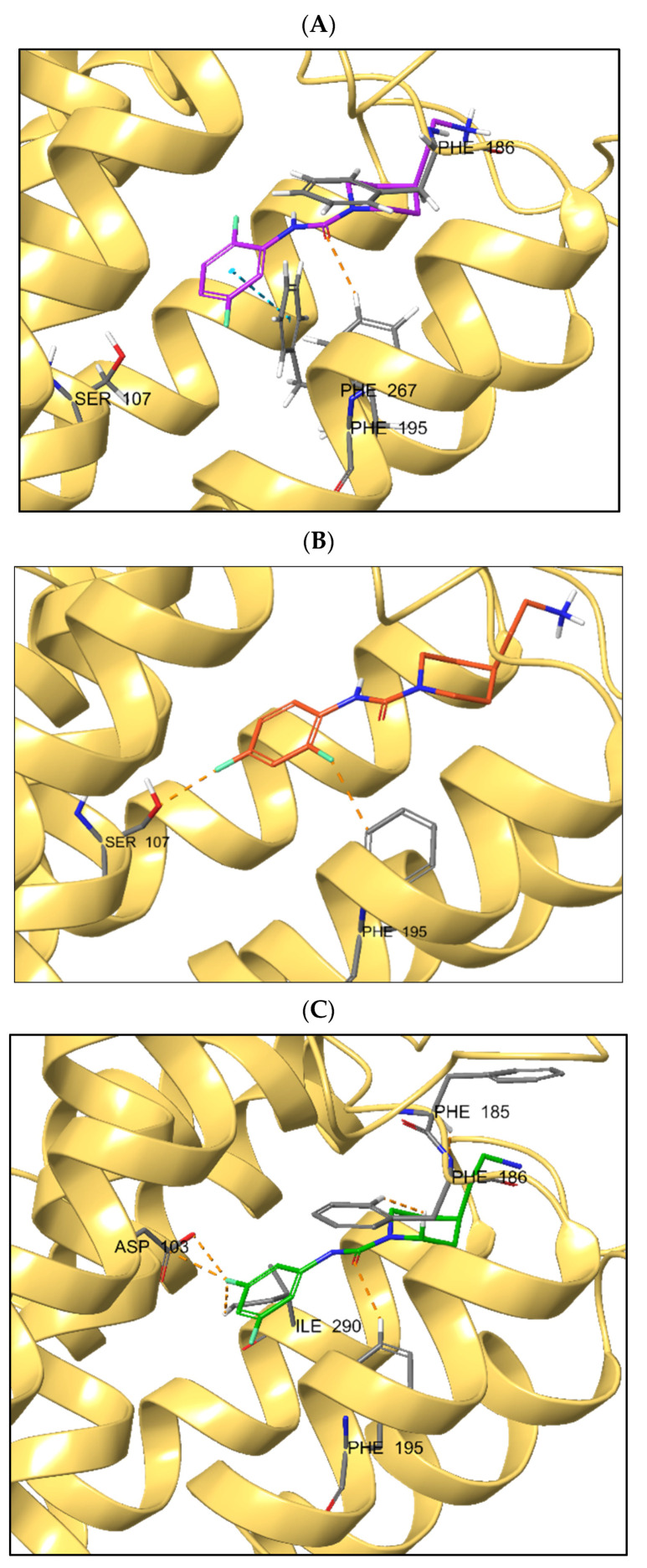
(**A**) Unconstrained docking pose of compound **12**; (**B**) forcibly docked compound **13** with the clash with Ser107 residue; (**C**) forcibly docked compound **14** with a network of unfavorable clashes. Dashed lines of orange and red color—strained protein–ligand contacts (clashes).

A very similar situation was observed for the triad **12**/**13**/**14**. Out of these compounds docked without constraints, only compound **12** displayed effective affinity to the target with a minimum number of strained contacts (Figure 5A). In unconstrained mode, compounds **13** and **14** bound in an inverse mode, or closer to the edge of the active cavity of TAAR1 (not shown). Forced docking of compounds **13** and **14** in the same binding mode as that displayed by compound **12**, again, generated a number of unfavorable interactions with residues Ser107 and Phe195 (compound **13**) and Asp103, Ile290, Phe195/185/186 (compound **14**) which, as in the previous case, resulted in the overall energetic unfavourability of the ligand–protein complex and is the likely reason for the observed absence of activity towards TAAR1. (Figure 5B,C).

### 2.4. In Vivo Efficacy

**Figure 6:**
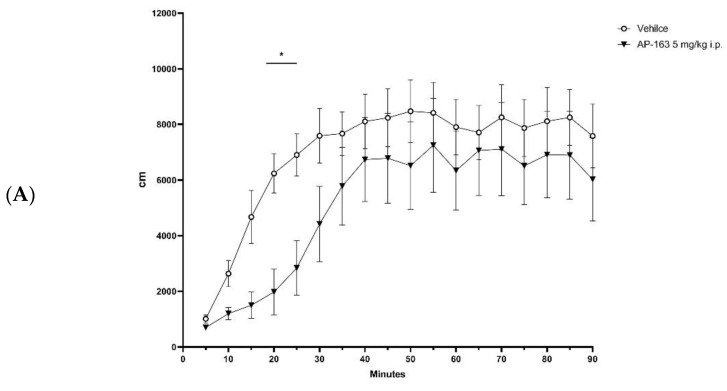

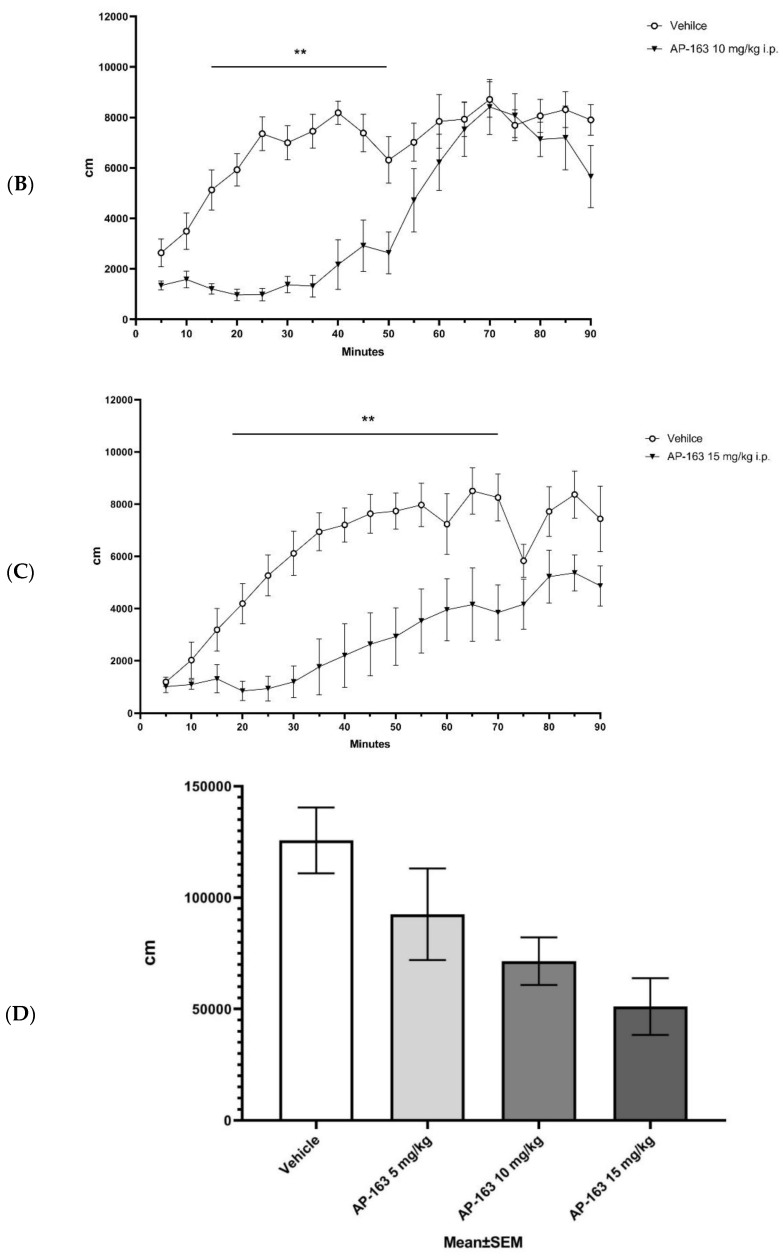
Effect of compound **18** (AP163) on the dynamics of hyperlocomotion of DAT-KO rats at doses (**A**) 5 mg/kg, (**B**) 10 mg/kg and (**C**) 15 mg/kg, and (**D**) the cumulative graph comparing the locomotion (total distance traveled for 90 min after i.p. administration) of the three treatment groups relative to vehicle-treated control group. * *p* < 0.05; ** *p* < 0.01.

Compounds **9**, **17**–**19** were evaluated in dopamine transporter knockout (DAT-KO) rats characterized with psychosis-like hyperlocomotion activity [20]. The ability of compounds to reduce the hyperlocomotion in these genetically modified animals generally deemed as an adequate model of psychosis-like states and schizophrenia [21] was expected to unveil the potential of these compounds as potential therapies for these psychiatric disorders. Previously, several TAAR1 agonists showed potent effect in inhibiting dopamine-dependent hyperactivity in DAT-KO animals, thereby validating the utility of this genetic model in the assessment of in vivo effects of TAAR1 agonists [7,10,20,21]. With all compounds dosed intraperitoneally (i.p.), only one compound, **18** (AP163) showed a statistically significant, dose-dependent reduction in rats’ hyperlocomotion (Figure 6), while other three compounds showed no effect at doses 10 mg/kg (two-way ANOVA *p* < 0.05).

Compound **18** (AP163) in dose range 5–15 mg/kg significantly attenuated DAT-KO hyperlocomotion and the effect was dose-dependent (Figure 6D, Kruskal–Wallis test *p* = 0.0417). At the dose of 5 mg/kg (Figure 6A), assessment of the dynamic of effect for 90 min after administration by two-way ANOVA did not reveal significant effect (*p* > 0.05) but from 15 to 25 min compound **18** (AP163) was effective in decreasing DAT-KO hyperlocomotion (uncorrected Fisher’s LSD test, *p* = 0.0105 0.0147, respectively). At 10 mg/kg (Figure 6B), compound **18** (AP163) significantly decreased hyperlocomotion (two-way ANOVA *p* = 0.0055) and the effect was most pronounced from 15 to 50 min (Bonferroni’s multiple comparisons test, *p* varying from <0.0001 to 0.0188). At 15 mg/kg (Figure 6C), compound **18** (AP163) significantly decreased DAT-KO hyperlocomotion (two-way ANOVA *p* = 0.0051) and the effect was most pronounced from 20 to 70 min (Bonferroni’s multiple comparisons test, *p* varying from 0.0009 to 0.0113) (Figure 6).

Compound **18** was subjected to in silico analysis of its druglikeness profile and prediction of its ADMET properties using www.swissadme.ch online resource (accessed on 14 September 2022). The data are collated in Supplementary Information to this article. According to the analysis, compound **18** is distinctly druglike, with no violations of the Lipinski rules for druglikeness. The compound is predicted to have a high probability of being orally bioavailable and to absorb well in the GI tract. This prompts us to continue evaluating the pharmacological properties of this compound further, as an orally dosed drug. More importantly, compound **18** is predicted to be highly blood–brain barrier permeant, which is commensurate with the observed efficacy of this compound in the in vivo evaluation.

The absence of in vivo efficacy of the other three potent compounds (**9**, **17** and **19**) may have to do with their suboptimal characteristics such as blood–brain barrier penetration and overall poorer pharmacokinetic profile and/or ADMET properties.

## 3. Experimental Procedures

### 3.1. Compound Synthesis-General

NMR spectroscopic data were recorded with 300, 400 and 500 spectrometers (300.13, 400.13 MHz for ^1^H and 75.47, 100.62, 125.72 MHz for ^13^C) in DMSO-*d*_6_ and were referenced to residual solvent proton signals δ_H_ = 2.50 ppm and solvent carbon signals δ_C_ = 39.52. Mass spectra were recorded with a Bruker Maxis HRMS-ESI-qTOF spectrometer (electrospray ionization mode). Column chromatography was carried out with silica gel grade 60 (0.040–0.063 mm) 230–400 mesh. TLC was performed with Macherey-Nagel «Alugram Sil G/UV254» plates. All commercial reagents and solvents were used without further purification. Not available isocyanates were synthesized according to the known procedures.

#### 3.1.1. Synthesis of Diethyl (Cyanomethyl)phosphonate (**3**)

Triethylphosphite (19.9 g, 120 mmol) was heated to 150 °C in a 100 mL 3-neck flask, and chloroacetonitrile (10.9 g, 144 mmol) was added dropwise over a period of 2 h. The mixture was stirred for 2 more h at 150 °C until no chloroethane evolvement. The mixture was distilled under reduced pressure (8 mbar, 132–134 °C). Yield 20.2 g (95%), colorless liquid.

#### 3.1.2. Synthesis of *tert*-Butyl 4-(Cyanomethylene)piperidine-1-carboxylate (**4**)

A solution of diethyl (cyanomethyl)phosphonate (20.2 g, 114 mmol) in anhydrous Et_2_O (30 mL) at 0 °C was added to a stirred suspension of NaH (5.0 g, 125 mmol; 60% dispersion in mineral oil) in anhydrous Et_2_O (180 mL); the resulting mixture was stirred for 15 min. Then, tert-butyl 4-oxopiperidine-1-carboxylate (22.7 g, 114 mmol) was added carefully in small portions. The reaction mixture was allowed to warm up to RT and stirred overnight. The reaction mixture was cooled down to 0 °C and quenched with cold H_2_O (300 mL). Phases were separated and aqueous phase was extracted with CH_2_Cl_2_ (3 × 100 mL). The organic layers were combined, dried over Na_2_SO_4_, filtered and concentrated in vacuo. The residue was purified by column chromatography (eluent: hexanes + EtOAc, gradient: 10–50% of EtOAc). Yield 20.7 g (82%), white solid.

#### 3.1.3. Synthesis of *tert*-Butyl 4-(2-Aminoethyl)piperidine-1-carboxylate (**5**)

A solution of KOH (10.2 g, 182 mmol) in H_2_O (100 mL) was added to a stirred solution of *tert*-butyl 4-(cyanomethylene)piperidine-1-carboxylate (15.6 g, 70 mmol) in THF (100 mL). Then, Ni/Al-alloy (45.5 g, Ni:Al = 50:50) was added in small portions within 4 h while maintaining moderate gas evolution. The reaction mixture was refluxed for 3 h. The precipitate was filtered off and washed with THF. The filtrate was concentrated by evaporating THF in vacuo and the resulting aqueous solution was extracted with CH_2_Cl_2_ (100 mL). The organic phase was dried over Na_2_SO_4_, filtered and concentrated in vacuo to give crude product which was used for next step without further purification. Yield 14.4 g (90%), gray solid.

#### 3.1.4. Synthesis of *tert*-Butyl 4-(2-(((Benzyloxy)carbonyl)amino)ethyl)piperidine-1-carboxylate (**6**)

The solution of *tert*-butyl 4-(2-aminoethyl)piperidine-1-carboxylate (13.0 g, 57 mmol) in THF (110 mL) was mixed with solution of NaHCO_3_ (5.7 g, 68 mmol) in H_2_O (350 mL). The resulting mixture was cooled down to 0 °C and solution of benzyl chloroformate (10.2 g, 60 mmol) in THF (220 mL) was added carefully. The reaction mixture was warmed up to RT and stirred for 15 min. THF was removed in vacuo and the resulting aqueous solution was extracted with EtOAc (350 mL). The organic phase was dried over Na_2_SO_4_, filtered and concentrated in vacuo to give crude product which was purified by column chromatography (eluent: hexanes + EtOAc, gradient: 10–50% of EtOAc). Yield 16.7 g (81%), white solid.

#### 3.1.5. Synthesis of Benzyl (2-(Piperidin-4-yl)ethyl)carbamate (**2**)

TFA (570 mg, 5 mmol) was added carefully to an ice-cold solution of *tert*-butyl 4-(2-(((benzyloxy)carbonyl)amino)ethyl)piperidine-1-carboxylate (181 mg, 0.5 mmol) in CH_2_Cl_2_ (8 mL). The reaction mixture was allowed to stir for 3 h at RT. The solution of NaOH (240 mg, 6 mmol) in H_2_O (10 mL) was added to the reaction mixture at 0 °C. The organic phase was separated, dried over Na_2_SO_4_, filtered and concentrated in vacuo to give crude product which was used immediately for the next step without any purification. (Mixture A)

#### 3.1.6. Synthesis of Benzyl (2-(1-(Arylcarbamoyl)piperidin-4-yl)ethyl)carbamates **1**, **7**–**23**

Corresponding isocyanate was added to the solution of benzyl (2-(piperidin-4-yl)ethyl)carbamate 2 (Mixture A, approximately 0.5 mmol) in anh. DMF (2 mL); the reaction mixture was stirred overnight at RT. The reaction mixture was concentrated in vacuo and the residue was dissolved in CH_2_Cl_2_ (25 mL). The resulting solution was washed with H_2_O (3 × 25 mL) and organic phase was separated, dried over Na_2_SO_4_, filtered and concentrated in vacuo to give crude product which was purified by column chromatography.

The Cbz-protected compound was dissolved in MeOH (15 mL). The solution was purged with argon followed by addition of 10% Pd/C (3 mol % of Pd). The resulting suspension was flashed with argon and hydrogen, and left stirred under the atmosphere of hydrogen gas (1 atm) for 24 h at RT. The reaction mixture was filtered through a pad of celite to remove catalyst and concentrated to provide crude product which was purified by acid/base extraction. The product was dissolved in a small amount of EtOAc and mixed with required amount of HCl solution in EtOAc to give stable at ambient conditions hydrochloride salt. HCl solution in EtOAc was obtained by careful and slow addition of acetyl chloride (100 mL, 110.4 g, 1.41 mol) via dropping funnel to the stirred ice cold EtOH (100 mL, 78.9 g, 1.71 mol).

*(1) 4-(2-Aminoethyl)-N-phenylpiperidine-1-carboxamide hydrochloride* (**1**). Yield 96 mg (68%). ^1^H NMR (300 MHz, DMSO-*d_6_*) δ 8.49 (s, 1H), 7.96 (br.s, 3H), 7.50–7.41 (m, 2H), 7.25–7.15 (m, 2H), 6.96–6.85 (m, 1H), 4.18–4.06 (m, 2H), 2.87–2.66 (m, 4H), 1.73–1.61 (m, 2H), 1.61–1.44 (m, 3H), 1.14–0.96 (m, 2H). ^13^C NMR (75 MHz, DMSO-*d_6_*) δ 154.9, 140.8, 128.2, 121.5, 119.6, 43.9, 36.4, 33.5, 32.6, 31.5. HRMS (ESI) calcd for C_14_H_22_N_3_O^+^ [M+H^+^] 248.1757; found 248.1757.

*(2) 4-(2-Aminoethyl)-N-(4-methylphenyl)piperidine-1-carboxamide hydrochloride (***7***)*. Yield 87 mg (59%). ^1^H NMR (300 MHz, DMSO-*d*_6_) δ 8.39 (s, 1H), 7.98 (br.s, 3H), 7.33 (d, *J* = 8.4 Hz, 2H), 7.01 (d, *J* = 8.3 Hz, 2H), 4.18–4.03 (m, 2H), 2.87–2.65 (m, 4H), 2.21 (s, 3H), 1.72–1.60 (m, 2H), 1.60–1.44 (m, 3H), 1.13–0.94 (m, 2H). ^13^C NMR (75 MHz, DMSO-*d*_6_) δ 155.0, 138.1, 130.3, 128.6, 119.8, 43.8, 36.4, 33.5, 32.6, 31.5, 20.3. HRMS (ESI) calcd for C_15_H_24_N_3_O^+^ [M+H^+^] 262.1914; found 262.1912.

*(3) 4-(2-Aminoethyl)-N-(3-fluorophenyl)piperidine-1-carboxamide hydrochloride (***8***)*. Yield 123 g (82%). ^1^H NMR (300 MHz, DMSO-*d*_6_) δ 8.73 (s, 1H), 7.91 (br.s, 3H), 7.50–7.39 (m, 1H), 7.30–7.16 (m, 2H), 6.77–6.65 (m, 1H), 4.19–4.05 (m, 2H), 2.88–2.68 (m, 4H), 1.74–1.62 (m, 2H), 1.61–1.45 (m, 3H), 1.14–0.97 (m, 2H). ^13^C NMR (75 MHz, DMSO-*d*_6_) δ 162.1 (d, *J* = 239.6 Hz), 154.5, 142.8 (d, *J* = 11.4 Hz), 129.6 (d, *J* = 9.7 Hz), 115.0 (d, *J* = 2.4 Hz), 107.7 (d, *J* = 21.2 Hz), 105.9 (d, *J* = 26.3 Hz), 43.9, 36.4, 33.5, 32.6, 31.5. HRMS (ESI) calcd for C_14_H_21_FN_3_O^+^ [M+H^+^] 266.1663; found 266.1662.

*(4) 4-(2-Aminoethyl)-N-(3-methylphenyl)piperidine-1-carboxamide hydrochloride (***9***)*. Yield 107 mg (72%). ^1^H NMR (300 MHz, DMSO-*d*_6_) δ 8.41 (s, 1H), 7.96 (br.s, 3H), 7.32–7.20 (m, 2H), 7.13–7.03 (m, 1H), 6.77–6.68 (m, 1H), 4.18–4.04 (m, 2H), 2.87–2.65 (m, 4H), 2.23 (s, 3H), 1.72–1.61 (m, 2H), 1.59–1.44 (m, 3H), 1.12–0.95 (m, 2H). ^13^C NMR (75 MHz, DMSO-*d*_6_) δ 154.9, 140.7, 137.2, 128.0, 122.2, 120.2, 116.8, 43.9, 36.4, 33.5, 32.6, 31.5, 21.2. HRMS (ESI) calcd for C_15_H_24_N_3_O^+^ [M+H^+^] 262.1914; found 262.1912.

*(5) 4-(2-Aminoethyl)-N-(2-methylphenyl)piperidine-1-carboxamide hydrochloride (***10***)*. Yield 98 mg (66%). ^1^H NMR (300 MHz, DMSO-*d*_6_) δ 8.15–7.82 (m, 4H), 7.20–6.98 (m, 4H), 4.15–4.01 (m, 2H), 2.87–2.68 (m, 4H), 2.14 (s, 3H), 1.72–1.61 (m, 2H), 1.61–1.46 (m, 3H), 1.15–0.97 (m, 2H). ^13^C NMR (75 MHz, DMSO-*d*_6_) δ 155.5, 138.2, 133.1, 130.0, 126.0, 125.7, 124.4, 44.1, 36.4, 33.6, 32.7, 31.5, 18.0. HRMS (ESI) calcd for C_15_H_24_N_3_O^+^ [M+H^+^] 262.1914; found 262.1913.

*(6) 4-(2-Aminoethyl)-N-(4-fluorophenyl)piperidine-1-carboxamide hydrochloride (***11***)*. Yield 120 mg (79%). ^1^H NMR (300 MHz, DMSO-*d*_6_) δ 8.58 (s, 1H), 8.03 (br.s, 3H), 7.53–7.41 (m, 2H), 7.10–6.98 (m, 2H), 4.19–4.04 (m, 2H), 2.87–2.66 (m, 4H), 1.72–1.61 (m, 2H), 1.60–1.45 (m, 3H), 1.13–0.94 (m, 2H). ^13^C NMR (75 MHz, DMSO-*d*_6_) δ 157.3 (d, *J* = 237.7 Hz), 155.0, 137.1 (d, *J* = 2.4 Hz), 121.4 (d, *J* = 7.6 Hz), 114.6 (d, *J* = 21.9 Hz), 43.9, 36.4, 33.5, 32.6, 31.5. HRMS (ESI) calcd for C_14_H_21_FN_3_O^+^ [M+H^+^] 266.1663; found 266.1663.

*(7) 4-(2-Aminoethyl)-N-(2,5-difluorophenyl)piperidine-1-carboxamide hydrochloride (***12***)*. Yield 110 mg (69%). ^1^H NMR (300 MHz, DMSO-*d*_6_) δ 8.42 (s, 1H), 8.02 (br.s, 3H), 7.44–7.34 (m, 1H), 7.27–7.15 (m, 1H), 6.94–6.83 (m, 1H), 4.14–4.01 (m, 2H), 2.87–2.70 (m, 4H), 1.73–1.62 (m, 2H), 1.60–1.45 (m, 3H), 1.14–0.97 (m, 2H). ^13^C NMR (75 MHz, DMSO-*d*_6_) δ 157.6 (dd, *J* = 238.0, 1.9 Hz), 154.5, 150.9 (dd, *J* = 240.4, 2.5 Hz), 129.4 (dd, *J* = 13.5, 11.8 Hz), 116.1 (dd, *J* = 22.8, 10.0 Hz), 111.4 (dd, *J* = 27.2, 2.2 Hz), 110.0 (dd, *J* = 24.1, 7.8 Hz), 44.1, 36.4, 33.4, 32.5, 31.4. HRMS (ESI) calcd for C_14_H_20_F_2_N_3_O^+^ [M+H^+^] 284.1569; found 284.1573.

*(8) 4-(2-Aminoethyl)-N-(2,4-difluorophenyl)piperidine-1-carboxamide hydrochloride (***13***)*. Yield 108 mg (68%). ^1^H NMR (300 MHz, DMSO-*d*_6_) δ 8.29 (s, 1H), 7.99 (br.s, 3H), 7.42–7.30 (m, 1H), 7.27–7.15 (m, 1H), 7.05–6.94 (m, 1H), 4.13–4.00 (m, 2H), 2.87–2.68 (m, 4H), 1.72–1.61 (m, 2H), 1.61–1.45 (m, 3H), 1.14–0.96 (m, 2H). ^13^C NMR (75 MHz, DMSO-*d*_6_) δ 158.6 (dd, *J* = 242.6, 11.5 Hz), 155.8 (dd, *J* = 248.2, 12.6 Hz), 155.1, 127.8 (dd, *J* = 9.5, 3.1 Hz), 124.4 (dd, *J* = 12.0, 3.6 Hz), 110.6 (dd, *J* = 21.8, 3.6 Hz), 103.9 (dd, *J* = 26.5, 24.6 Hz), 44.0, 36.4, 33.5, 32.6, 31.4. HRMS (ESI) calcd for C_14_H_20_F_2_N_3_O^+^ [M+H^+^] 284.1569; found 284.1530.

*(9) 4-(2-Aminoethyl)-N-(3,5-difluorophenyl)piperidine-1-carboxamide hydrochloride (***14***)*. Yield 114 mg (71%). ^1^H NMR (300 MHz, DMSO-*d*_6_) δ 9.07–8.94 (m, 1H), 7.99 (br.s, 3H), 7.38–7.21 (m, 2H), 6.77–6.61 (m, 1H), 4.20–4.07 (m, 2H), 2.89–2.68 (m, 4H), 1.74–1.62 (m, 2H), 1.62–1.43 (m, 3H), 1.13–0.95 (m, 2H). ^13^C NMR (75 MHz, DMSO-*d*_6_) δ 162.3 (dd, *J* = 241.1, 15.7 Hz), 154.3, 143.9 (t, *J* = 14.2 Hz), 101.9–101.6 (m), 96.1 (t, *J* = 26.2 Hz), 44.0, 36.5, 33.5, 32.6, 31.5. HRMS (ESI) calcd for C_14_H_20_F_2_N_3_O^+^ [M+H^+^] 284.1569; found 284.1568.

*(10) 4-(2-Aminoethyl)-N-(3-fluoro-4-methylphenyl)piperidine-1-carboxamide hydrochloride (***15***)*. Yield 107 mg (68%). ^1^H NMR (300 MHz, DMSO-*d*_6_) δ 8.63 (s, 1H), 8.00 (br.s, 3H), 7.44–7.34 (m, 1H), 7.20–7.03 (m, 2H), 4.18–4.05 (m, 2H), 2.87–2.66 (m, 4H), 2.13 (s, 3H), 1.73–1.61 (m, 2H), 1.61–1.45 (m, 3H), 1.13–0.95 (m, 2H). ^13^C NMR (75 MHz, DMSO-*d*_6_) δ 160.2 (d, *J* = 239.2 Hz), 154.7, 140.4 (d, *J* = 11.2 Hz), 130.7 (d, *J* = 6.5 Hz), 116.2 (d, *J* = 17.4 Hz), 115.0 (d, *J* = 2.8 Hz), 106.0 (d, *J* = 27.0 Hz), 43.9, 36.4, 33.5, 32.6, 31.5, 13.5 (d, *J* = 2.9 Hz). HRMS (ESI) calcd for C_15_H_23_FN_3_O^+^ [M+H^+^] 280.1820; found 280.1817.

*(11) 4-(2-Aminoethyl)-N-(4-fluoro-3-methylphenyl)piperidine-1-carboxamide hydrochloride (***16***)*. Yield 90 mg (57%). ^1^H NMR (400 MHz, DMSO-*d*_6_) δ 8.43 (s, 1H), 7.89 (br.s, 3H), 7.40–7.31 (m, 1H), 7.31–7.20 (m, 1H), 7.03–6.91 (m, 1H), 4.18–4.03 (m, 2H), 2.90–2.63 (m, 4H), 2.22–2.11 (m, 3H), 1.73–1.62 (m, 2H), 1.60–1.44 (m, 3H), 1.12–0.98 (m, 2H). ^13^C NMR (101 MHz, DMSO-*d*_6_) δ 155.8 (d, J = 237.0 Hz), 154.9, 136.7 (d, J = 2.5 Hz).123.3 (d, J = 17.8 Hz), 122.7 (d, J = 4.3 Hz), 118.7 (d, J = 7.6 Hz), 114.2 (d, J = 22.8 Hz), 43.9, 36.8, 34.6, 32.7, 31.5, 14.3 (d, J = 3.0 Hz). HRMS (ESI) calcd for C_15_H_23_FN_3_O^+^ [M+H^+^] 280.1820; found 280.1820.

*(12) 4-(2-Aminoethyl)-N-(3,4-dimethylphenyl)piperidine-1-carboxamide hydrochloride (***17***)*. Yield 92 mg (59%). ^1^H NMR (400 MHz, DMSO-*d*_6_) δ 8.29 (s, 1H), 7.96 (br.s, 3H), 7.28–7.19 (m, 1H), 7.19–7.12 (m, 1H), 6.98–6.92 (m, 1H), 4.15–4.05 (m, 2H), 2.87–2.76 (m, 2H), 2.76–2.66 (m, 2H), 2.18–2.09 (m, 6H), 1.72–1.60 (m, 2H), 1.60–1.45 (m, 3H), 1.12–0.96 (m, 2H). ^13^C NMR (101 MHz, DMSO-*d*_6_) δ 155.0, 138.3, 135.5, 129.1, 129.0, 121.1, 117.3, 43.8, 36.4, 33.5, 32.6, 31.5, 19.6, 18.6. HRMS (ESI) calcd for C_16_H_26_N_3_O^+^ [M+H^+^] 276.2070; found 276.2072.

*(13) 4-(2-Aminoethyl)-N-(3,5-dimethylphenyl)piperidine-1-carboxamide hydrochloride (***18***)*. Yield 95 mg (61%). ^1^H NMR (400 MHz, DMSO-*d*_6_) δ 8.30 (s, 1H), 7.92 (br.s, 3H), 7.08 (s, 2H), 6.56 (s, 1H), 4.15–4.04 (m, 2H), 2.87–2.76 (m, 2H), 2.76–2.66 (m, 2H), 2.19 (s, 6H), 1.73–1.60 (m, 2H), 1.60–1.45 (m, 3H), 1.12–0.96 (m, 2H). ^13^C NMR (101 MHz, DMSO-*d*_6_) δ 154.9, 140.5, 137.0, 123.1, 117.4, 43.9, 36.4, 33.5, 32.6, 31.5, 21.1. HRMS (ESI) calcd for C_16_H_26_N_3_O^+^ [M+H^+^] 276.2070; found 276.2071.

*(14) 4-(2-Aminoethyl)-N-(2,5-dimethylphenyl)piperidine-1-carboxamide hydrochloride (***19***)*. Yield 80 mg (51%). ^1^H NMR (400 MHz, DMSO-*d*_6_) δ 8.00–7.78 (m, 4H), 7.06–7.00 (m, 1H), 6.98 (s, 1H), 6.88–6.79 (m, 1H), 4.14–3.99 (m, 2H), 2.89–2.68 (m, 4H), 2.23 (s, 3H), 2.09 (s, 3H), 1.72–1.62 (m, 2H), 1.61–1.46 (m, 3H), 1.14–0.98 (m, 2H). ^13^C NMR (126 MHz, DMSO-*d*_6_) δ 155.5, 137.9, 134.6, 129.9, 129.8, 126.6, 125.1, 44.0, 36.4, 33.6, 32.7, 31.5, 20.5, 17.5. HRMS (ESI) calcd for C_16_H_26_N_3_O^+^ [M+H^+^] 276.2070; found 276.2071.

*(15) 4-(2-Aminoethyl)-N-(2,3-dimethylphenyl)piperidine-1-carboxamide hydrochloride (***20***)*. Yield 75 mg (48%). ^1^H NMR (400 MHz, DMSO-*d*_6_) δ 8.70 (s, 1H), 8.11–7.76 (m, 3H), 7.07–6.84 (m, 3H), 4.15–4.00 (m, 2H), 2.96–2.65 (m, 4H), 2.23 (s, 3H), 2.02 (s, 3H), 1.74–1.47 (m, 5H), 1.16–0.99 (m, 2H). ^13^C NMR (101 MHz, DMSO-*d*_6_) δ 155.8, 138.0, 136.4, 132.3, 126.2, 124.8, 124.3, 44.0, 36.4, 33.6, 32.7, 31.5, 20.2, 18.5. HRMS (ESI) calcd for C_16_H_26_N_3_O^+^ [M+H^+^] 276.2070; found 276.2075.

*(16) 4-(2-Aminoethyl)-N-(3-(trifluoromethyl)phenyl)piperidine-1-carboxamide hydrochloride (***21***)*. Yield 81 mg (46%). ^1^H NMR (400 MHz, DMSO-*d*_6_) δ 8.88 (s, 1H), 8.11–7.85 (m, 4H), 7.80–7.71 (m, 1H), 7.49–7.38 (m, 1H), 7.28–7.18 (m, 1H), 4.22–4.06 (m, 2H), 2.94–2.69 (m, 4H), 1.76–1.63 (m, 2H), 1.63–1.46 (m, 3H), 1.15–0.98 (m, 2H). ^13^C NMR (101 MHz, DMSO-*d*_6_) δ 154.5, 141.7, 129.4, 129.1 (q, J = 31.2 Hz), 124.3 (q, J = 272.0 Hz), 122.8 (d, J = 1.1 Hz), 117.6 (q, J = 3.9 Hz), 115.4 (q, J = 4.0 Hz), 43.9, 36.5, 33.5, 32.6, 31.4. HRMS (ESI) calcd for C_15_H_21_F_3_N_3_O^+^ [M+H^+^] 316.1631; found 316.1629.

*(17) 4-(2-Aminoethyl)-N-(2-methoxy-5-methylphenyl)piperidine-1-carboxamide hydrochloride (***22***)*. Yield 95 mg (58%). ^1^H NMR (400 MHz, DMSO-*d*_6_) δ 7.93 (br.s, 3H), 7.60–7.40 (m, 2H), 6.89–6.83 (m, 1H), 6.82–6.75 (m, 1H), 4.06–3.96 (m, 2H), 3.76 (s, 3H), 2.87–2.71 (m, 4H), 2.20 (s, 3H), 1.74–1.61 (m, 2H), 1.61–1.45 (m, 3H), 1.14–0.99 (m, 2H). ^13^C NMR (101 MHz, DMSO-*d*_6_) δ 154.7, 147.8, 128.8, 128.4, 123.2, 122.7, 110.7, 55.8, 43.9, 36.4, 33.5, 32.5, 31.3, 20.5. HRMS (ESI) calcd for C_16_H_26_N_3_O_2_^+^ [M+H^+^] 292.2020; found 292.2021.

*(18) 4-(2-Aminoethyl)-N-(3-chlorophenyl)piperidine-1-carboxamide hydrochloride (***23***)*. Yield 71 mg (45%). ^1^H NMR (300 MHz, DMSO-*d*_6_) δ 8.79 (s, 1H), 8.08 (br.s, 3H), 7.68 (s, 1H), 7.48–7.38 (m, 1H), 7.27–7.16 (m, 1H), 6.99–6.88 (m, 1H), 4.22–4.04 (m, 2H), 2.88–2.66 (m, 4H), 1.74–1.47 (m, 5H), 1.14–0.95 (m, 2H). ^13^C NMR (75 MHz, DMSO-*d*_6_) δ 154.5, 142.5, 132.6, 129.8, 121.0, 118.8, 117.7, 43.9, 36.4, 33.4, 32.6, 31.5. HRMS (ESI) calcd for C_14_H_21_ClN_3_O^+^ [M+H^+^] 282.1368; found 282.1366.

#### 3.1.7. Synthesis of Benzyl (2-(1-Benzoylpiperidin-4-yl)ethyl)carbamates **24**–**25**

Et_3_N (61 mg, 0.6 mmol) was added to the stirred ice-cold solution of benzyl (2-(piperidin-4-yl)ethyl)carbamate **2** (Mixture A, approximately 0.5 mmol) in CH_2_Cl_2_ (4 mL), followed by dropwise addition of corresponding benzoyl chloride (0.55 mmol). The reaction mixture was allowed to warm up to RT and stirred for 1 h. The solution was diluted with CH_2_Cl_2_ (6 mL) and mixed with 1M HCl solution (10 mL). The organic phase was separated, dried over Na_2_SO_4_, filtered and concentrated in vacuo to give crude product which was purified by column chromatography.

The Cbz-protected compound was dissolved in MeOH (15 mL). The solution was purged with argon followed by addition of 10% Pd/C (3 mol % of Pd). The resulting suspension was flashed with argon and hydrogen, and left stirred under the atmosphere of hydrogen gas (1 atm) for 24 h at RT. The reaction mixture was filtered through a pad of celite to remove catalyst and concentrated to provide crude product which was purified by acid/base extraction. The product was dissolved in a small amount of EtOAc and mixed with required amount of HCl solution in EtOAc to give stable at ambient conditions hydrochloride salt.

*(1) (4-(2-Aminoethyl)piperidin-1-yl)(phenyl)methanone hydrochloride (***24***)*. Yield 79 mg (59%). ^1^H NMR (400 MHz, DMSO-*d*_6_) δ 7.96 (br.s, 3H), 7.47–7.40 (m, 3H), 7.40–7.32 (m, 2H), 4.46 (br.s, 1H), 3.55 (br.s, 1H), 3.09–2.65 (m, 4H), 1.84–1.46 (m, 5H), 1.19–0.99 (m, 2H). ^13^C NMR (101 MHz, DMSO-*d*_6_) δ 168.9, 136.4, 129.3, 128.4, 126.6, 47.1 (br.s), 41.5 (br.s), 36.3, 33.3, 32.6, 31.8, 31.2. HRMS (ESI) calcd for C_14_H_21_N_2_O^+^ [M+H^+^] 233.1648; found 233.1644.

*(2) (4-(2-Aminoethyl)piperidin-1-yl)(m-tolyl)methanone (***25***)*. Yield 82 mg (58%). ^1^H NMR (400 MHz, DMSO-*d*_6_) δ 7.91 (br.s, 3H), 7.35–7.28 (m, 1H), 7.28–7.22 (m, 1H), 7.19–7.10 (m, 2H), 4.45 (br.s, 1H), 3.56 (br.s, 1H), 3.08–2.64 (m, 4H), 2.33 (s, 3H), 1.83–1.45 (m, 5H), 1.16–0.99 (m, 2H). ^13^C NMR (101 MHz, DMSO-*d*_6_) δ 169.0, 137.7, 136.4, 129.8, 128.2, 127.1, 123.6, 47.0 (br.s), 41.4 (br.s), 36.3, 33.3, 32.6, 31.8, 31.1, 20.9. HRMS (ESI) calcd for C_15_H_23_N_2_O^+^ [M+H^+^] 247.1805; found 247.1805.

#### 3.1.8. Synthesis of Benzyl (2-(1-(Arylsulfonyl)piperidin-4-yl)ethyl)carbamates **26**–**28**

Triethylamine (56 mg, 0.550 mmol), and a catalytic amount of 4-CH2Cl2 (DMAP) were added to a solution of benzyl (2-(piperidin-4-yl)ethyl)carbamate **2** (Mixture A, approximately 0.5 mmol) in CH_2_Cl_2_ (2 mL). After stirring at RT for 15 min, a solution of corresponding sulfonyl chloride (0.550 mmol) in dichloromethane (2 mL) was added slowly in dropwise manner. The reaction mixture was stirred at 40 °C under inert atmosphere for 12 h. After complete conversion, the reaction mixture was neutralized with saturated sodium bicarbonate solution, and the aqueous layer was extracted with EtOAc, washed with H_2_O, dried over Na_2_SO_4_, filtered and concentrated in vacuo. The residue was purified by flash chromatography (eluent: EtOAc + hexane).

The Cbz-protected compound was dissolved in MeOH (15 mL). The solution was purged with argon followed by addition of 10% Pd/C (3 mol % of Pd). The resulting suspension was flashed with argon and hydrogen, and left stirred under the atmosphere of hydrogen gas (1 atm) for 24 h at RT. The reaction mixture was filtered through a pad of celite to remove catalyst and concentrated to provide crude product which was purified by acid/base extraction. The product was dissolved in a small amount of EtOAc and mixed with required amount of HCl solution in EtOAc to give stable at ambient conditions hydrochloride salt.

*(1) 2-[1-(Phenylsulfonyl)piperidin-4-yl]ethanamine hydrochloride (***26***)*. Yield 85 mg (56%). ^1^H NMR (300 MHz, DMSO-*d*_6_) δ 8.08 (br.s, 3H), 7.77–7.60 (m, 5H), 3.66–3.56 (m, 2H), 2.79–2.64 (m, 2H), 2.23–2.07 (m, 2H), 1.75–1.63 (m, 2H), 1.52–1.38 (m, 2H), 1.38–1.21 (m, 1H), 1.21–1.05 (m, 2H). ^13^C NMR (75 MHz, DMSO-*d*_6_) δ 135.4, 133.1, 129.4, 127.4, 46.0, 36.2, 32.9, 31.4, 30.5. HRMS (ESI) calcd for C_13_H_21_N_2_O_2_S^+^ [M+H^+^] 269.1318; found 269.1317.

*(2) 2-{1-[(4-Fluorophenyl)sulfonyl]piperidin-4-yl}ethanamine hydrochloride (***27***)*. Yield 125 mg (78%). ^1^H NMR (300 MHz, DMSO-*d*_6_) δ 8.05 (br.s, 3H), 7.85–7.76 (m, 2H), 7.54–7.44 (m, 2H), 3.67–3.53 (m, 2H), 2.81–2.64 (m, 2H), 2.24–2.09 (m, 2H), 1.76–1.63 (m, 2H), 1.53–1.39 (m, 2H), 1.39–1.22 (m, 1H), 1.22–1.05 (m, 2H). ^13^C NMR (75 MHz, DMSO-*d*_6_) δ 164.6 (d, *J* = 251.7 Hz), 131.9 (d, *J* = 2.9 Hz), 130.5 (d, *J* = 9.6 Hz), 116.6 (d, *J* = 22.6 Hz), δ 45.9, 36.2, 32.9, 31.4, 30.5. HRMS (ESI) calcd for C_13_H_20_FN_2_O_2_S^+^ [M+H^+^] 287.1224; found 287.1224.

*(3) 2-{1-[(4-Methylphenyl)sulfonyl]piperidin-4-yl}ethanamine hydrochloride (***28***)*. Yield 115 mg (72%). ^1^H NMR (300 MHz, DMSO-*d*_6_) δ 8.05 (br.s, 3H), 7.61 (d, *J* = 8.2 Hz, 2H), 7.44 (d, *J* = 8.1 Hz, 2H), 3.64–3.52 (m, 2H), 2.80–2.64 (m, 2H), 2.40 (s, 3H), 2.19–2.04 (m, 2H), 1.74–1.62 (m, 2H), 1.51–1.38 (m, 2H), 1.36–1.21 (m, 1H), 1.21–1.04 (m, 2H). ^13^C NMR (75 MHz, DMSO-*d*_6_) δ 143.5, 132.4, 129.8, 127.5, 45.9, 36.2, 32.9, 31.4, 30.5, 21.0. HRMS (ESI) calcd for C_14_H_23_N_2_O_2_S^+^ [M+H^+^] 283.1475; found 283.1474.

#### 3.1.9. Synthesis of 2-(1-Methylpiperidin-4-yl)ethan-1-amine dihydrochloride (**29**)

Synthesis of 2-(1-Methylpiperidin-4-yl)ethan-1-amine dihydrochloride (**29**) from 1-methylpiperidin-4-one (0.5 mmol scale) was achieved using the same procedures as for compounds **1**, **7–23**. The overall yield for three steps 47 mg (44%). ^1^H NMR (300 MHz, DMSO-*d*_6_) δ 10.78 (br.s, 1H), 8.19 (br.s, 3H), 3.37–3.26 (m, 2H), 2.93–2.72 (m, 4H), 2.66 (s, 3H), 1.87–1.75 (m, 2H), 1.65–1.37 (m, 5H). ^13^C NMR (75 MHz, DMSO-*d_6_*) δ 53.2, 42.5, 36.3, 32.8, 29.9, 28.6. HRMS (ESI) calcd for C_8_H_19_N_2_^+^ [M+H^+^] 143.1543; found 143.1544.

### 3.2. BRET Analysis

The Bioluminescence Resonance Energy Transfer approach (BRET) has been used for functional activity experiments. HEK-293 cells were transiently transfected with cDNA for TAARs and cAMP BRET biosensor (EPAC) protein and then plated in a 96-well plate as described [18]. All compounds were tested at the initial concentrations of 1 μM or 10 μM. Then, for active compounds, a concentration–response analysis was performed in order to calculate the EC_50_ values. Natural agonist of TAAR1 tyramine hydrochloride was used as a positive control. 

### 3.3. Molecular Modeling 

#### 3.3.1. Protein Structure Preparation

Protein structure is unavailable in the Protein Data Bank. A TAAR1 structure modeled with AlphaFold [22] software was downloaded from Uniprot database [23] (structure ID Q96RJ0). The protein model thus obtained was preprocessed with use of protein preparation wizard, included in the Schrödinger Suite (NY, USA, version 2021-4). In this step, the following errors in protein model were eliminated: invalid bond orders, protonation states, atom typing, missing amino acid sidechains, missing loops, clashes between sidechains, incorrect torsions, etc. [24]. All operations with proteins and ligands were performed in OPLS47 forcefield, a part of the Schrödinger Suite.

#### 3.3.2. Ligand Structure Preparation

Three-dimensional structures of ligands were generated in the same forcefield—OPLS4 [25]. For all ligands, pKa values were calculated and likely protonation states determined with the use of Epik [26] module of the Schrödinger Suite.

#### 3.3.3. Molecular Docking Procedure

Protein-ligand complexes with TAAR1 were obtained with the use of Glide [27] methodology of molecular docking. The gridbox for molecular docking was centered on following TAAR1 residues: D103, I104, S107, V184, F186, T194, F195, F267, F268, I290 and Y294. Grid size was selected to be 16 × 16 × 16 Å. For each compound, 20 poses were generated.

#### 3.3.4. Molecular Docking: Best-Fitting Structure Selection

Best-fitting binding pose was selected based on the docking solutions clustering and interactions with the crucial amino acid residues as described in the literature [9,16,28], as well as based on the GlideScore and ΔG values.

#### 3.3.5. Molecular Mechanics with Generalized Born and Surface Area Solvation (MM-GBSA)

Protein structure refinement during the MM-GBSA [19] modeling was limited by 6Å of ligand poses with sidechain optimization. The calculated Gibbs free energy included all interaction terms and strain energy components.

### 3.4. In Vivo Evaluation of Compounds Activity on DAT-KO Rat Hyperlocomotion

#### 3.4.1. Animals

Twelve Wistar dopamine transporter knockout (DAT-KO) female rats were utilized for assessment of drugs’ activity on hyperlocomotion. Animals were maintained on a 12/12 light/dark cycle at the room temperature maintained between 20 °C and 23 °C with a relative humidity maintained between 30% and 70%. Chow and water were provided ad libitum for the duration of the studies.

#### 3.4.2. Compound Administration

All compounds were dissolved in physiological saline (0.9% solution of NaCl) and intraperitoneally injected in volume 10 mL/kg immediately before locomotion testing.

#### 3.4.3. Locomotion Tests

Rats were intraperitoneally injected with a compound solution or vehicle (saline) and were immediately placed in Plexiglas boxes 40 cm × 40 cm × 40 cm for 90 min. Noldus EthoVision software was used to measure the distance moved by the animals.

## 4. Conclusions

Starting from screening hit **1** with EC_50_ = 0.507 μM agonistic activity towards TAAR1, twenty-three analogs were synthesized and compared in activity to one another and to a small subset of benzamide and sulfonamide analogs (as well as one compound lacking an aromatic moiety on the nitrogen atom). Several structure–activity relationship generalizations have been drawn from the observed data, some of which were corroborated by molecular modeling against the crystal structure of TAAR1. Ureas were by far the lead chemotype, while benzamides were less potent and sulfonamides inactive. Additionally inactive was 2-(1-methylpiperidin-4-yl)ethan-1-amine hydrochloride (**29**), lacking a pharmacophorically important bulky aromatic group on the nitrogen atom. The four most active compounds (EC_50_ for TAAR1 agonistic activity ranging from 0.033 to 0.112 μM) were nominated and scaled up for evaluation in vivo. The DAT-KO rat model of hyperlocomotion was used to evaluate the compounds’ efficacy in vivo. Out of four compounds, only compound **18** (AP163) displayed a statistically significant and dose-dependent reduction in hyperlocomotion in DAT-KO rats. As such, compound **18** (AP163) represents a viable lead for further preclinical characterization.

## Figures and Tables

**Table 1:** Agonistic activity of compounds **7**–**30** with respect to TAAR1.

Compound	Ar	Agonistic Activity *^a^* at 1 μM, %	EC_50_, μM
**1**	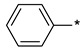	65	0.507
**7**	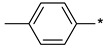	Inactive *^b^*	-
**8**	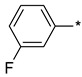	Inactive	-
**9**	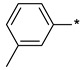	89	0.052
**10**	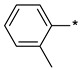	Inactive	-
**11**	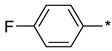	84	0.206
**12**	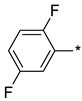	75	0.273
**13**	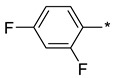	Inactive	-
**14**	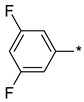	Inactive	-
**15**	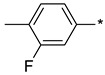	Inactive	inactive
**16**	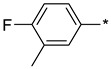	107 *^c^*	0.143
**17**	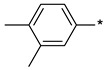	84 *^c^*	0.033
**18** (AP163)	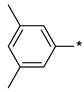	85 *^c^*	0.112
**19**	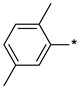	80 *^c^*	0.042
**20**	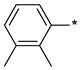	82 *^c^*	0.914
**21**	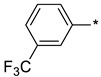	96 *^c^*	0.035
**22**	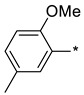	84*^c^*	1.90
**23**	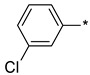	Inactive	-
**24**	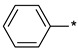	31 *^c^*	0.635
**25**	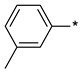	41 *^c^*	0.634
**26**	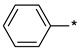	Inactive	-
**27**	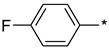	Inactive	-
**28**	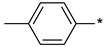	Inactive	-
**29**	-	Inactive	-

*^a^* Compared to 1 μM tyramine hydrochloride (positive control) on primary screening. *^b^* Producing less than 20% activation of TAAR1 relative to 1 μM tyramine hydrochloride. *^c^* Activity at 10 μM compared to 1 μM tyramine hydrochloride (positive control) on primary screening, *—joining bond.

**Table 2:** Docking score (GScore), MM-GBSA free energy of ligand binding (ΔG) and ‘correctness’ of ligand binding for triads **7**/**9**/**10** and **12**/**13**/**14** in comparison with Ralmitaront. Best binding poses are highlighted by green color.

Compound	GScore	ΔG	‘Correctness’ of Ligand Binding	TAAR1 Activity
Ralmitaront	−8.91	−36.85	Reference ligand	Active
**7**	−8.26	−30.93	Incorrect	Inactive
**9**	−8.26	−33.41	Fully correct	Active
**10**	−7.87	−29.50	Incorrect	Inactive
**12**	−8.26	−42.94	Fully correct	Active
**13**	−8.45	−27.27	Partially correct	Inactive
**14**	−5.92	−33.08	Incorrect	Inactive

## Data Availability

Not applicable.

## Supplementary Materials

The following supporting information can be downloaded at https://www.mdpi.com/article/10.3390/ijms231911579/s1: copies of ^1^H and ^13^C NMR spectra.
