# Supplementary material for: Discovery and In Vivo Efficacy of Trace Amine-Associated Receptor 1 (TAAR1) Agonist 4-(2-Aminoethyl)-N-(3,5-dimethylphenyl)piperidine-1-carboxamide Hydrochloride (AP163) for the Treatment of Psychotic Disorders
Source: Int J Mol Sci. 2022 Sep 30;23(19):11579. doi: 10.3390/ijms231911579 (PMC9569940; doi:10.3390/ijms231911579)
Supplement: Supplementary file 1 [file ijms-23-11579-s001.zip › ijms-1925623-supplementary.pdf]

Supplementary Information for

Discovery and In Vivo Efficacy of Trace Amine-Associated Receptor 1 (TAAR1) Agonist 4-(2-aminoethyl)-*N*-(3,5-dimethylphenyl)piperidine-1-carboxamide Hydrochloride (AP163) for the Treatment of Psychotic Disorders

Mikhail Krasavin*, Anatoly A. Peshkov, Alexey Lukin, Kristina Komarova, Lyubov Vinogradova, Daria Smirnova, Diana Gapanenok, Evgeny V. Kanov, Savelii R. Kuvarzin, Ramilya Z. Murtazina, Evgeniya V. Efimova, Maxim Gureev, Kirill Onokhin, Konstantin Zakharov and Raul R. Gainetdinov*

Contents

Copies of ^{1}H and ^{13}C NMR spectra for compounds **1, 7**-**29** 2-25

Calculated ADMET report from swissadme.ch for compound **18** 26-27

^{1}H and ^{13}C NMR spectra of compound **1**

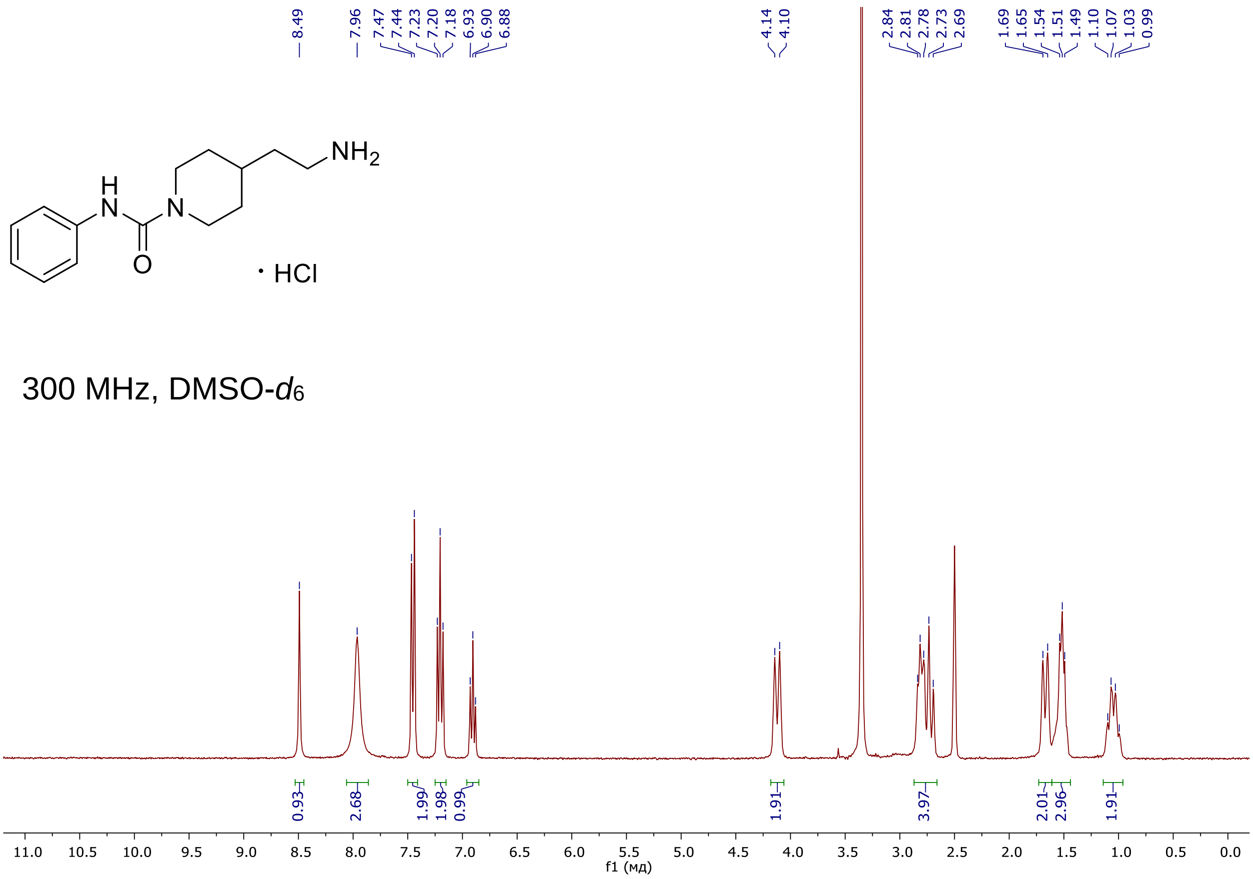

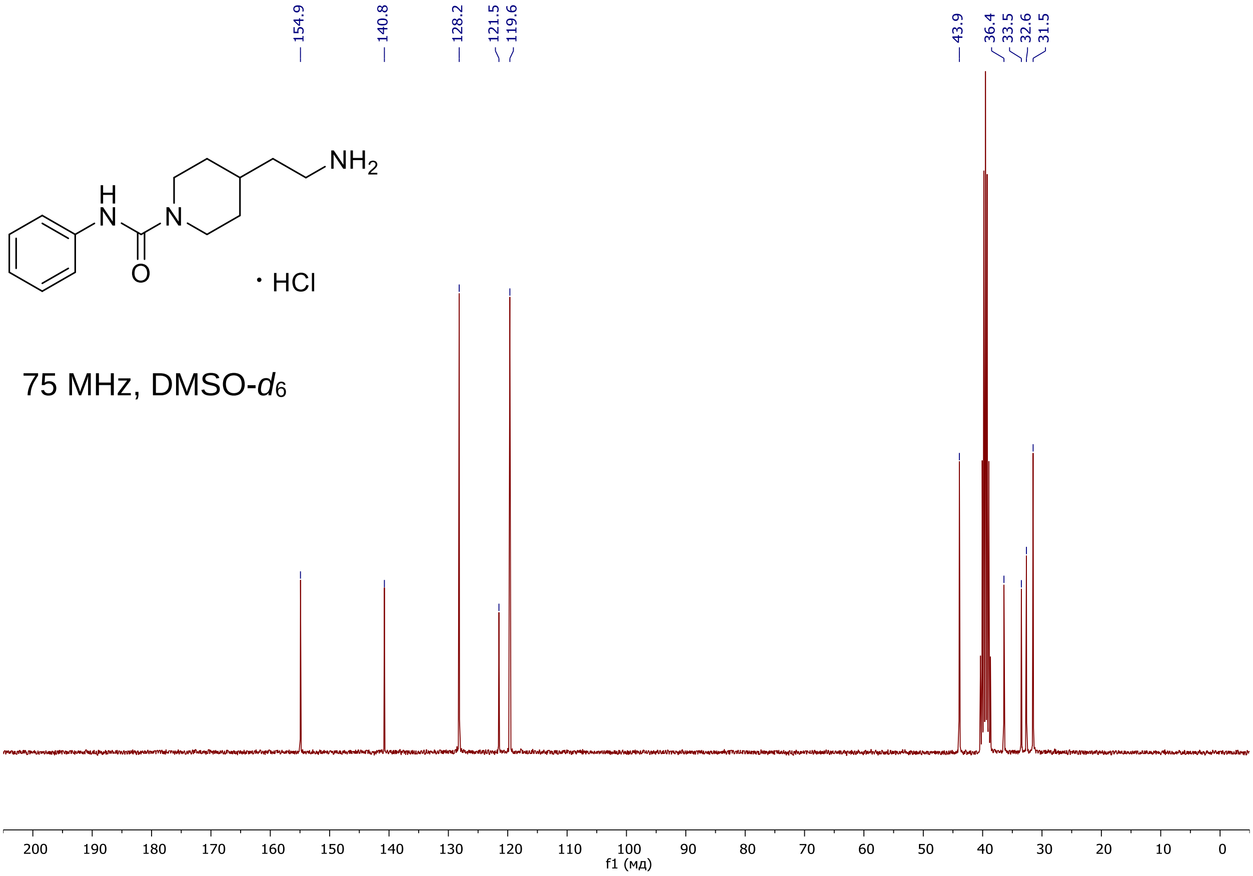

^{1}H and ^{13}C NMR spectra of compound **7**

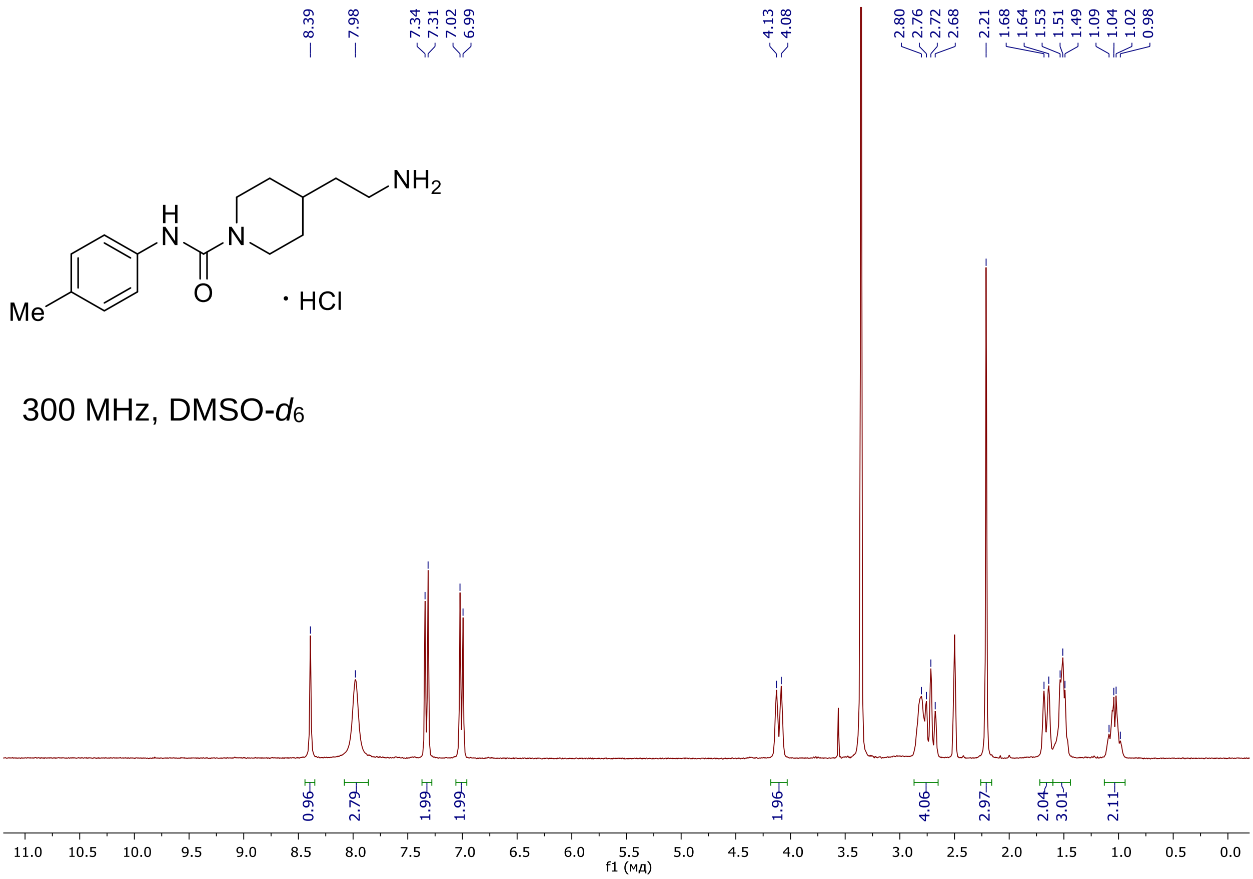

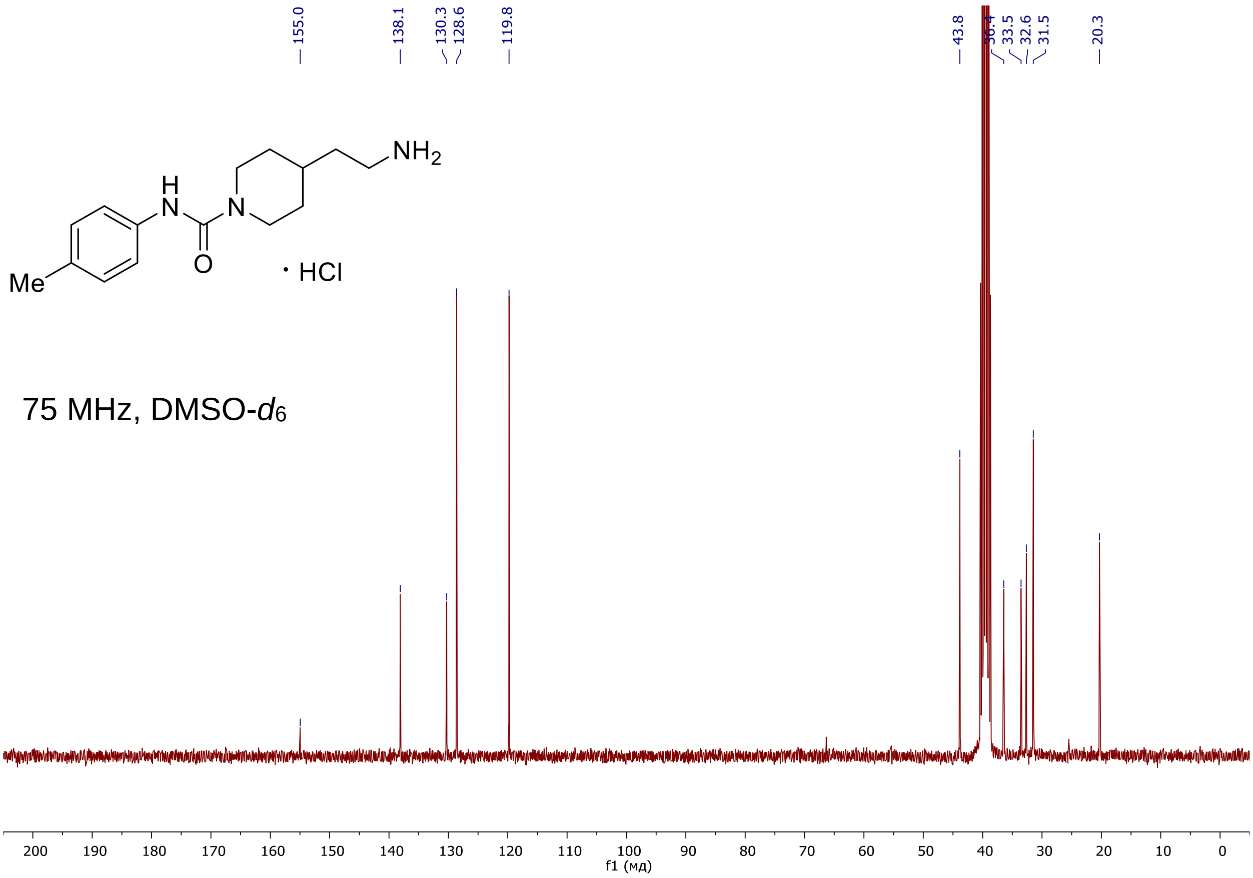

^{1}H and ^{13}C NMR spectra of compound **8**

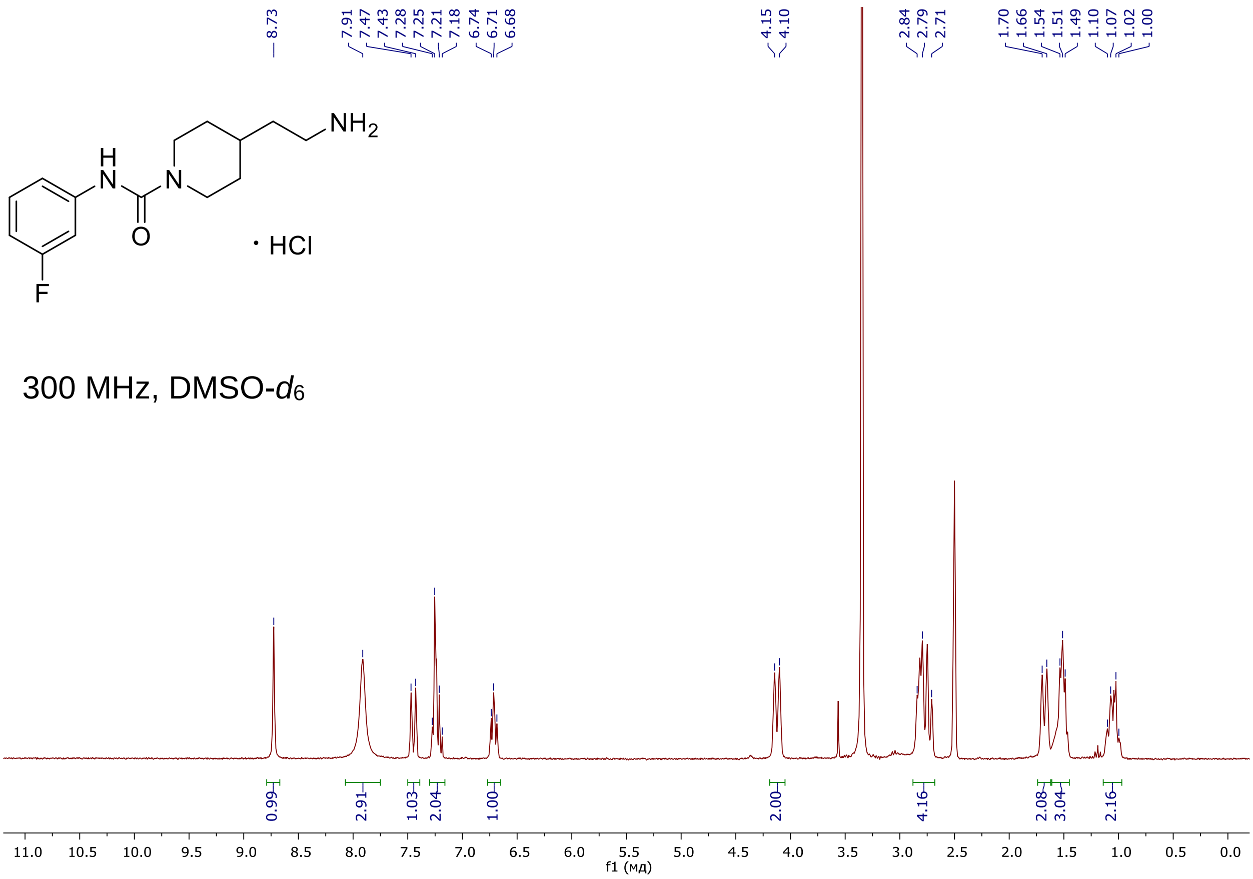

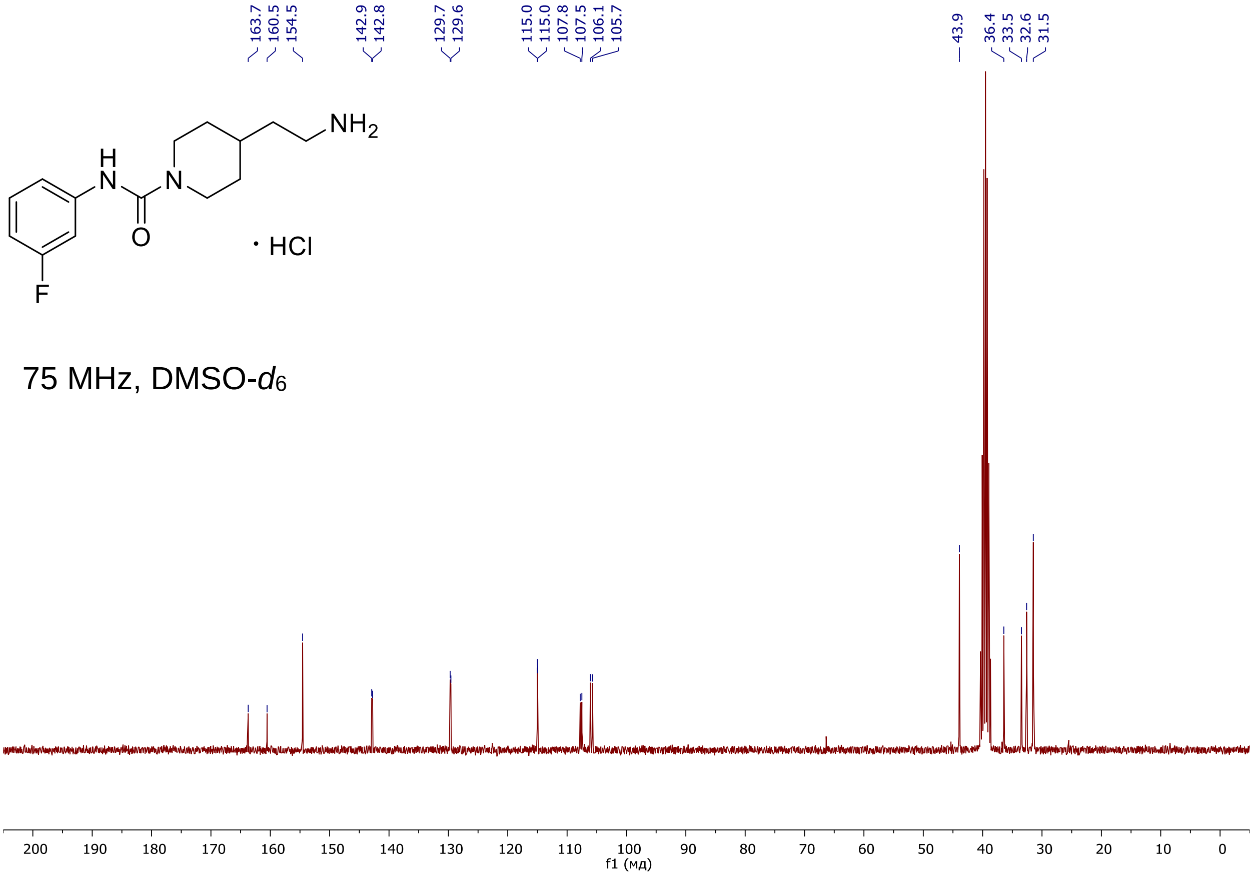

^{1}H and ^{13}C NMR spectra of compound **9**

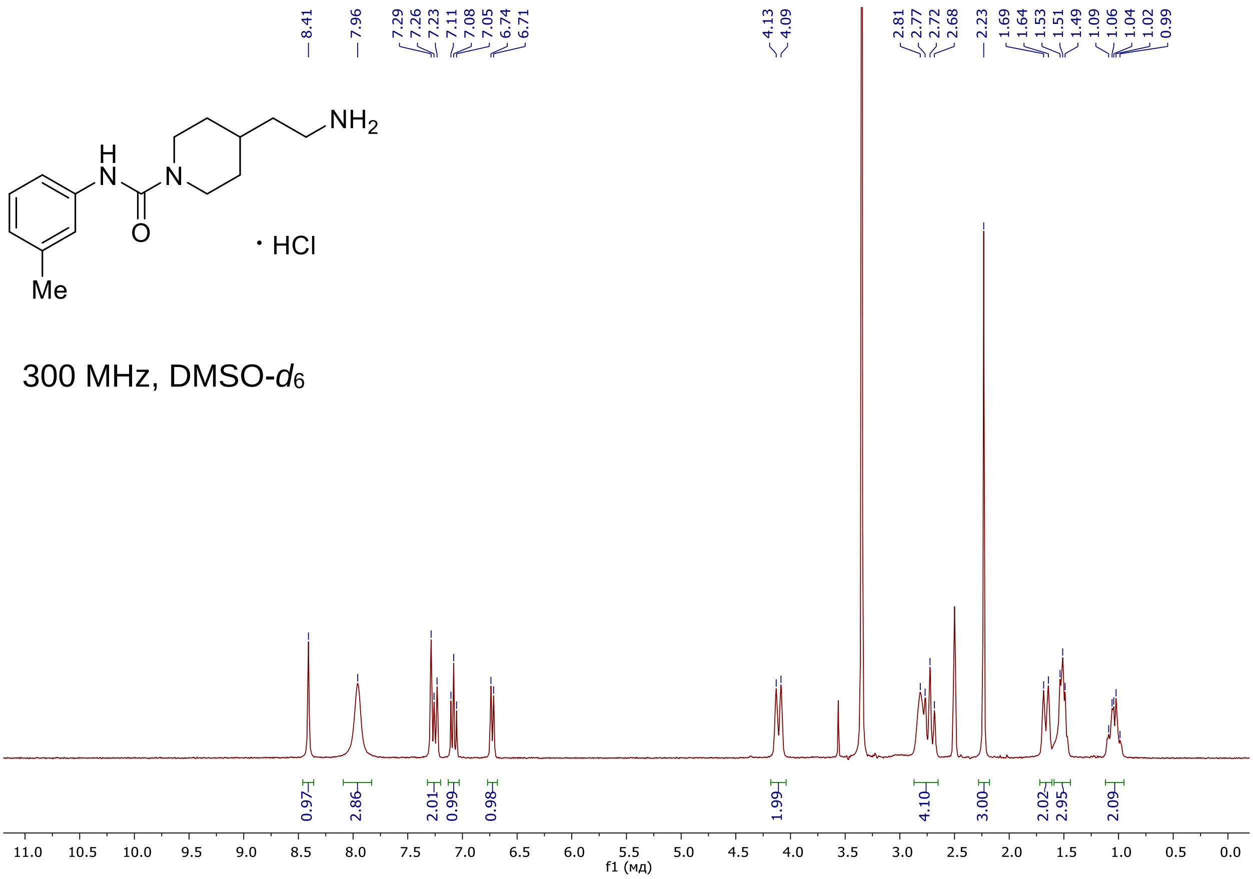

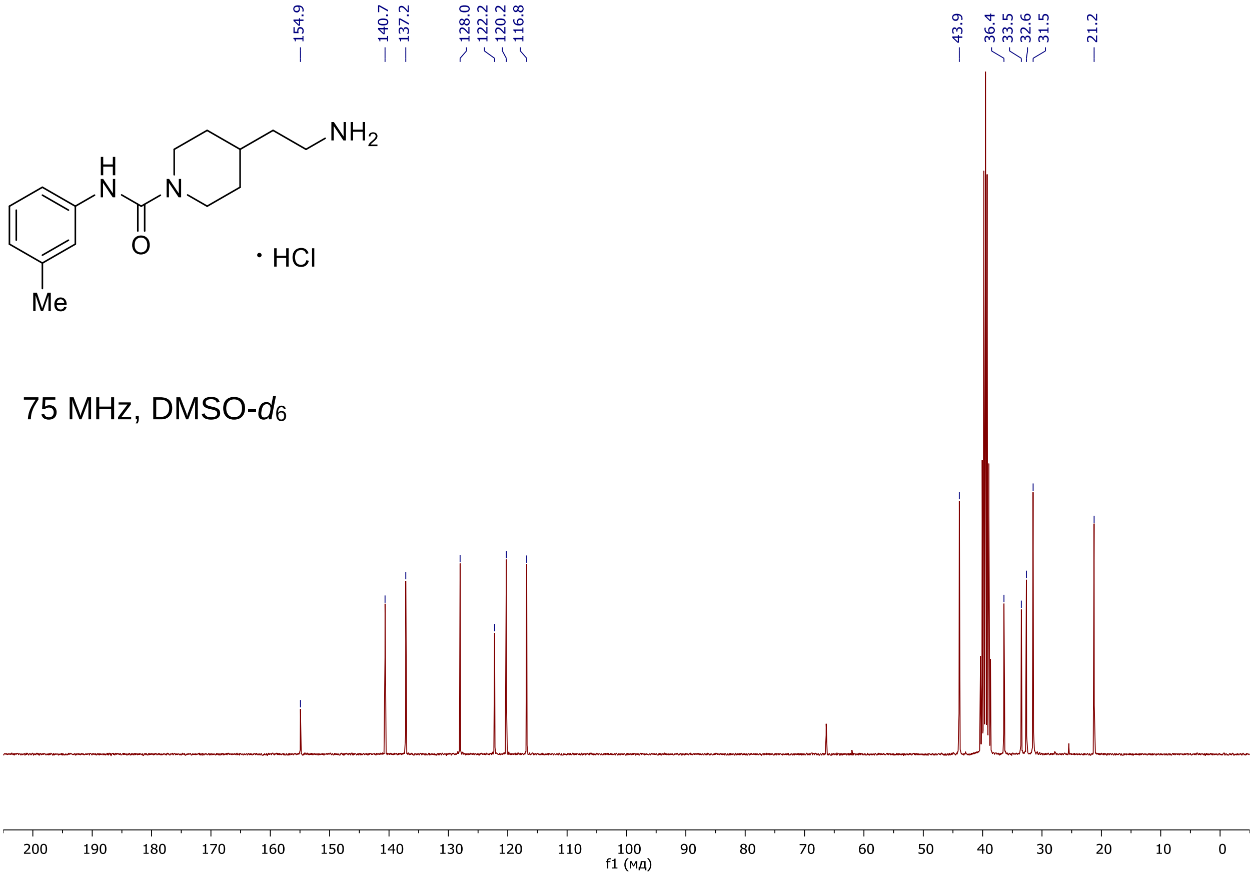

^{1}H and ^{13}C NMR spectra of compound **10**

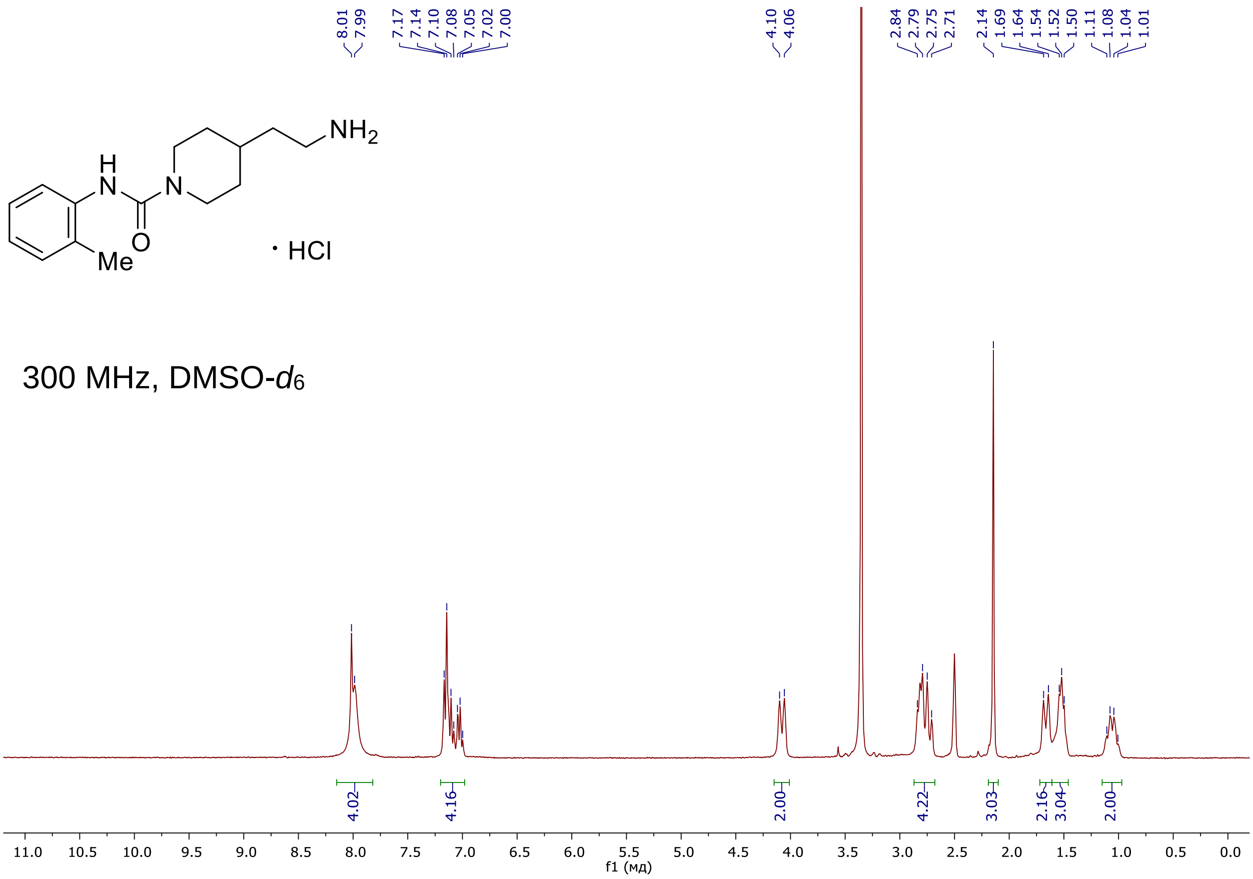

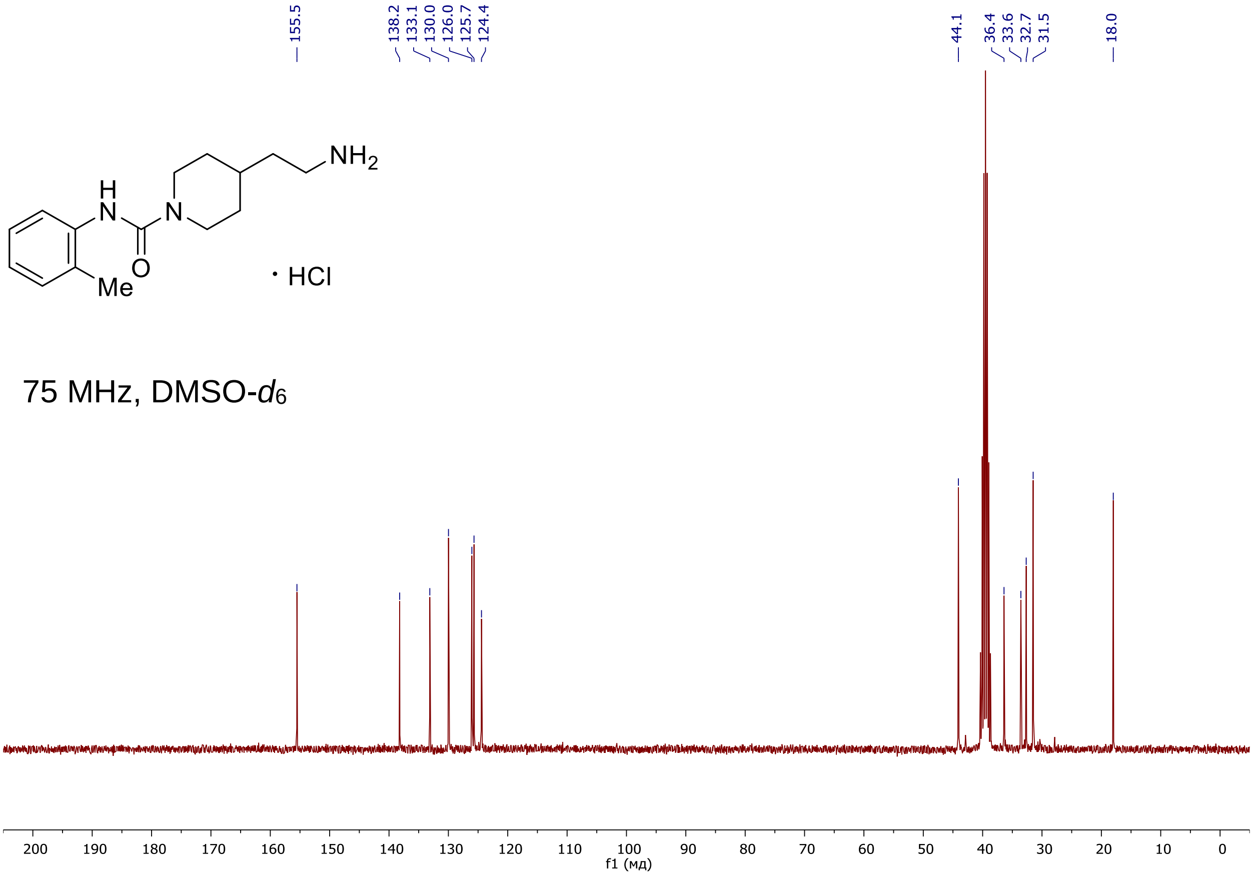

^{1}H and ^{13}C NMR spectra of compound **11**

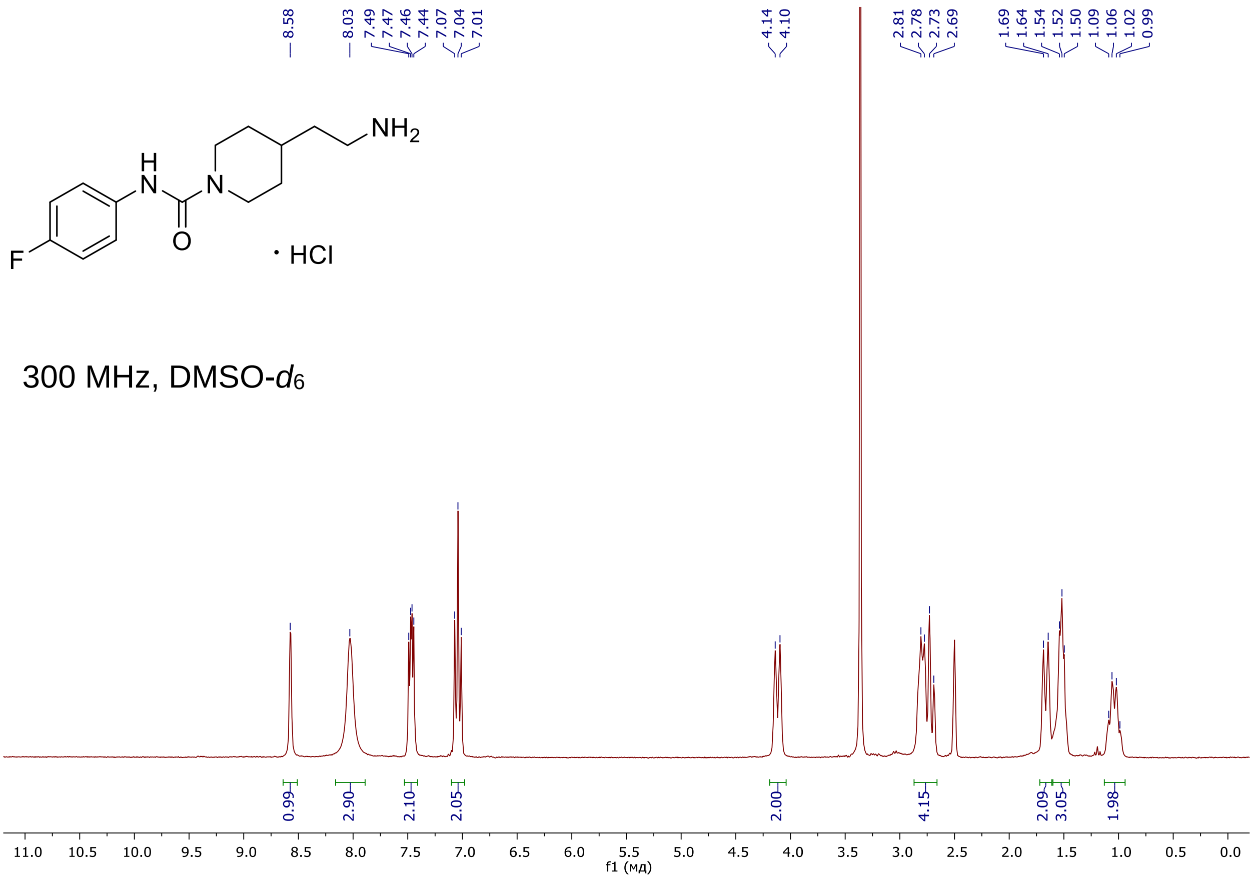

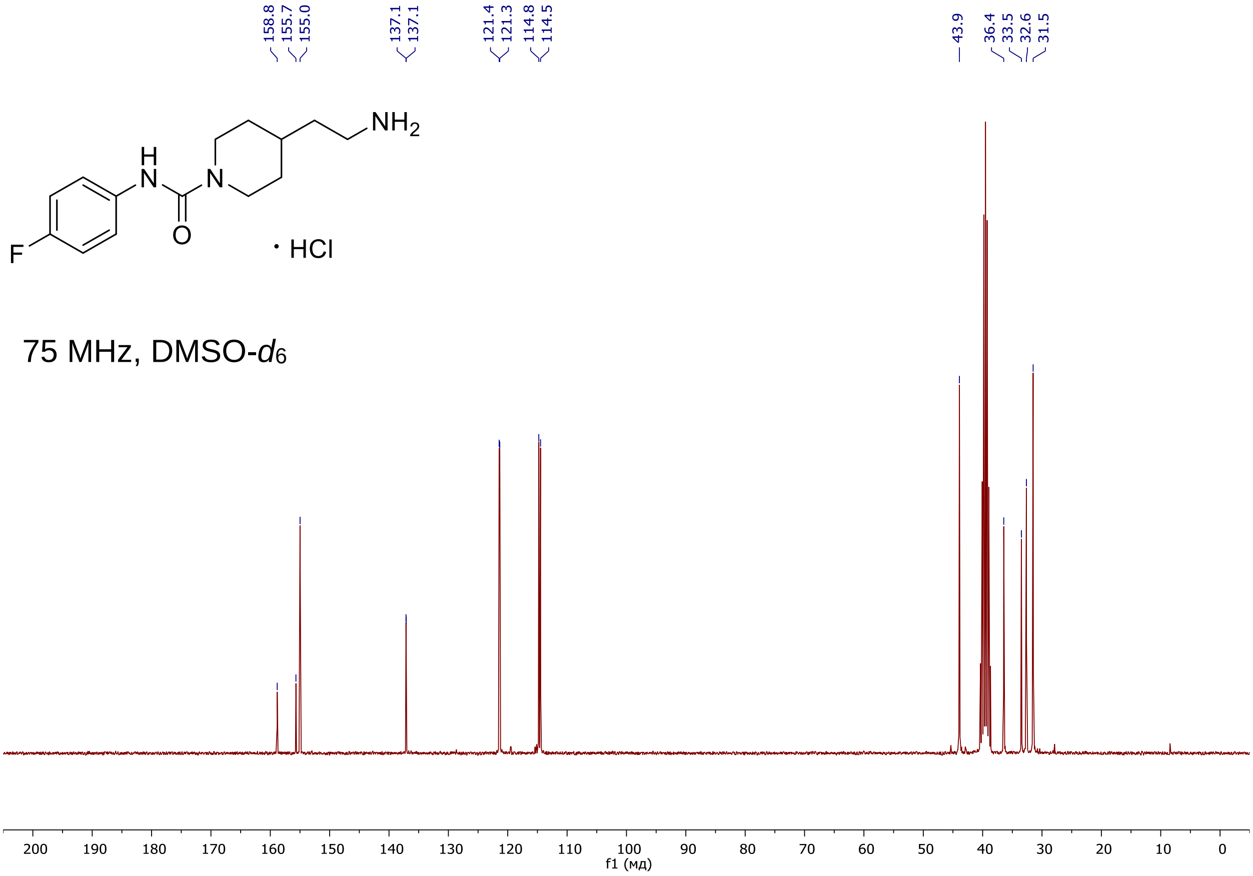

^{1}H and ^{13}C NMR spectra of compound **12**

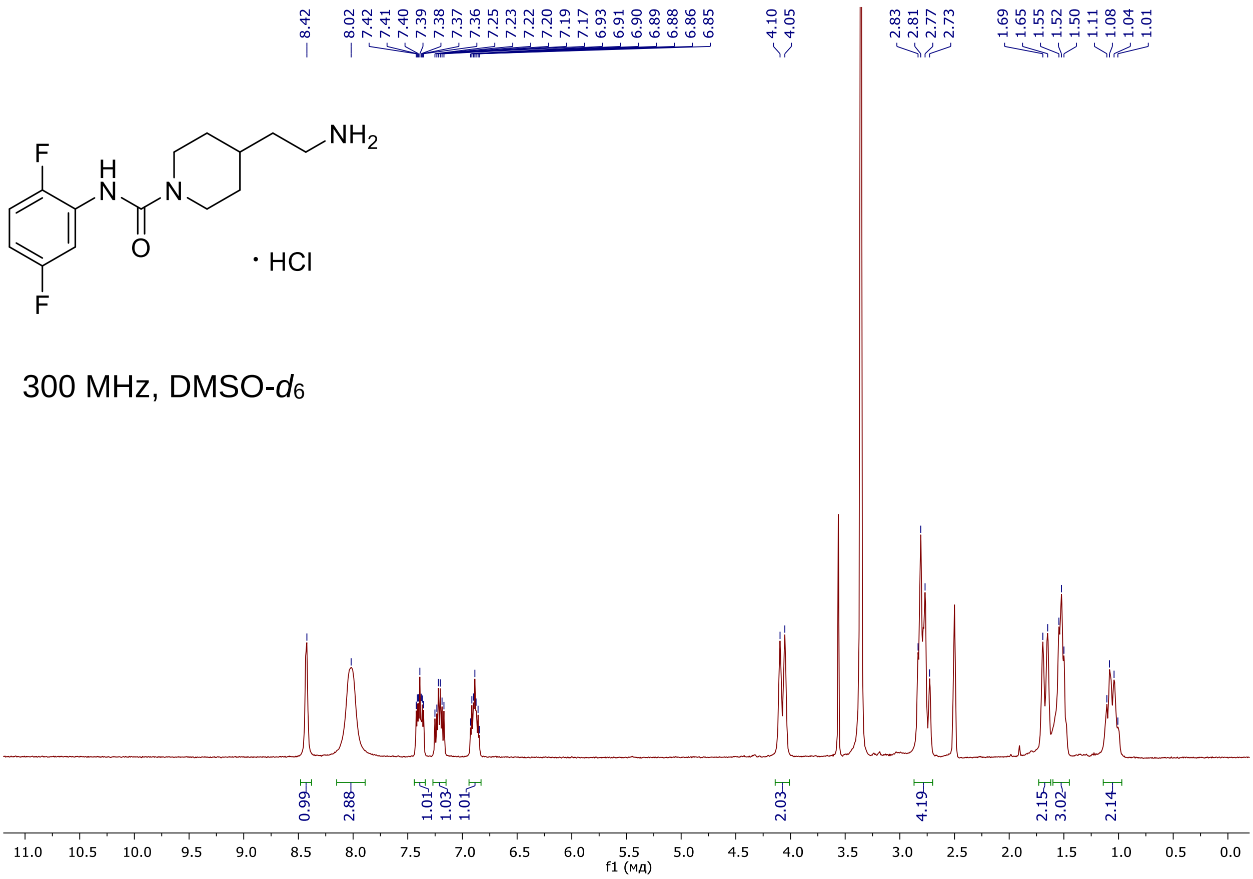

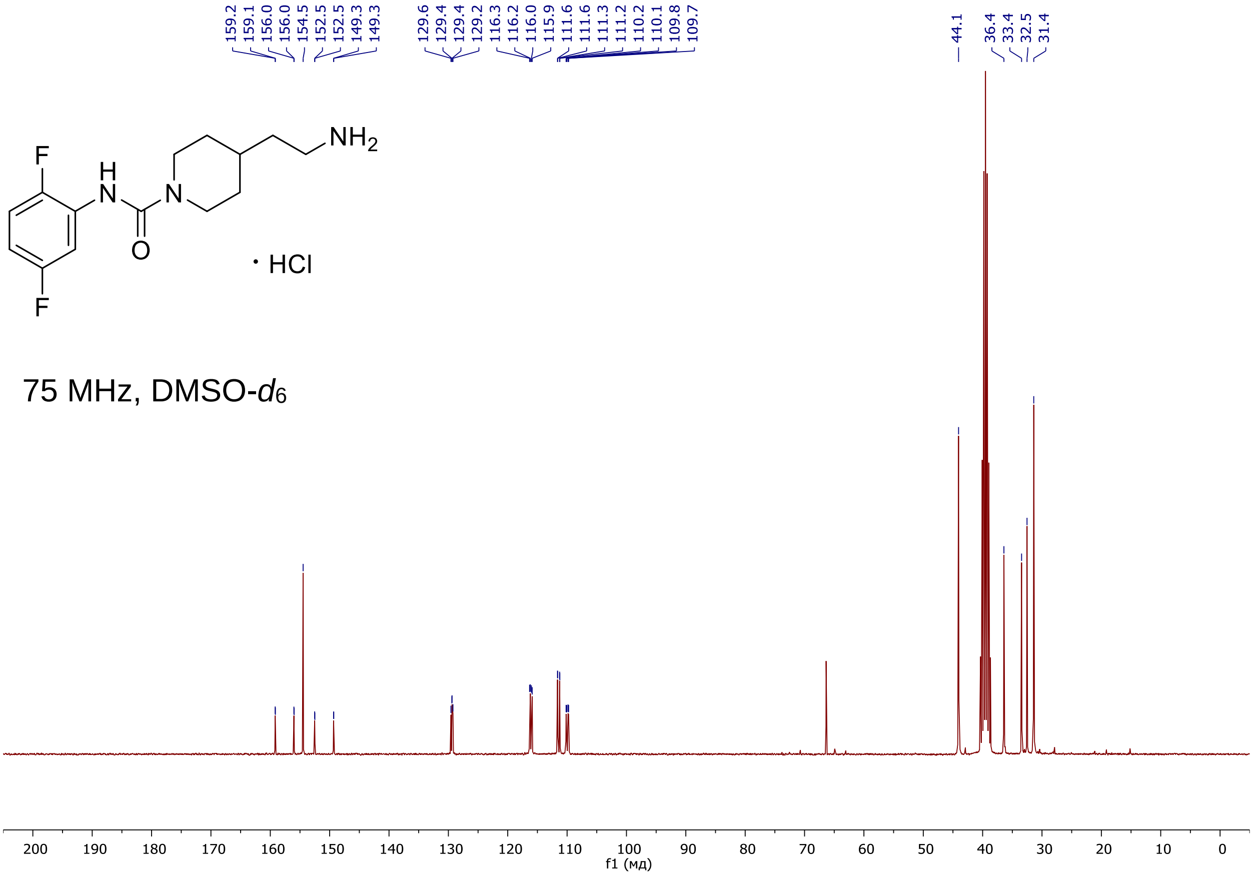

^{1}H and ^{13}C NMR spectra of compound **13**

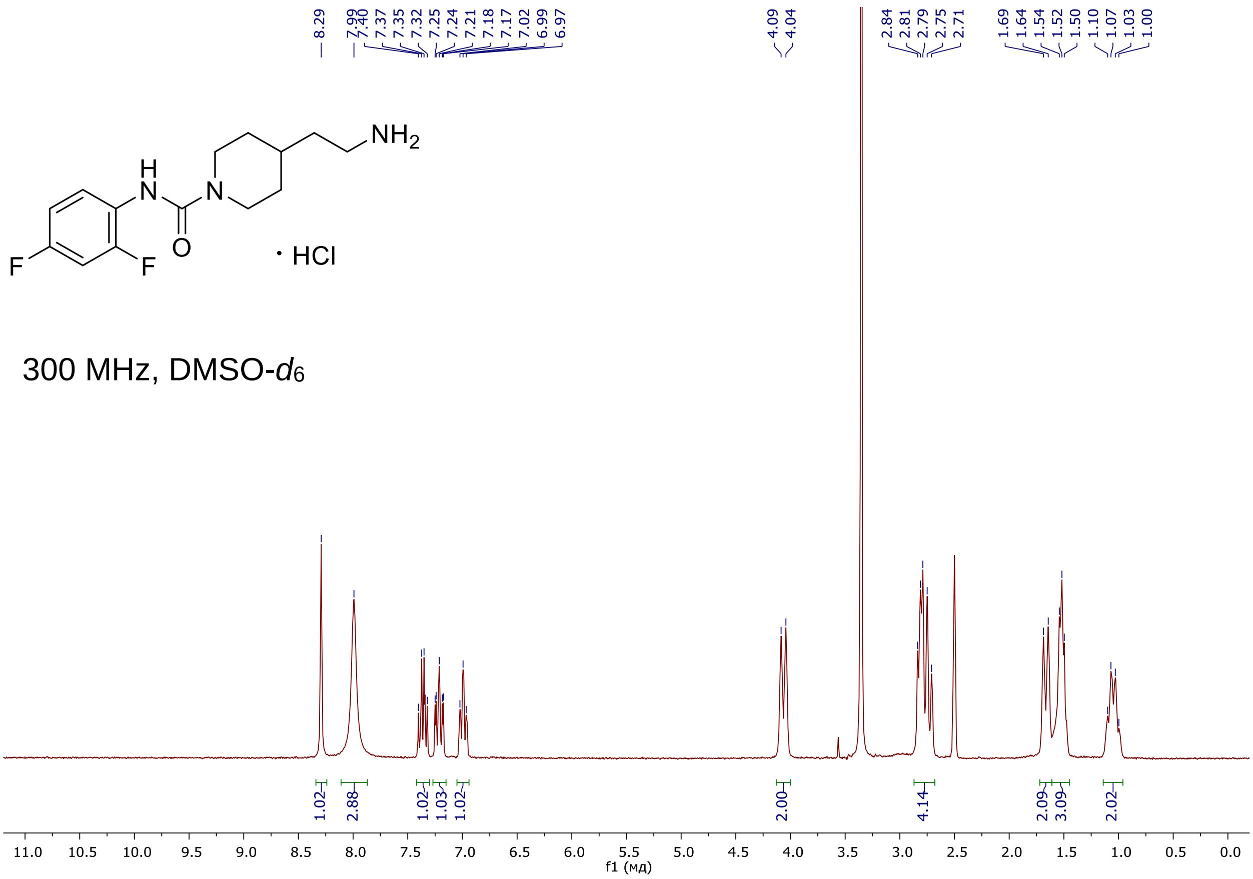

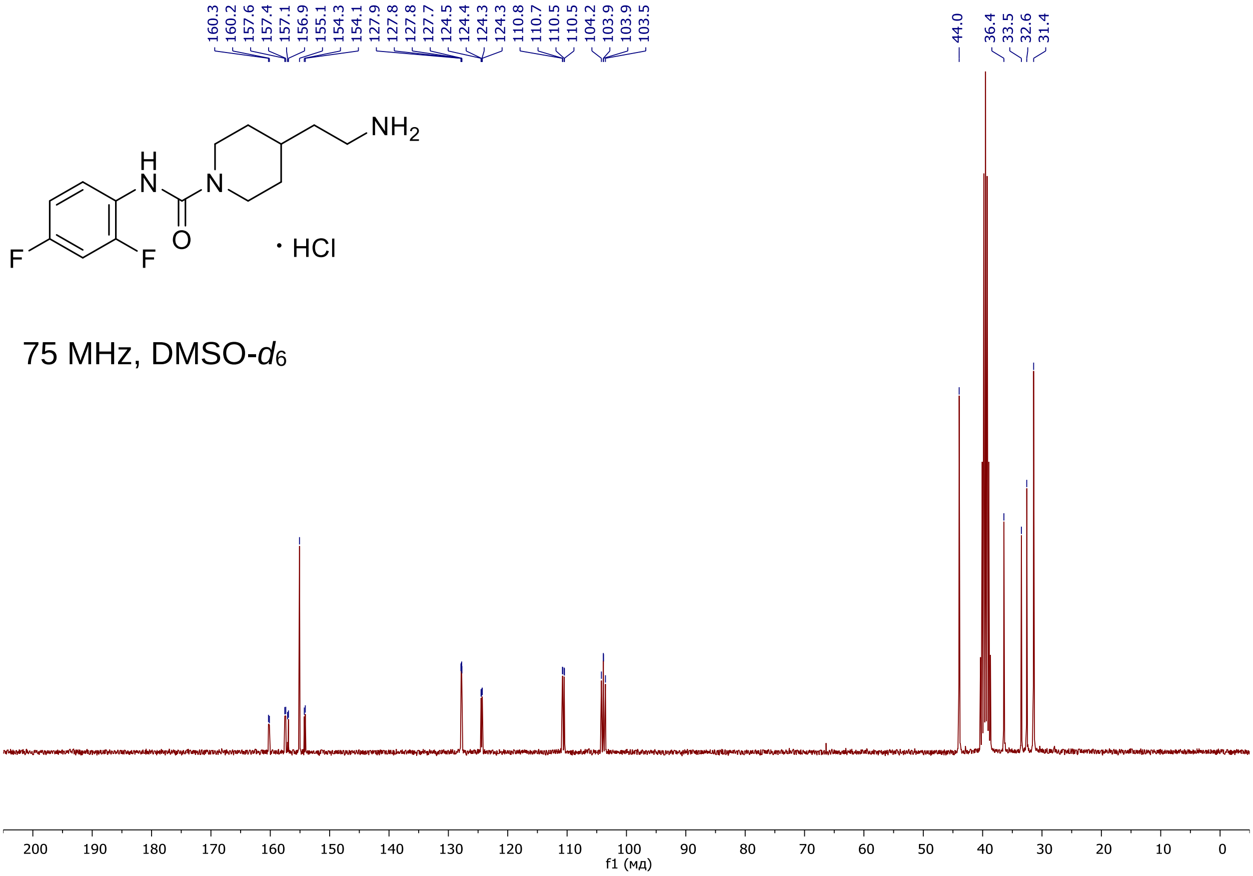

^{1}H and ^{13}C NMR spectra of compound **14**

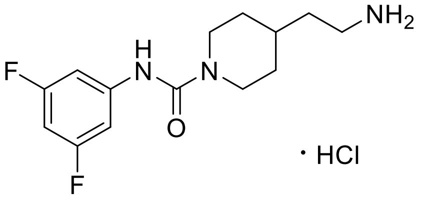

300 MHz, DMSO-d_6

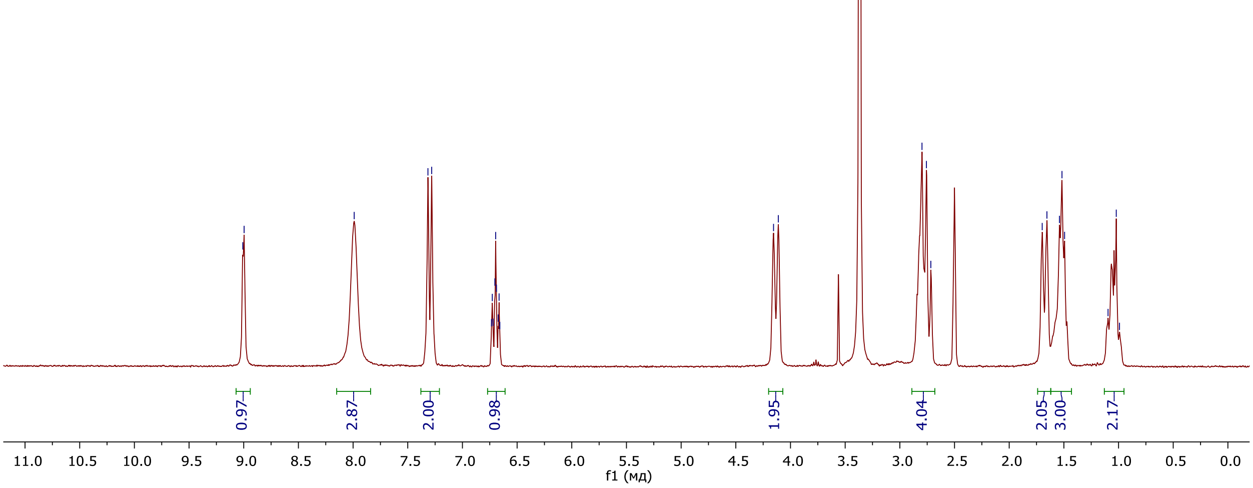

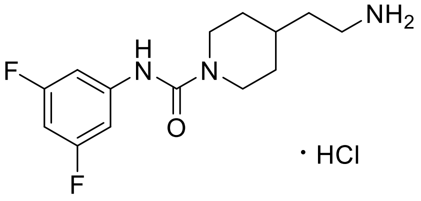

75 MHz, DMSO-d_6

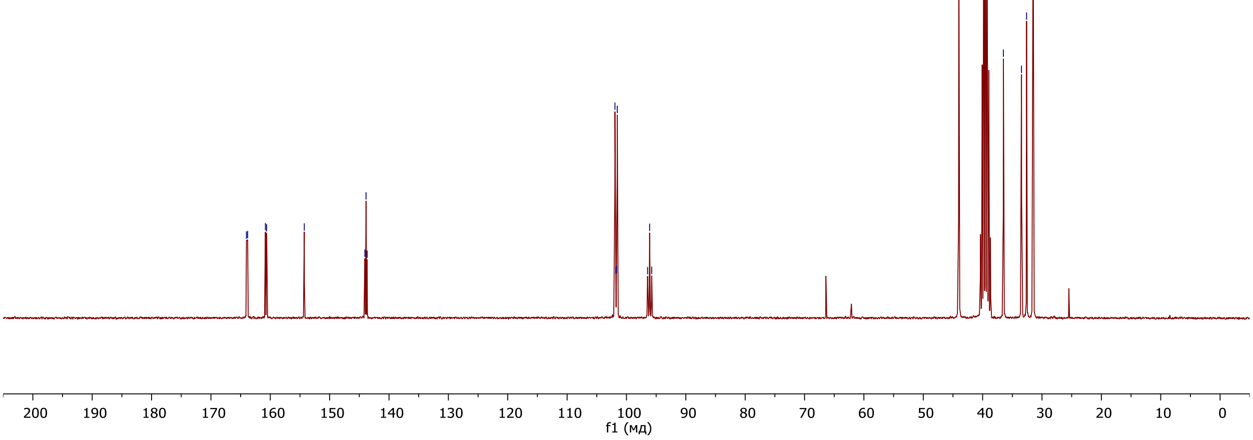

^{1}H and ^{13}C NMR spectra of compound **15**

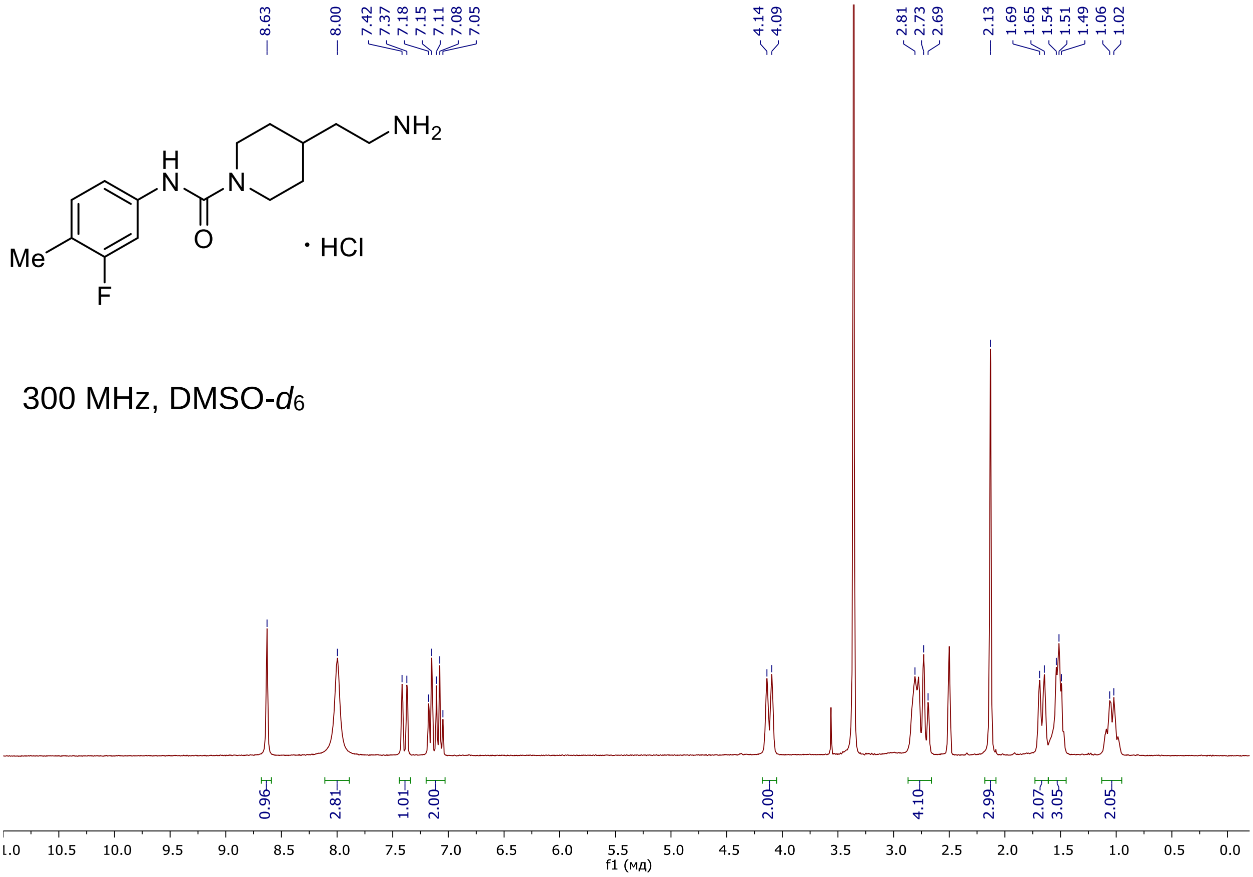

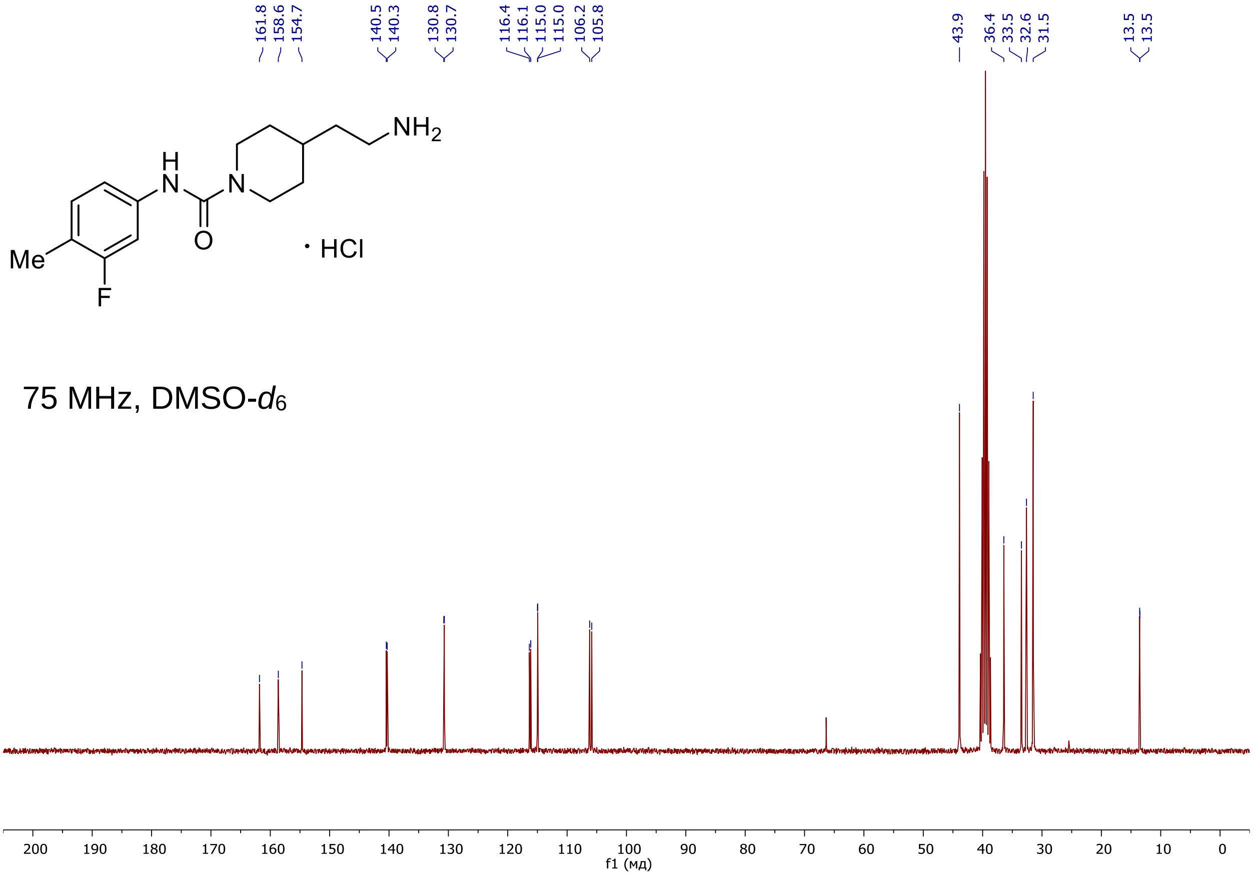

^{1}H and ^{13}C NMR spectra of compound **16**

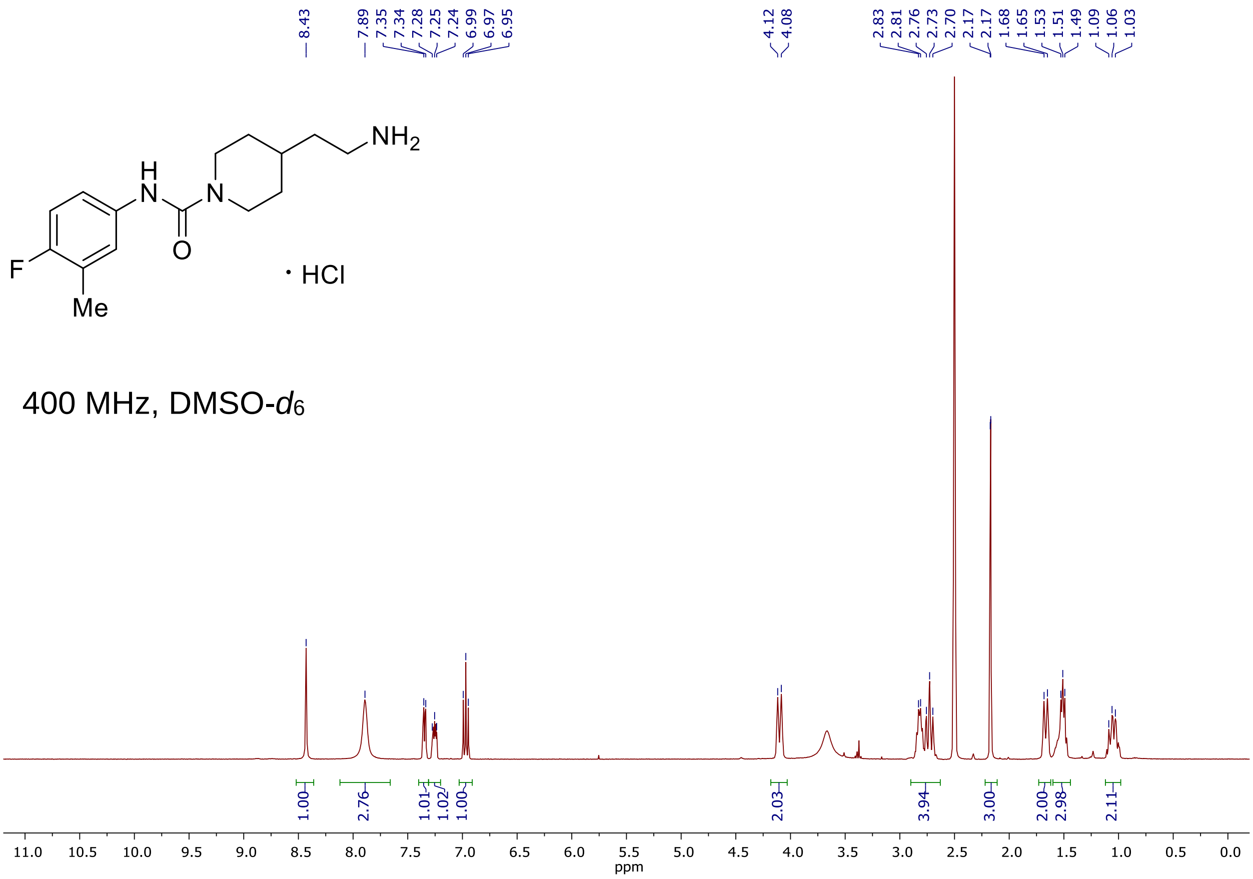

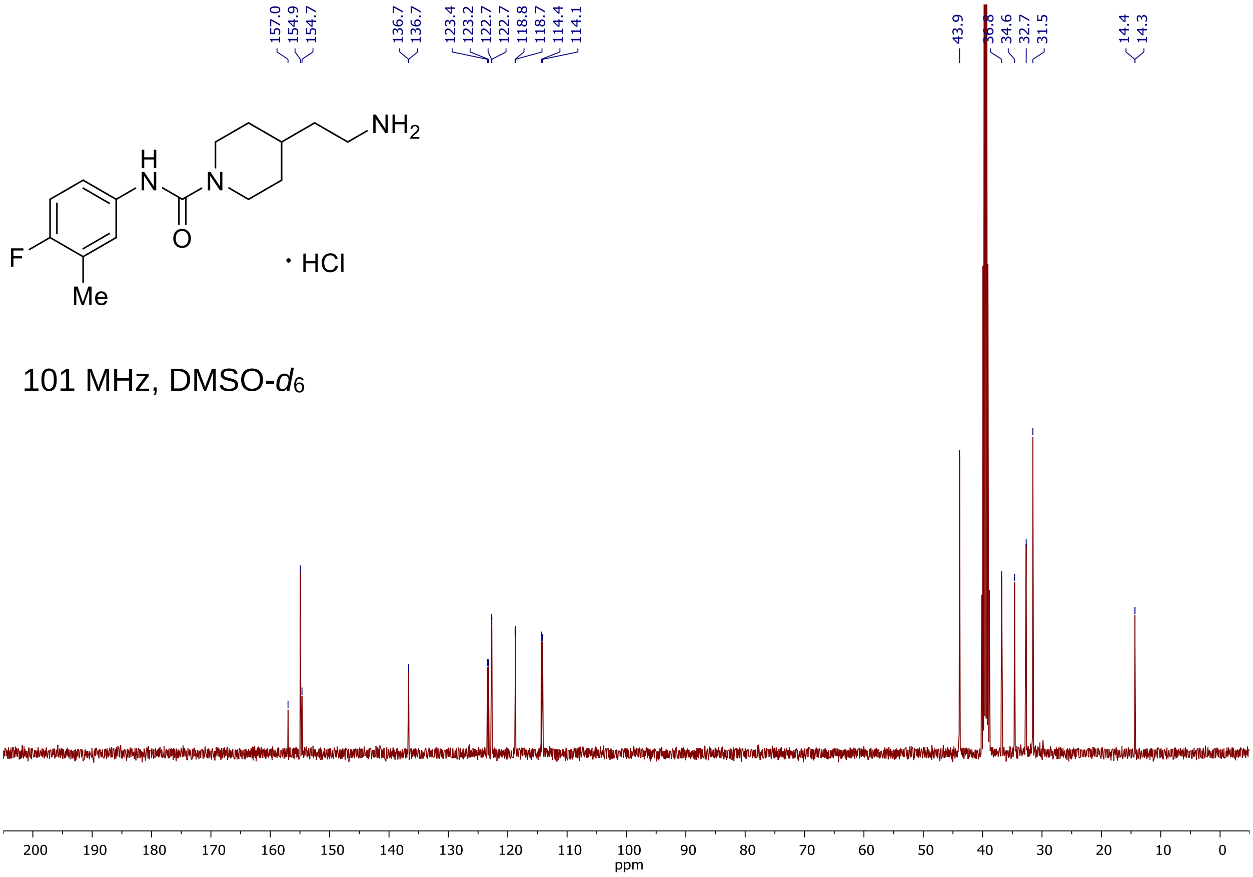

^{1}H and ^{13}C NMR spectra of compound **17**

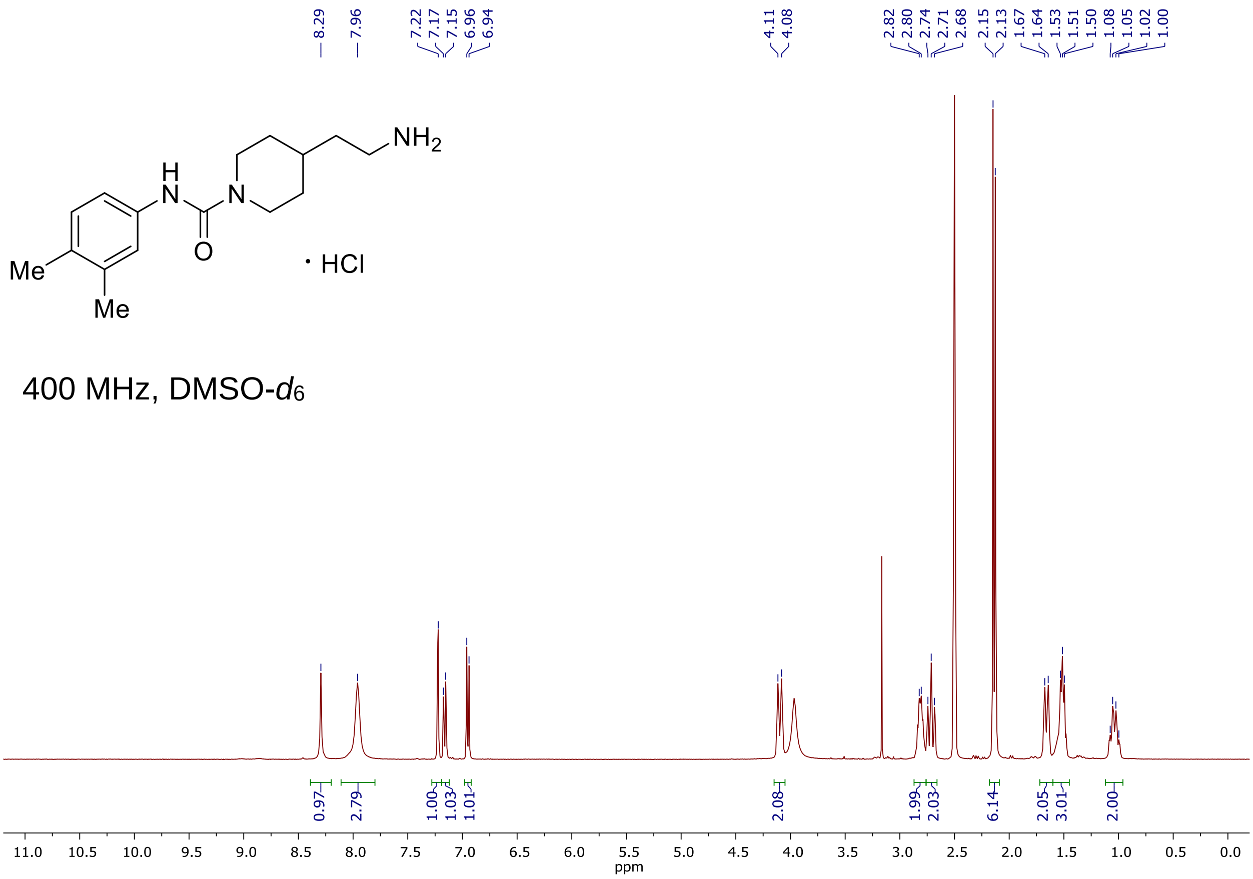

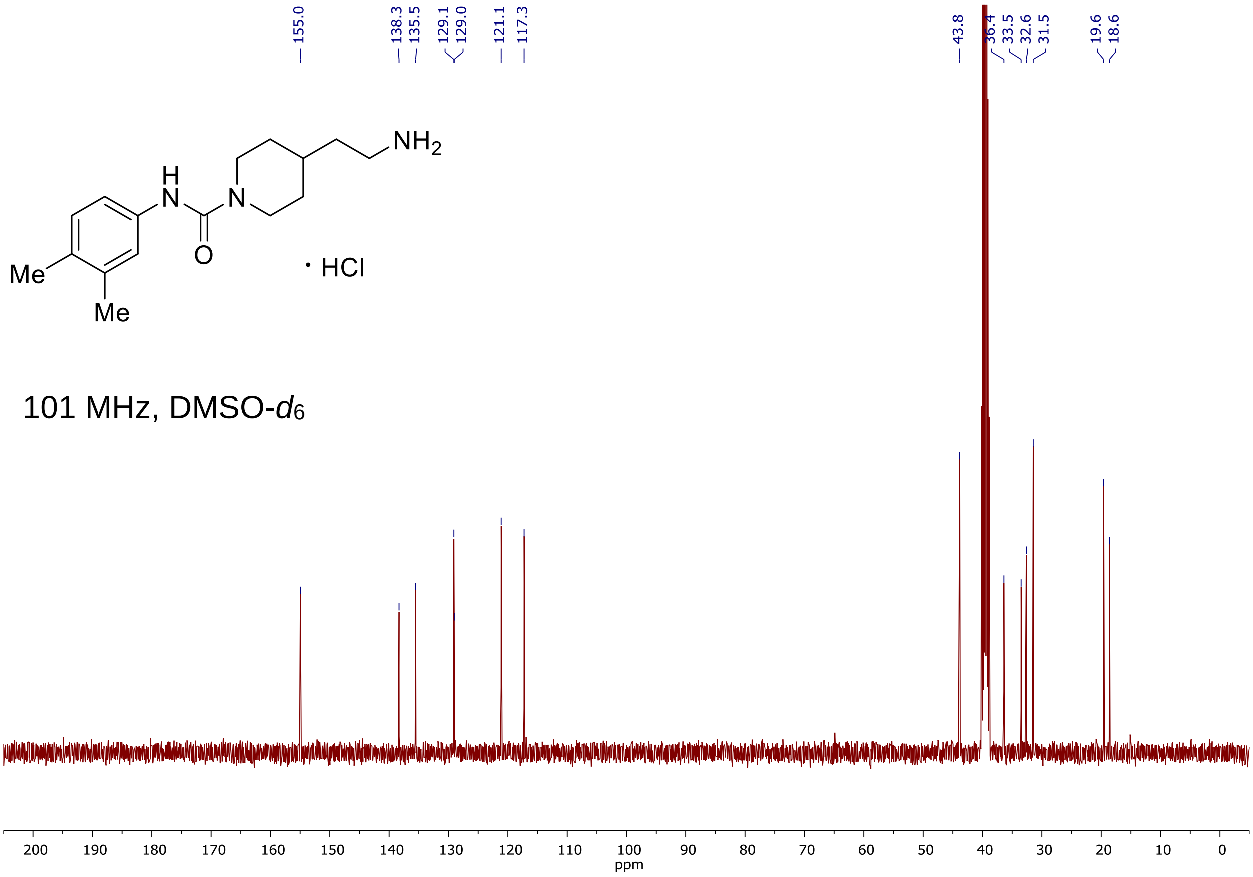

^{1}H and ^{13}C NMR spectra of compound **18** (AP163)

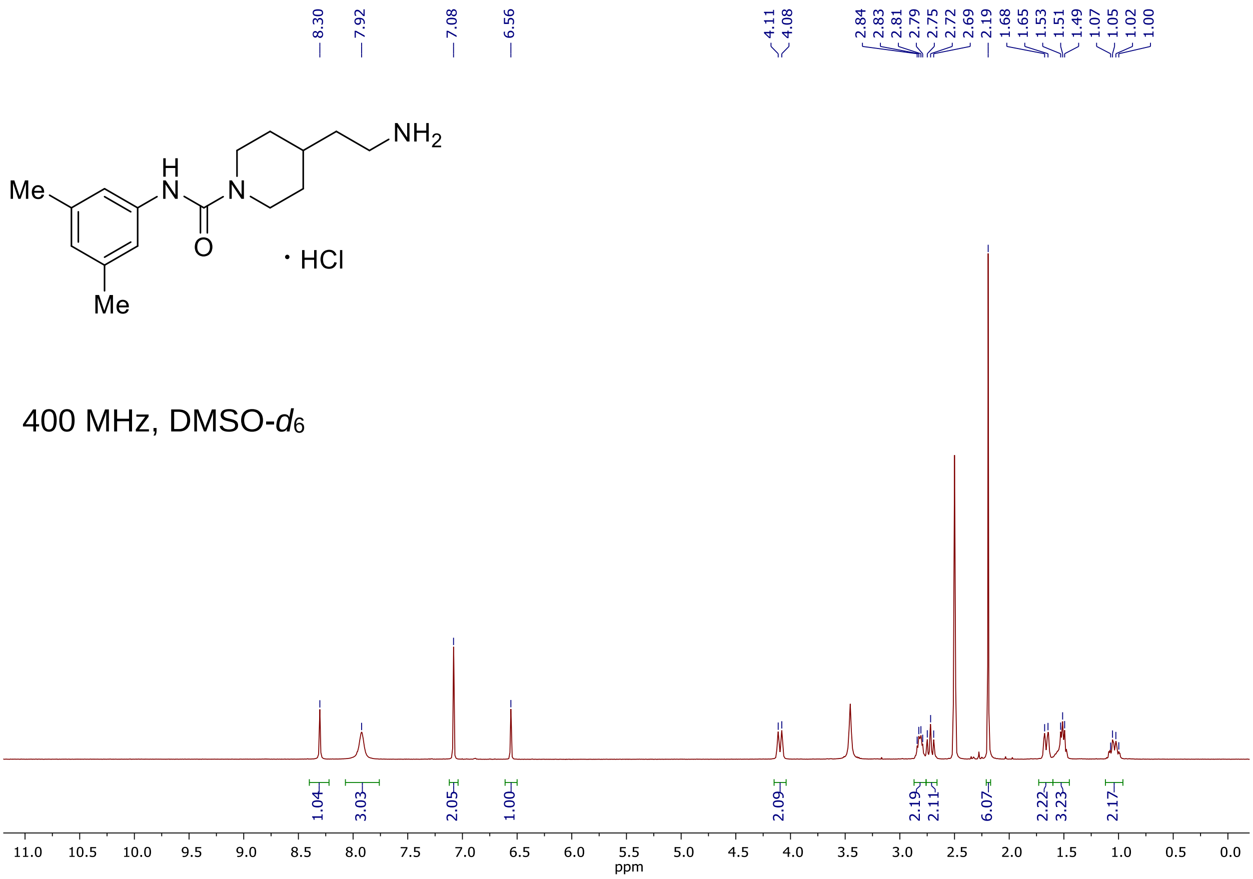

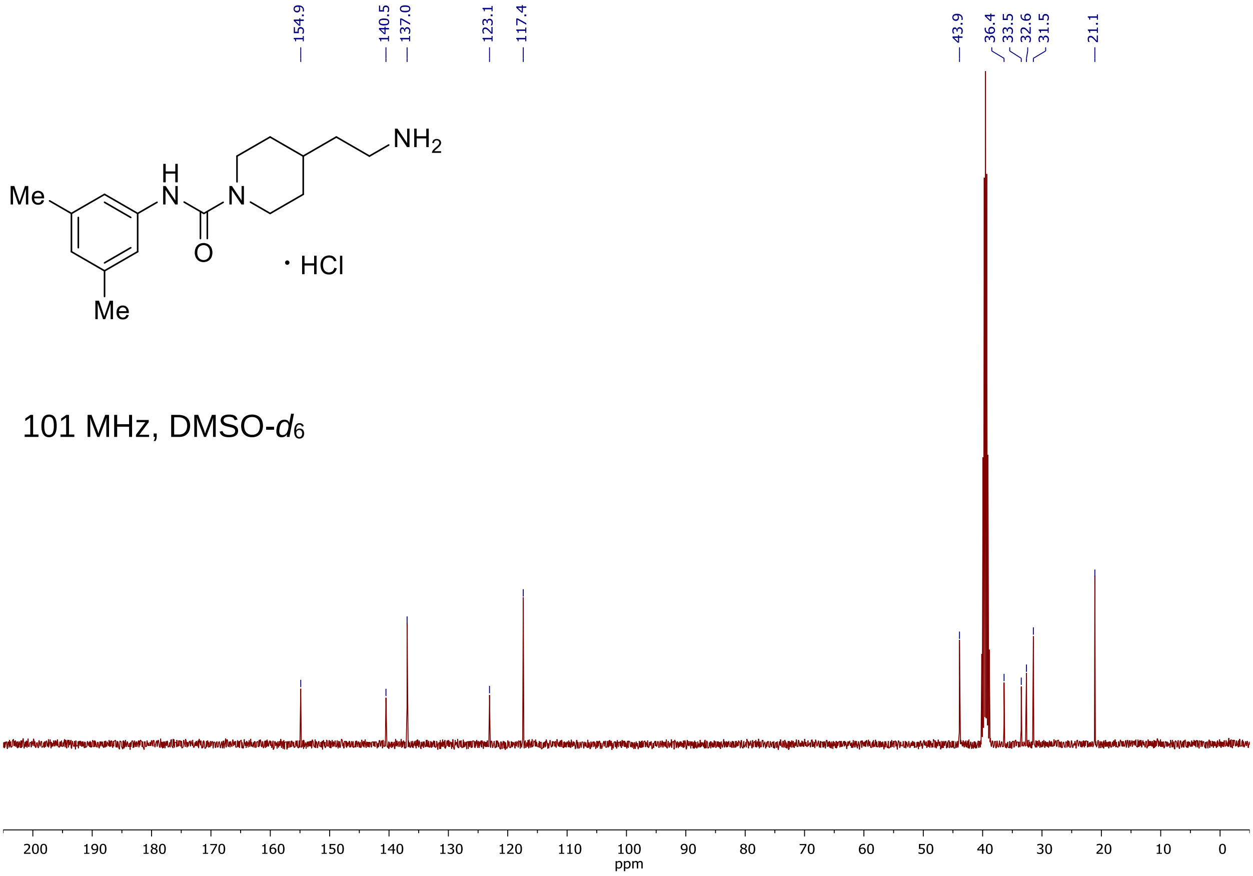

^{1}H and ^{13}C NMR spectra of compound **19**

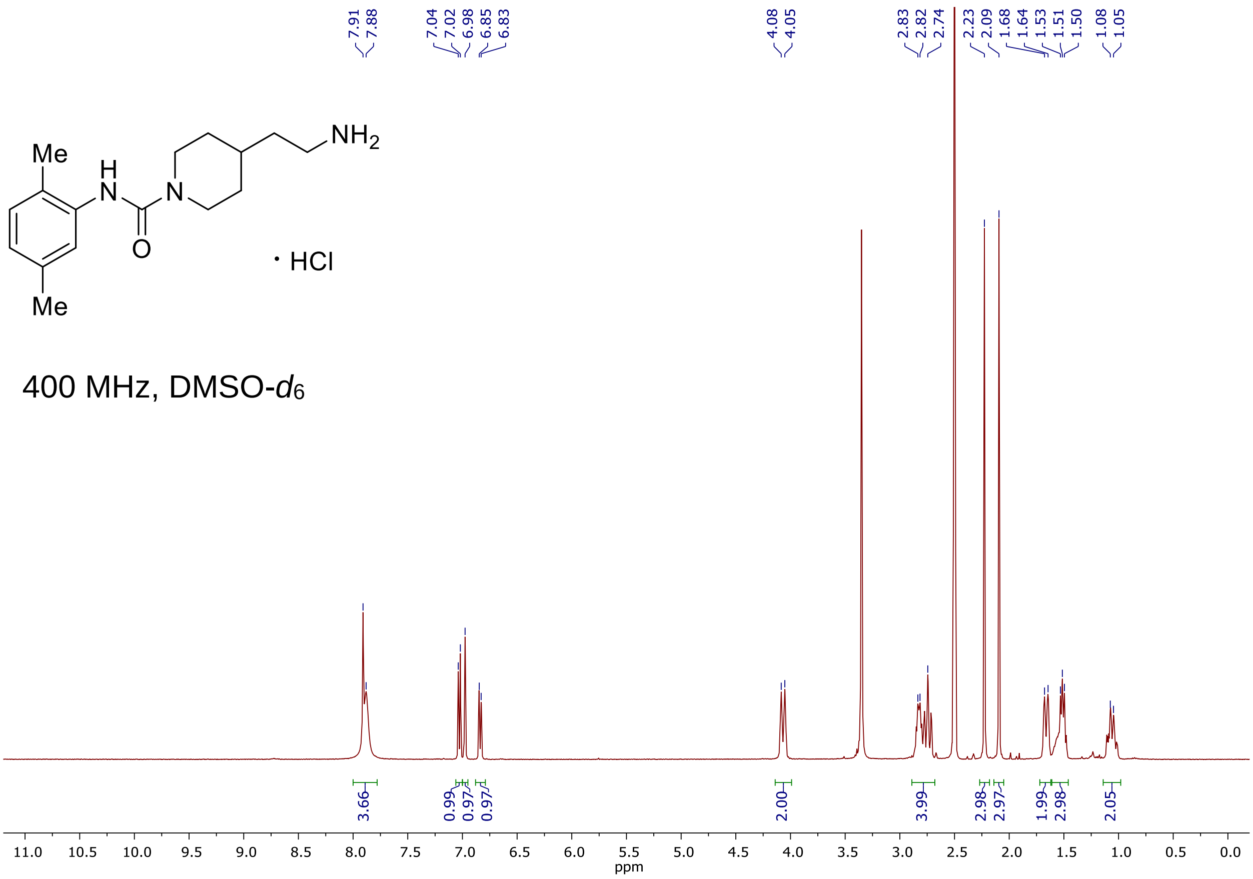

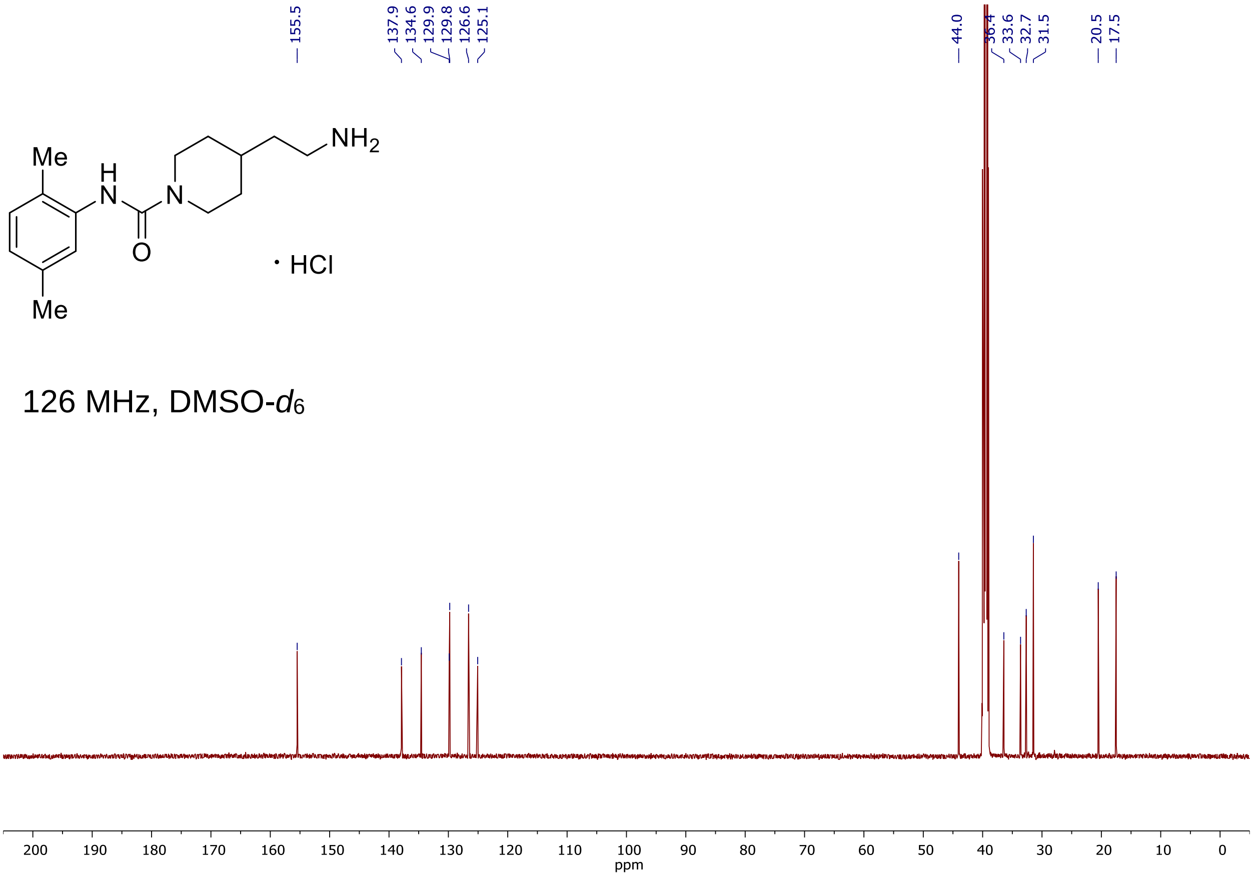

^{1}H and ^{13}C NMR spectra of compound **20**

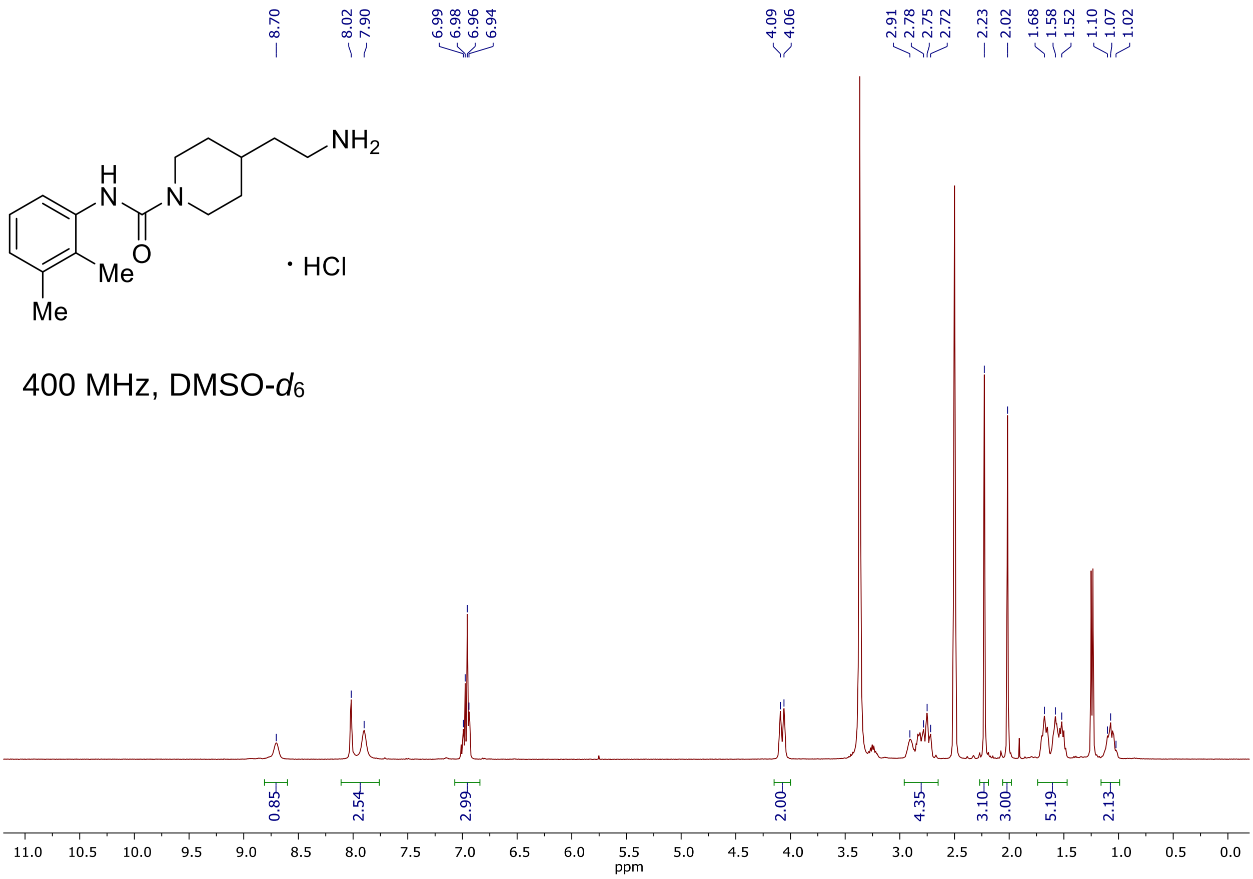

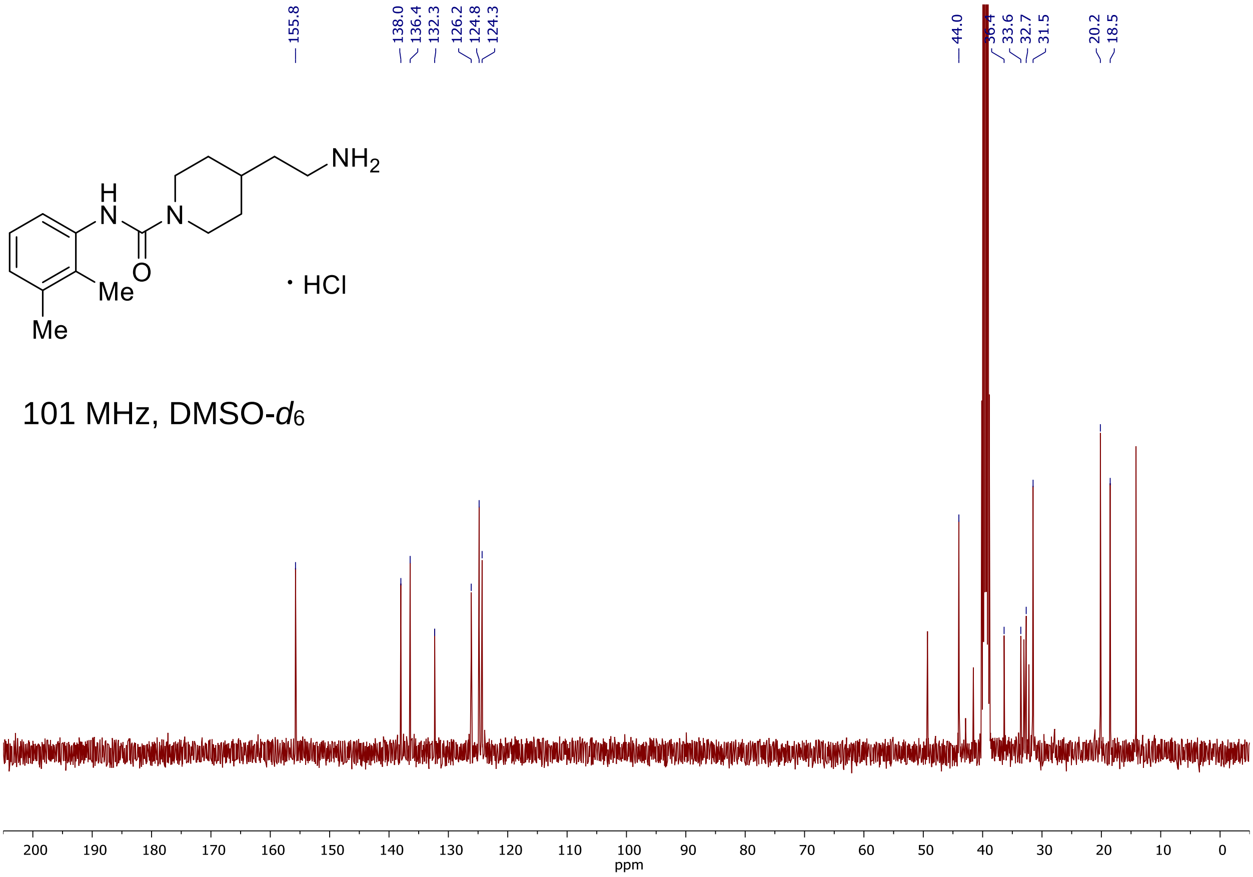

^{1}H and ^{13}C NMR spectra of compound **21**

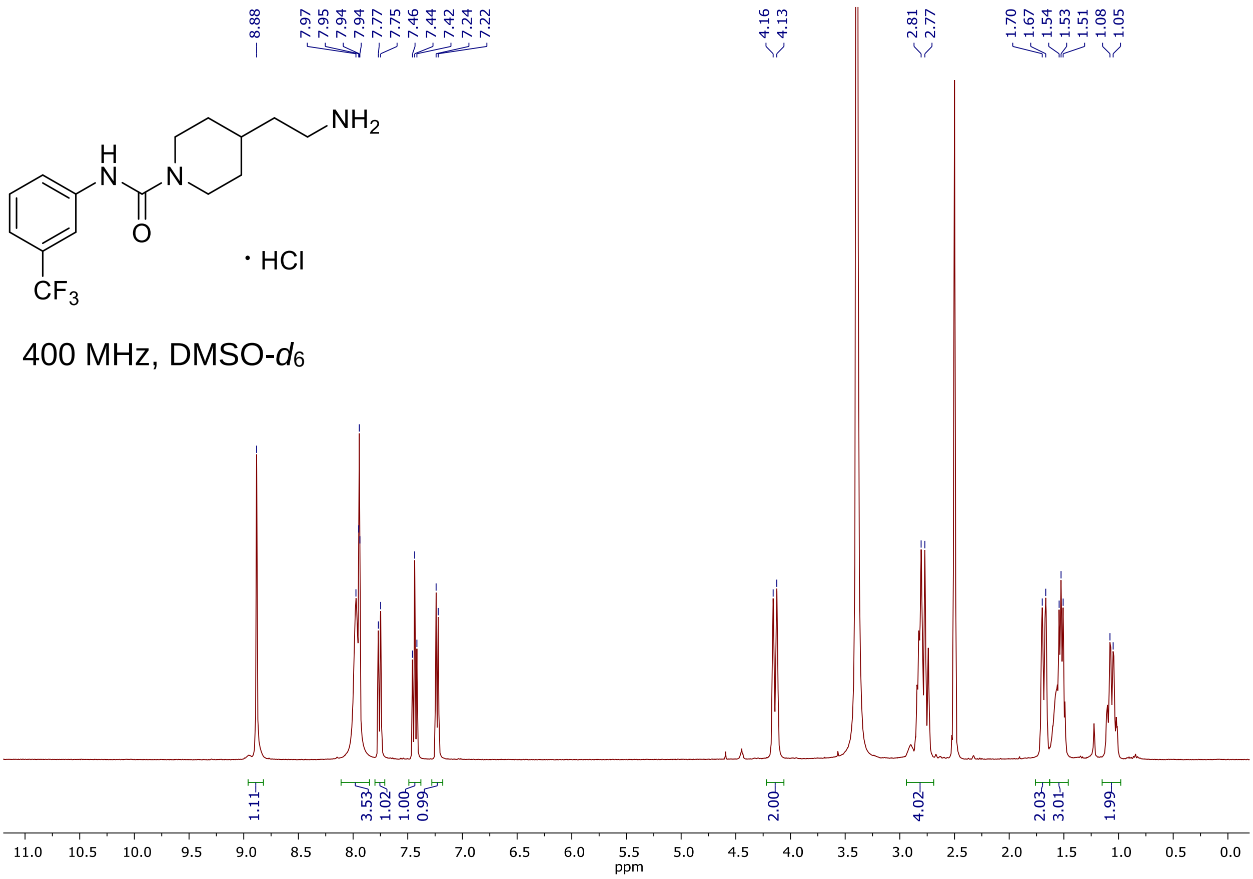

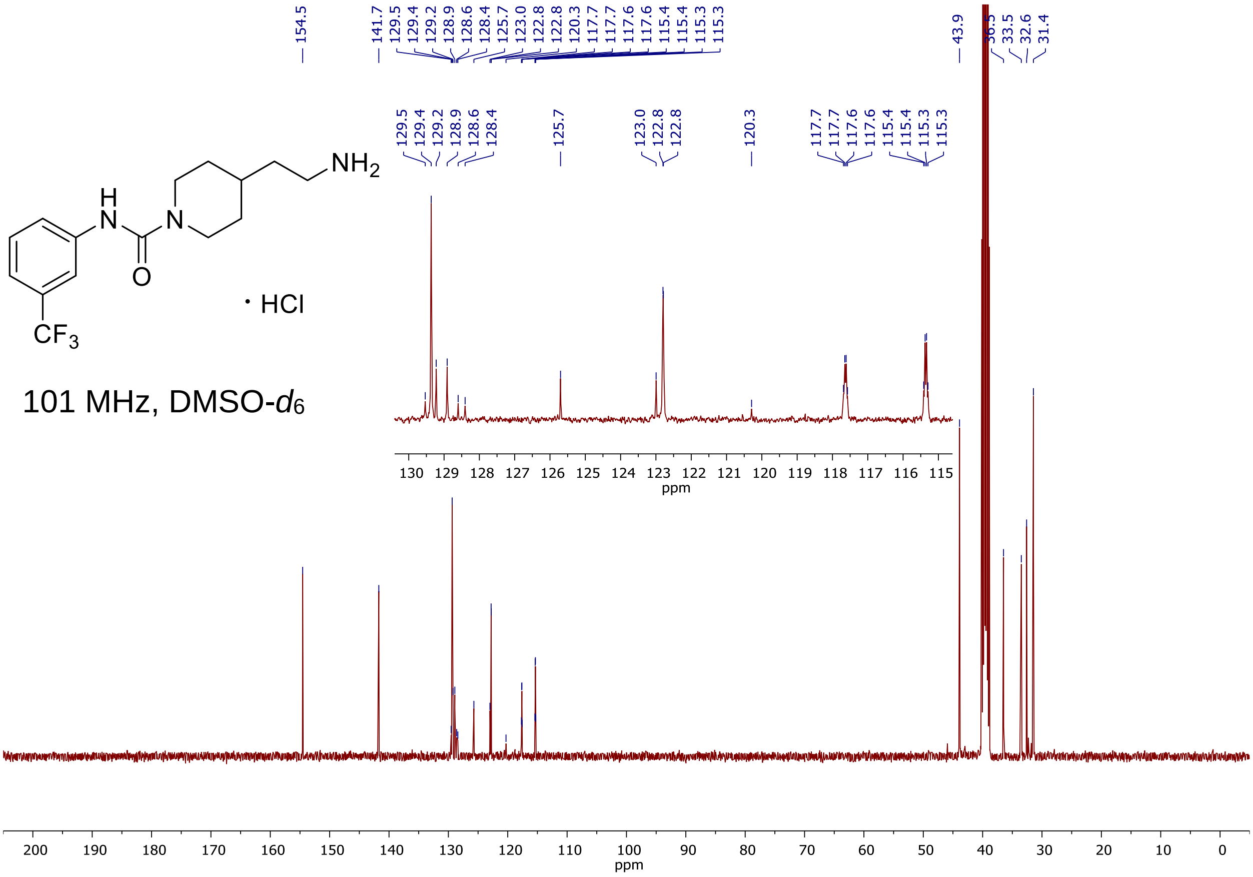

^{1}H and ^{13}C NMR spectra of compound **22**

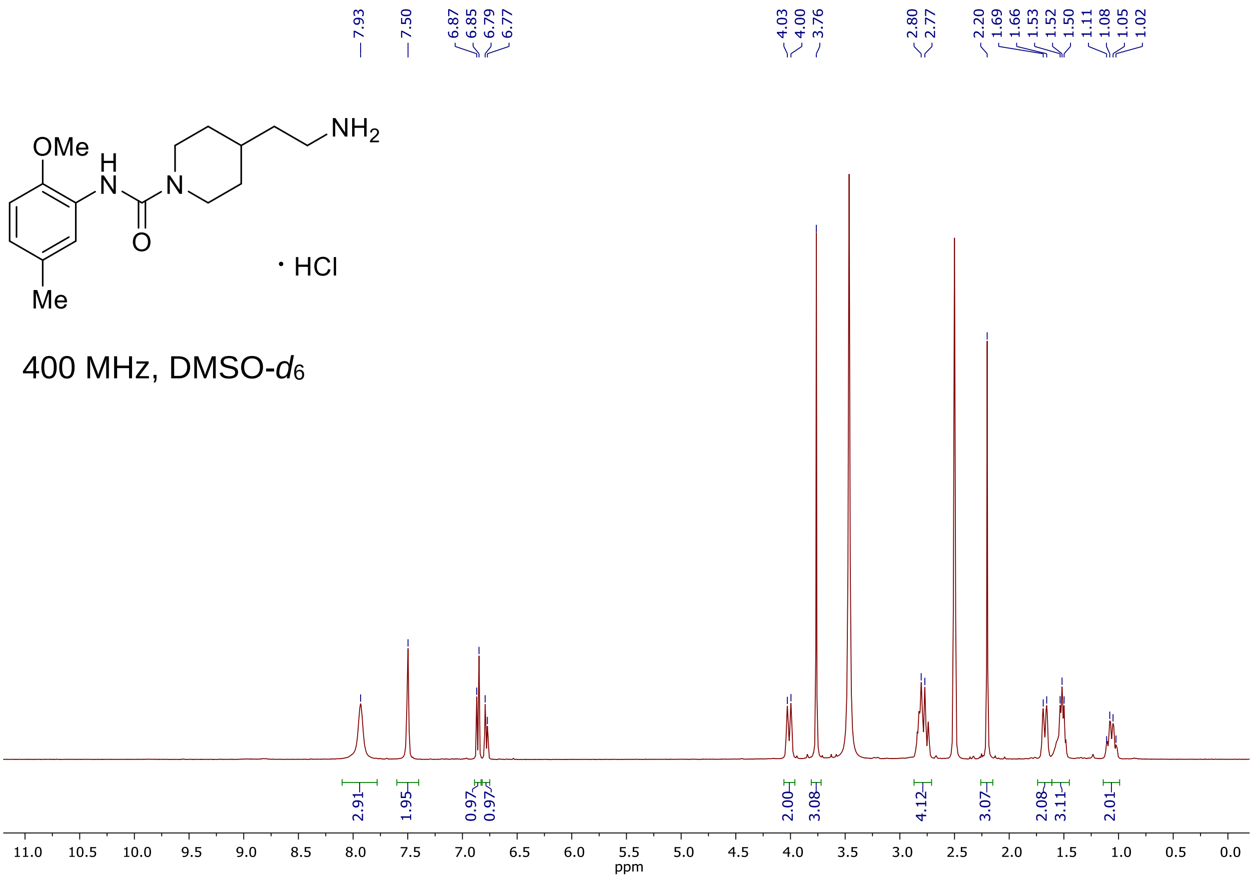

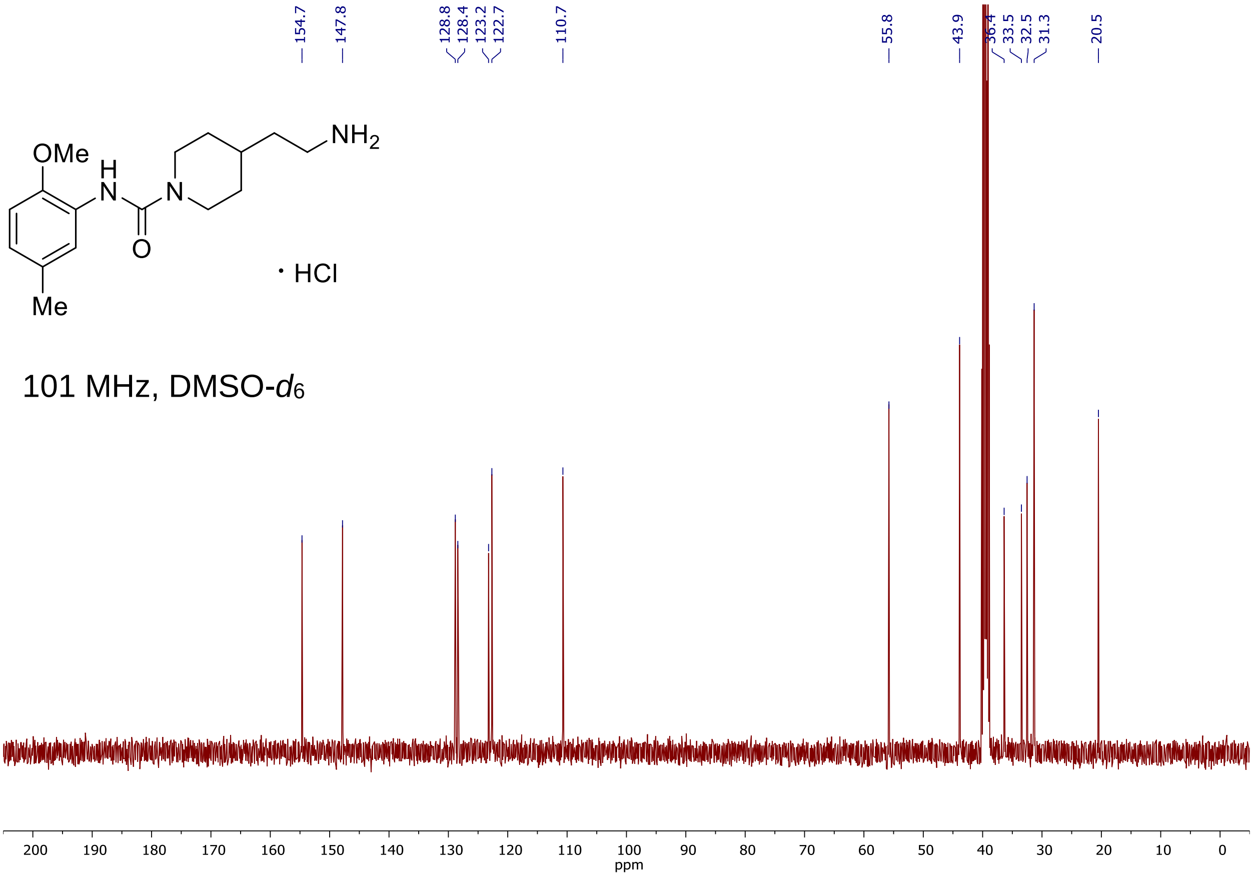

^{1}H and ^{13}C NMR spectra of compound **23**

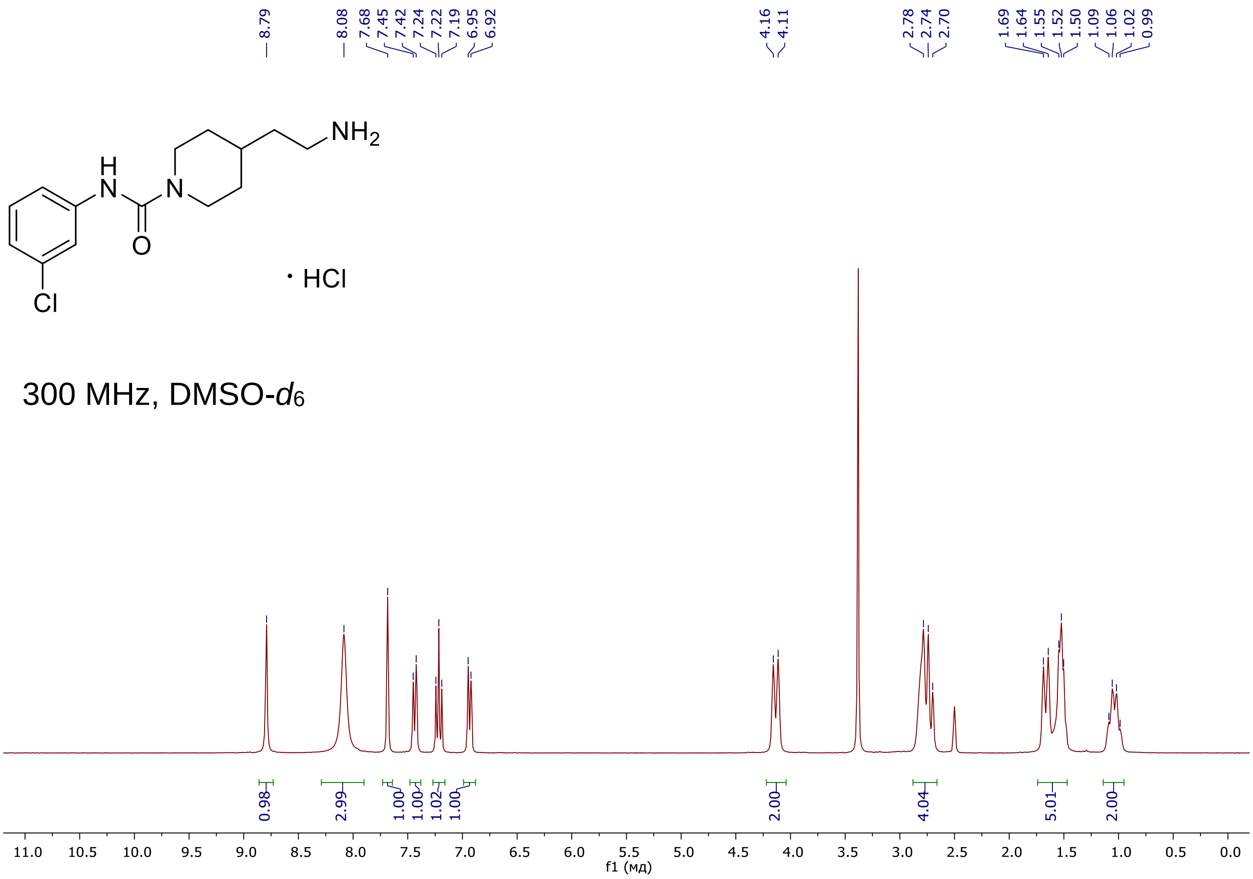

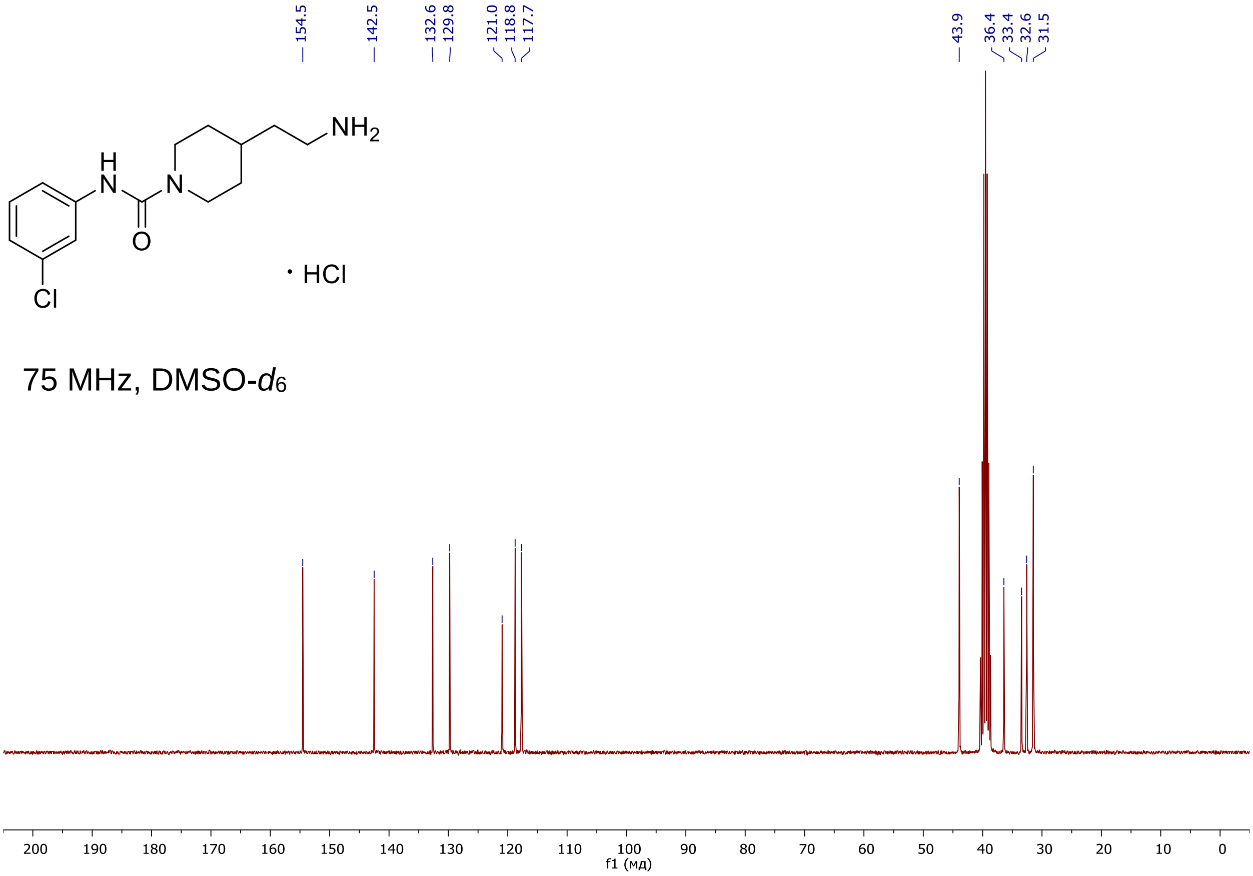

^{1}H and ^{13}C NMR spectra of compound **24**

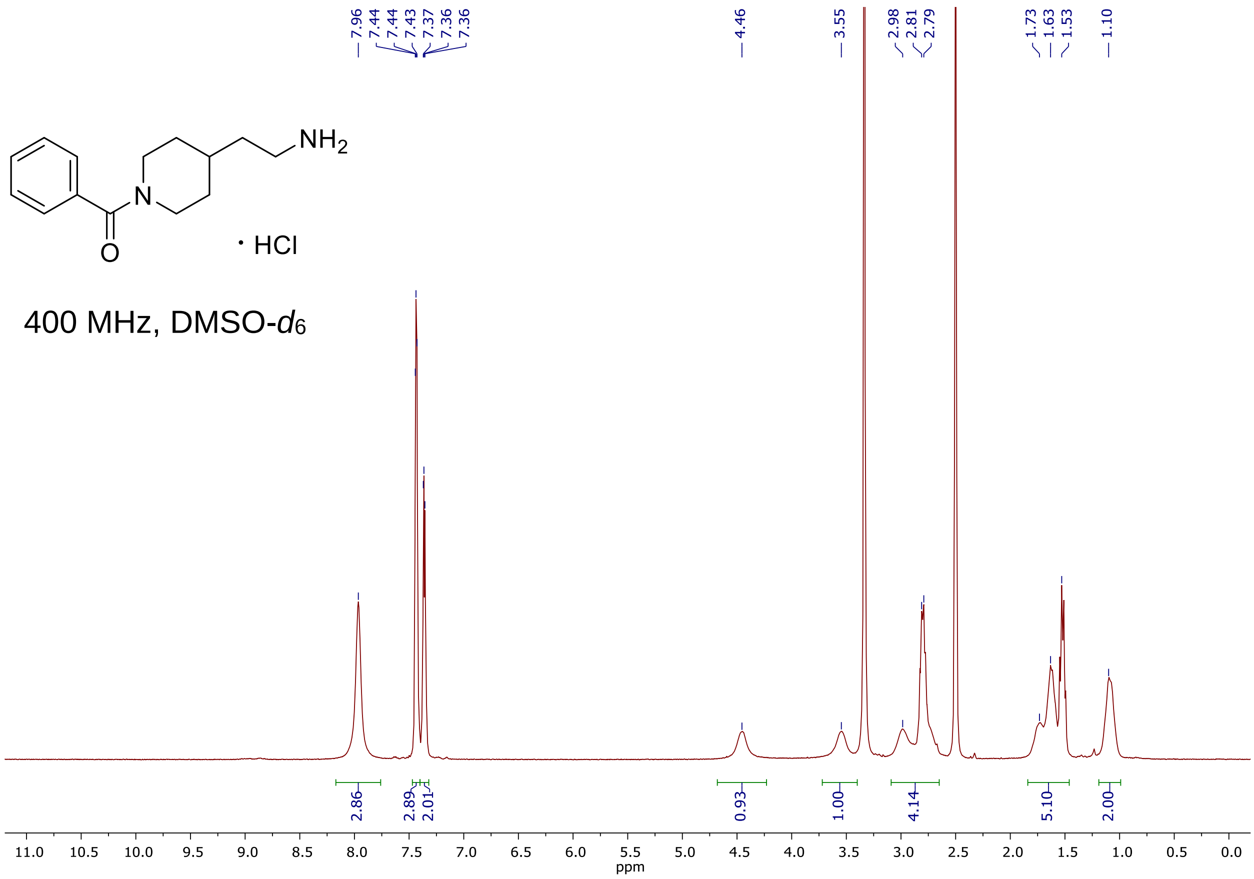

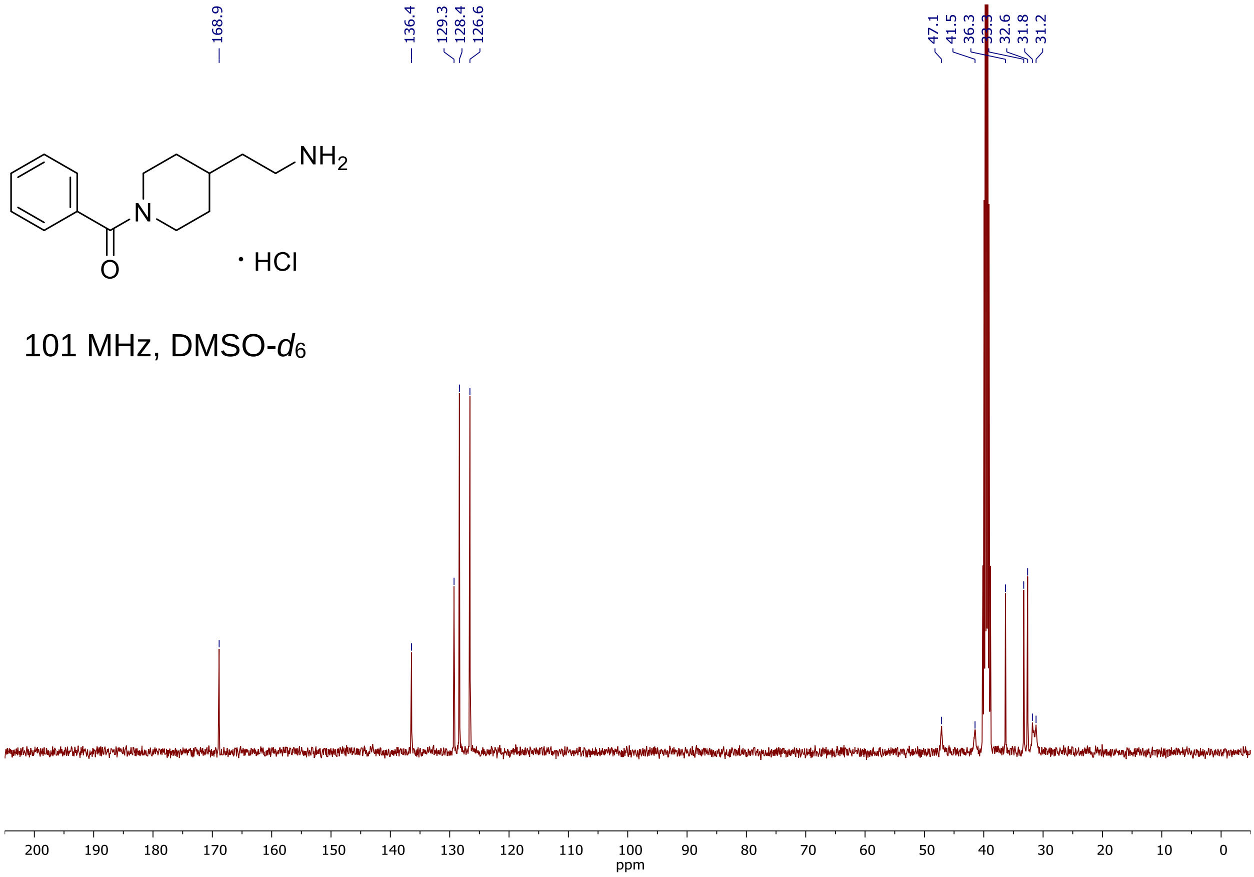

^{1}H and ^{13}C NMR spectra of compound **25**

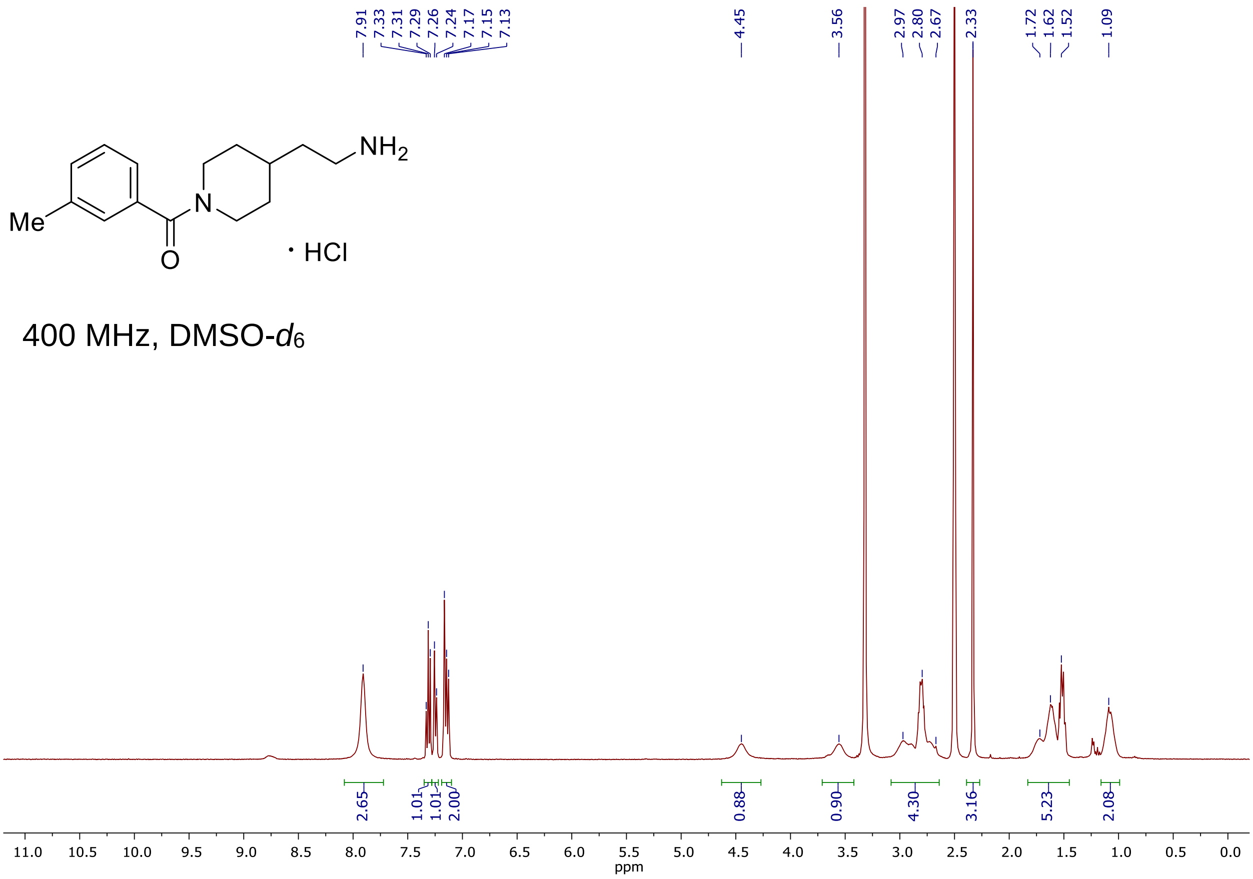

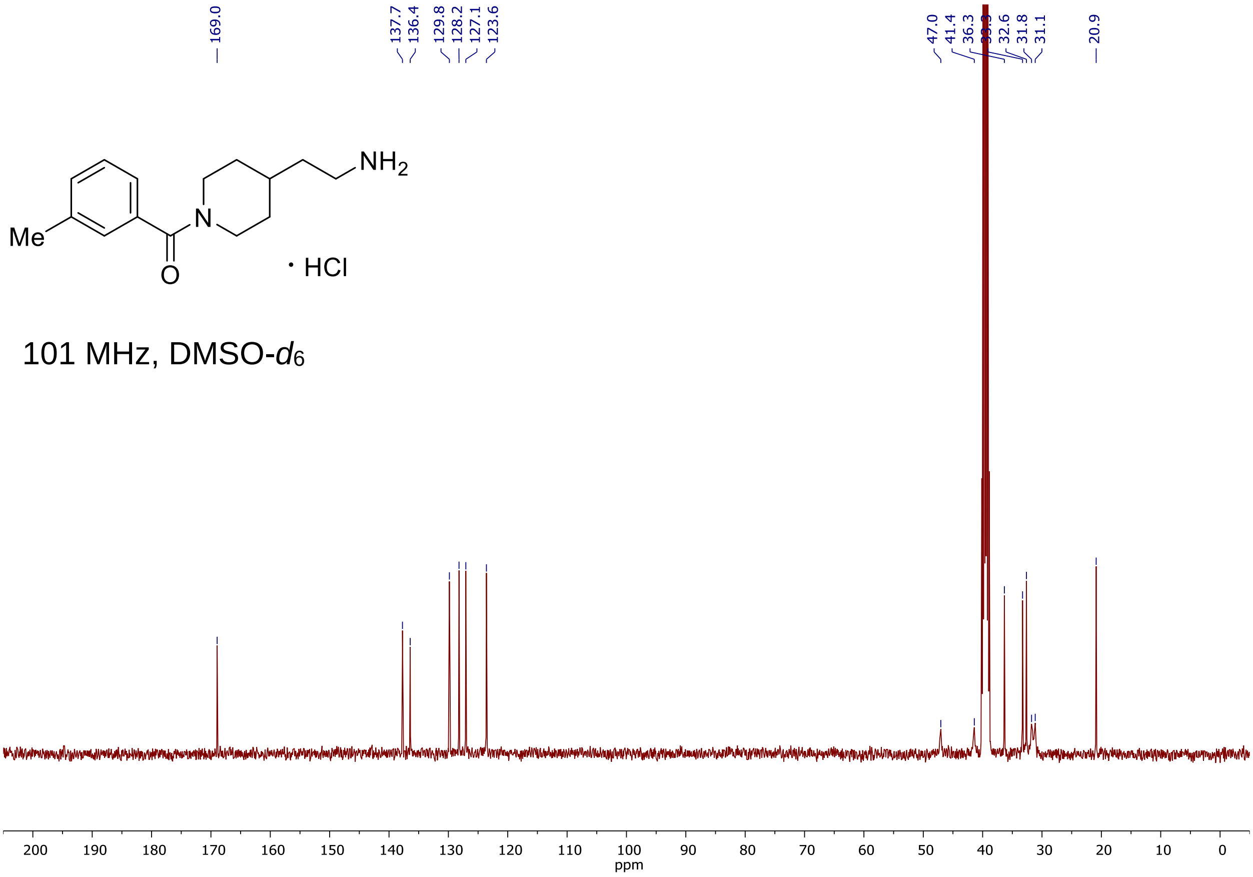

^{1}H and ^{13}C NMR spectra of compound **26**

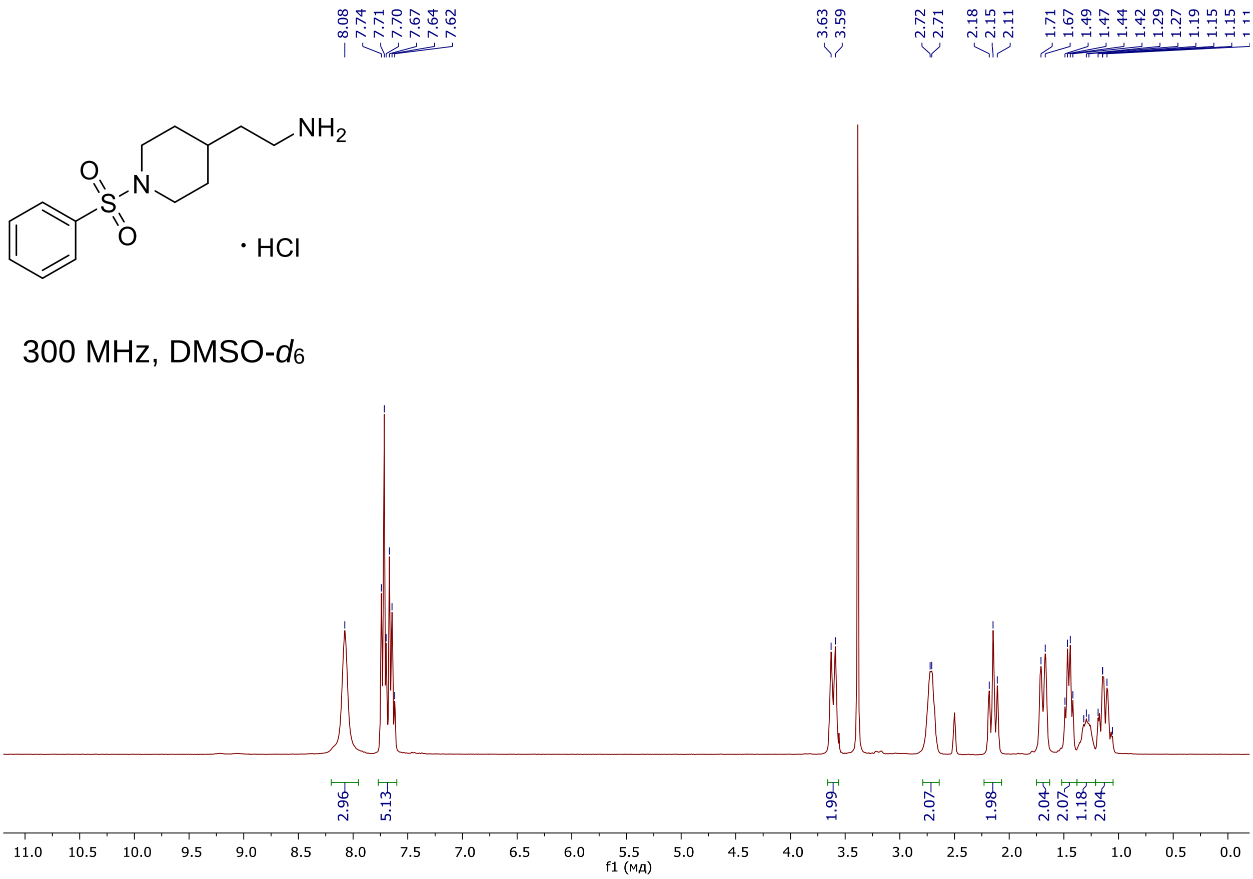

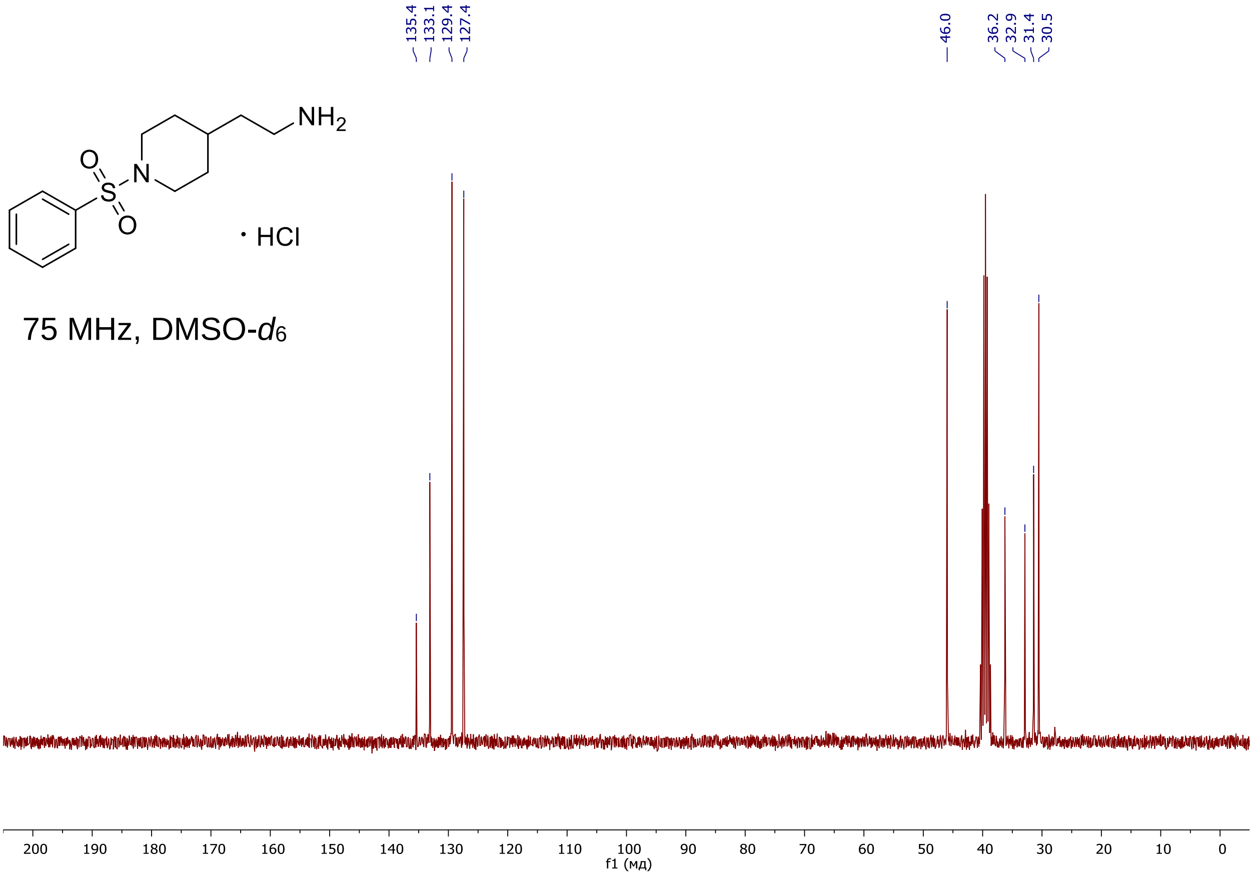

^{1}H and ^{13}C NMR spectra of compound **27**

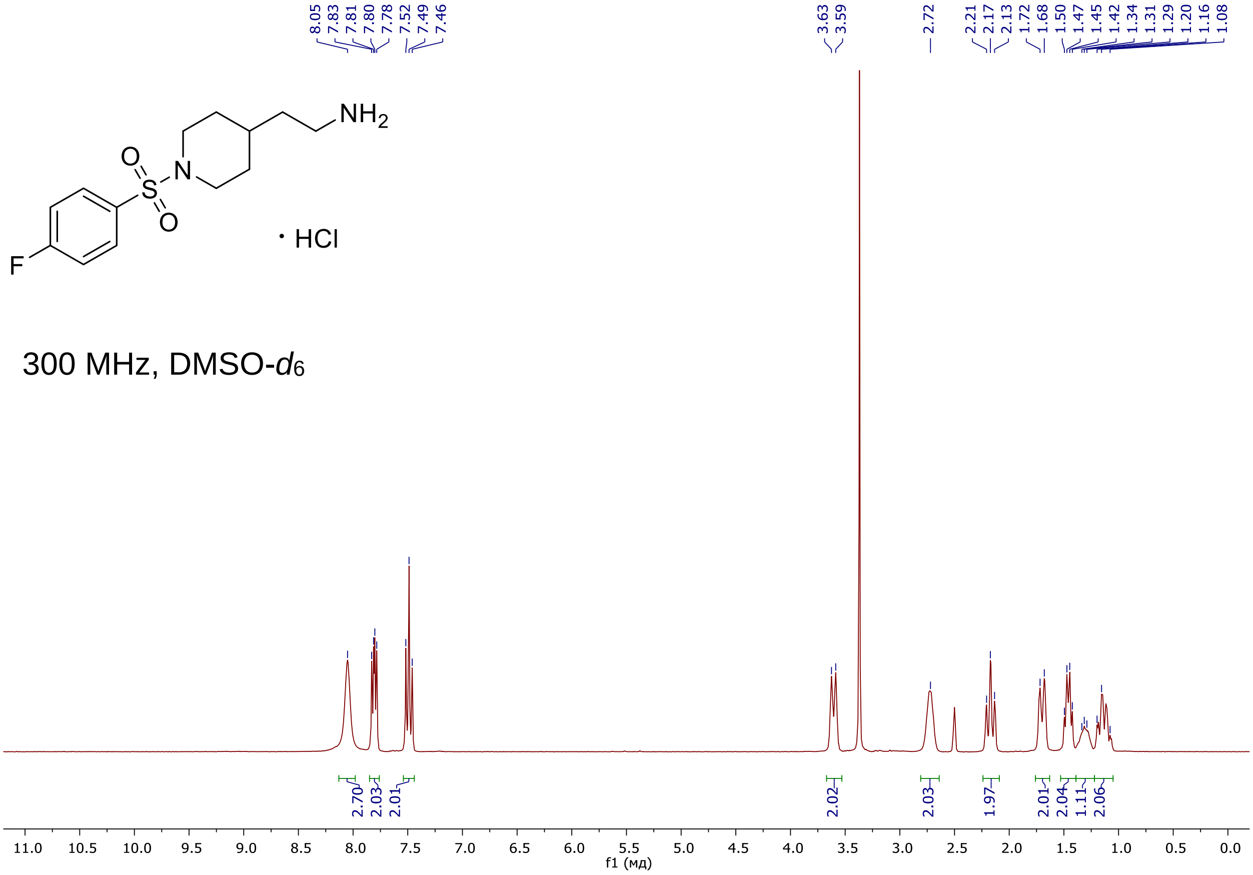

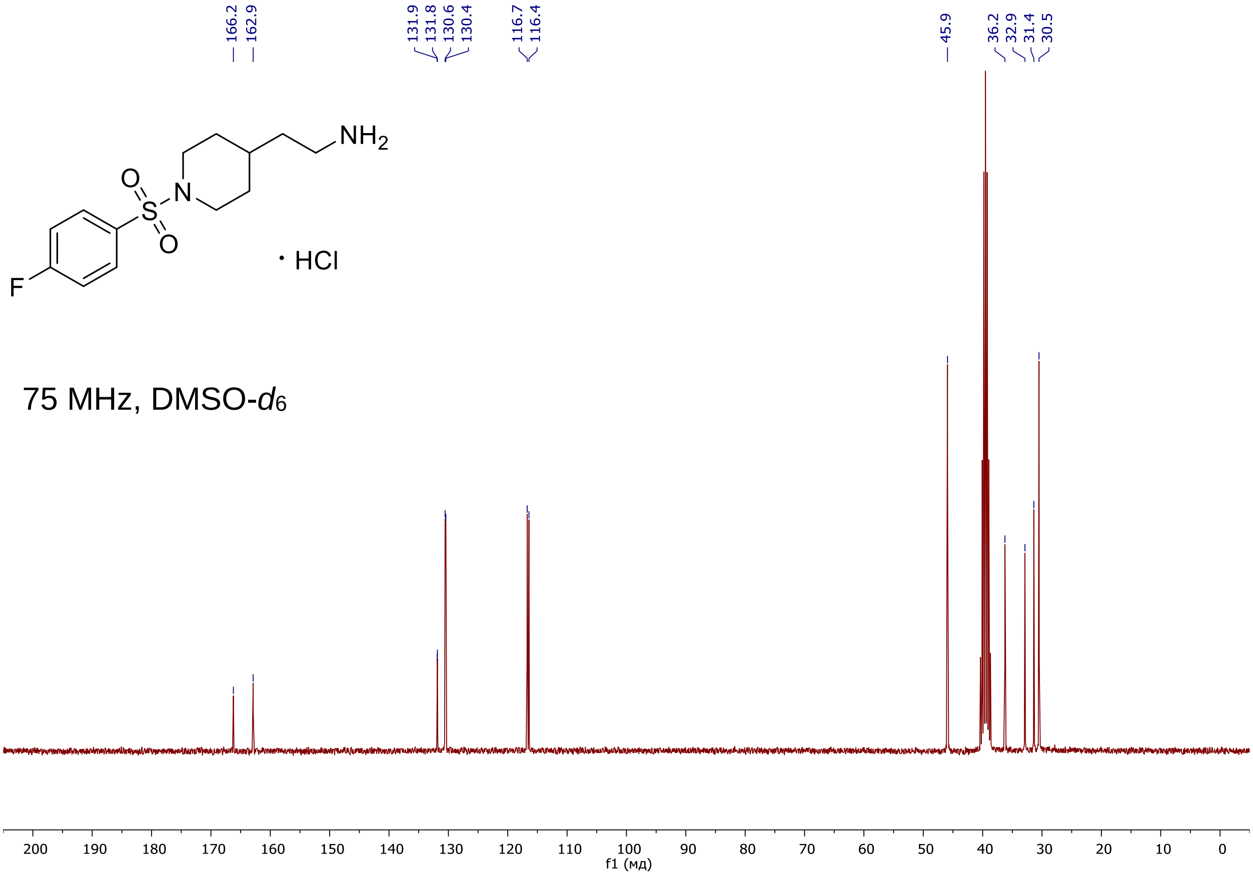

^{1}H and ^{13}C NMR spectra of compound **28**

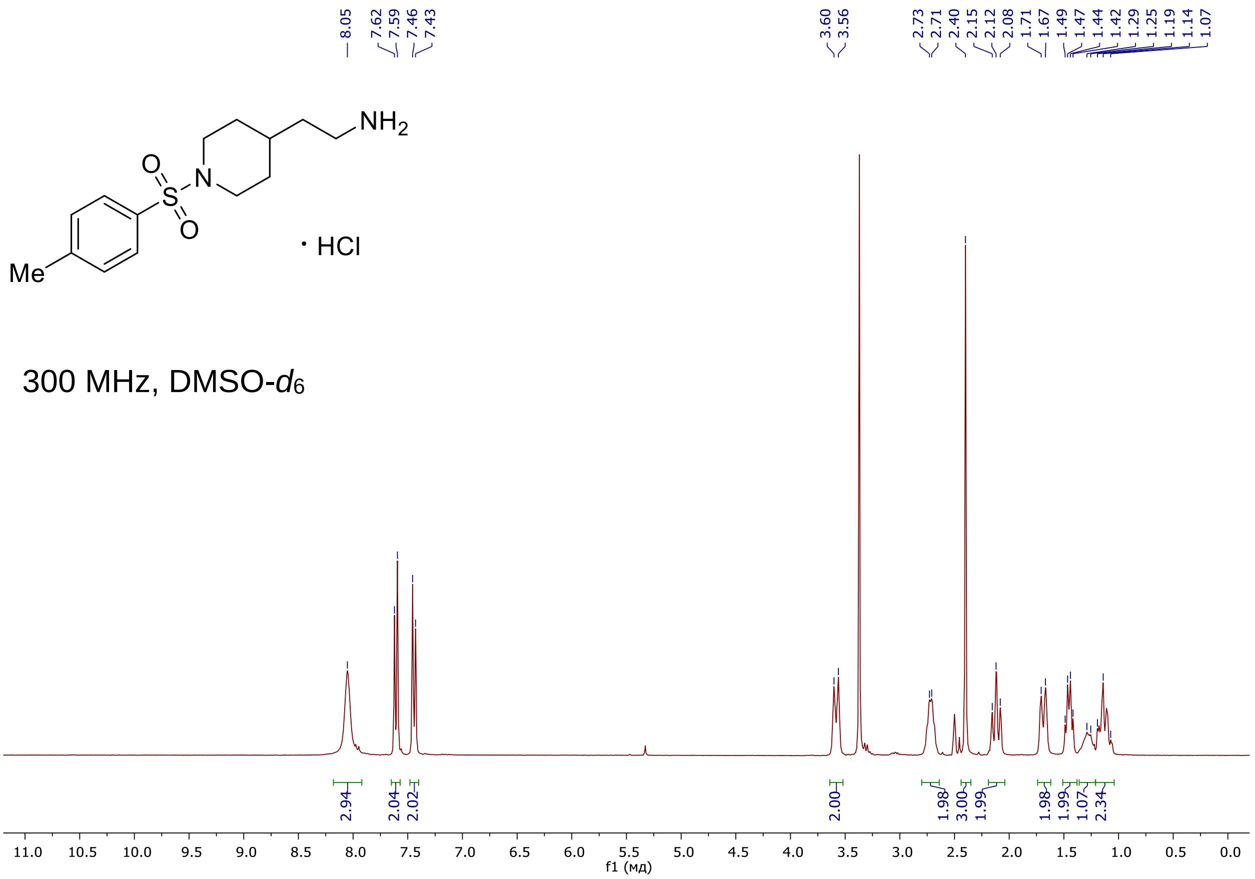

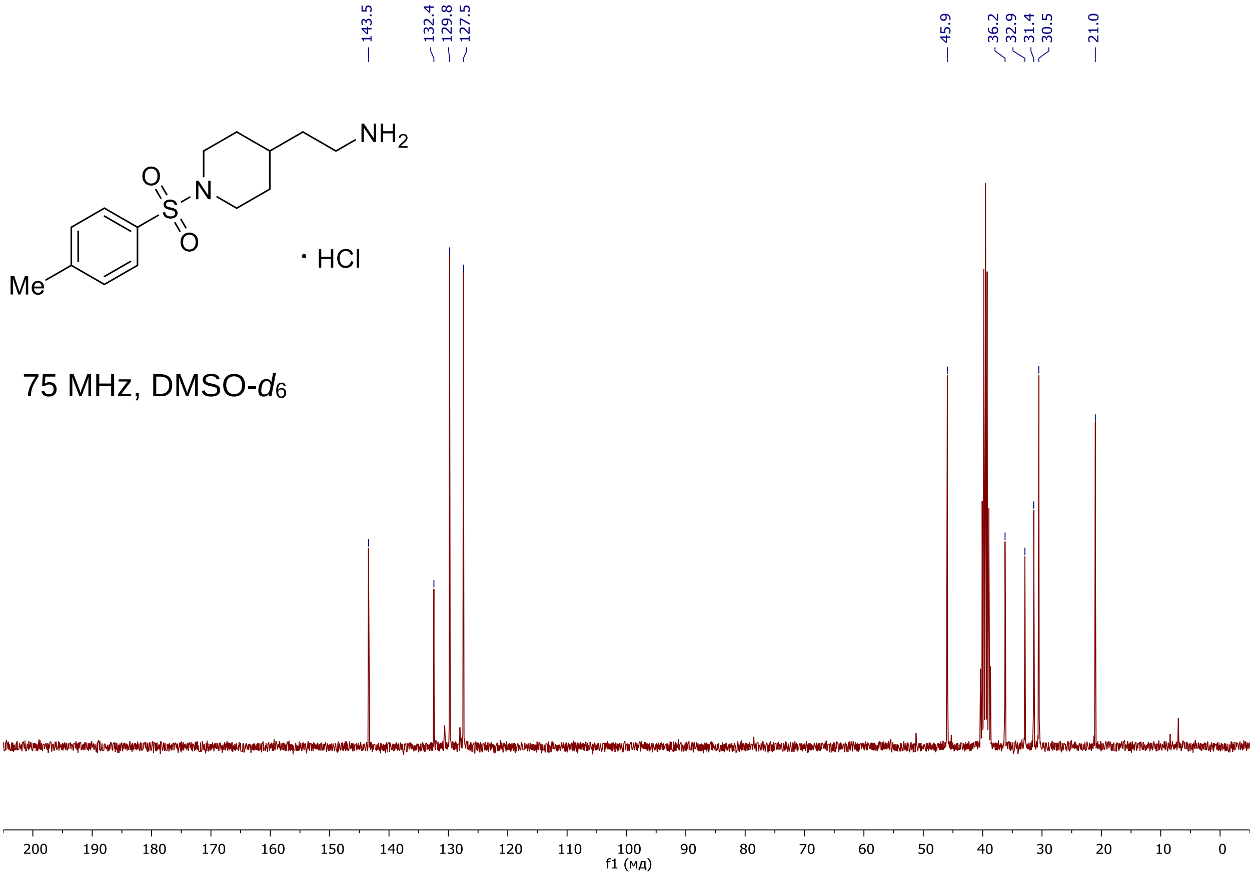

^1H and ^{13}C NMR spectra of compound **29**

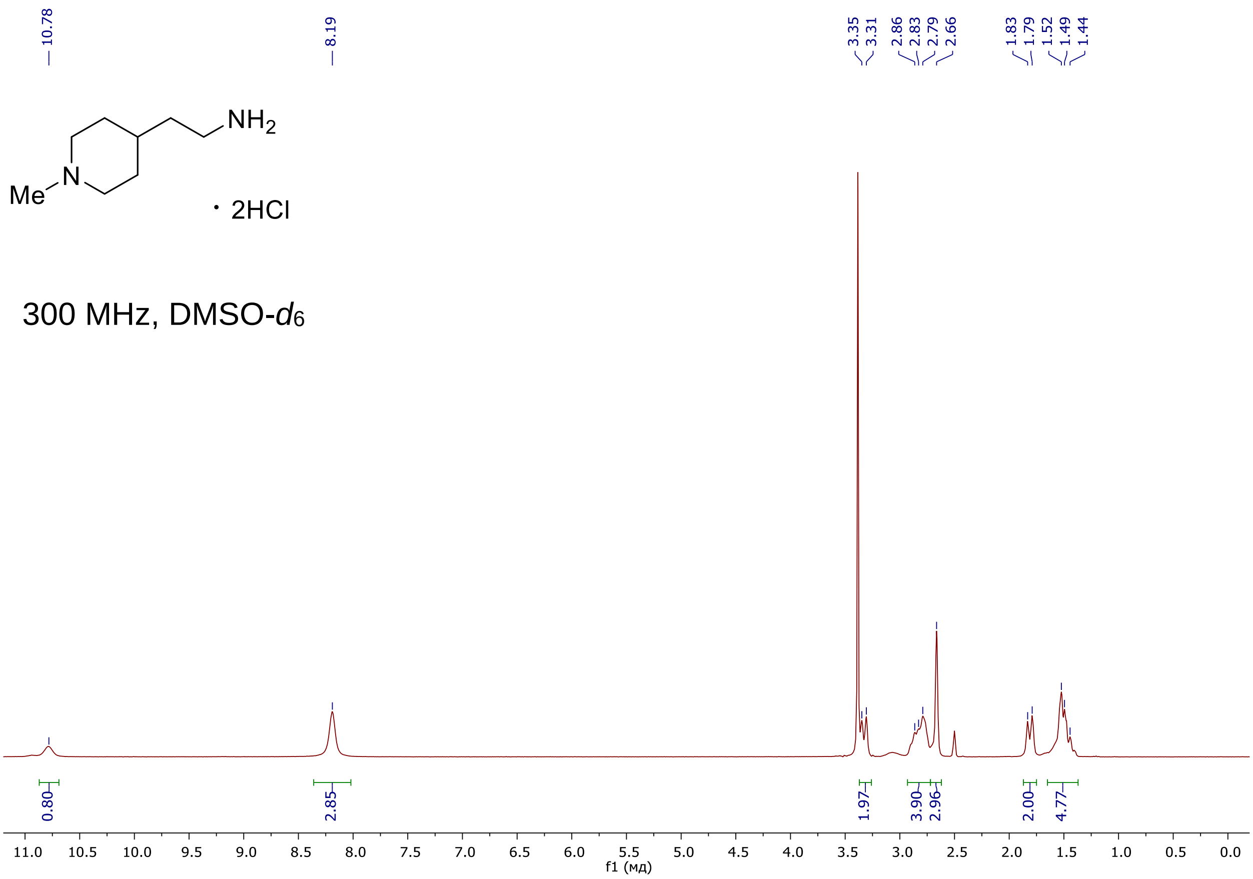

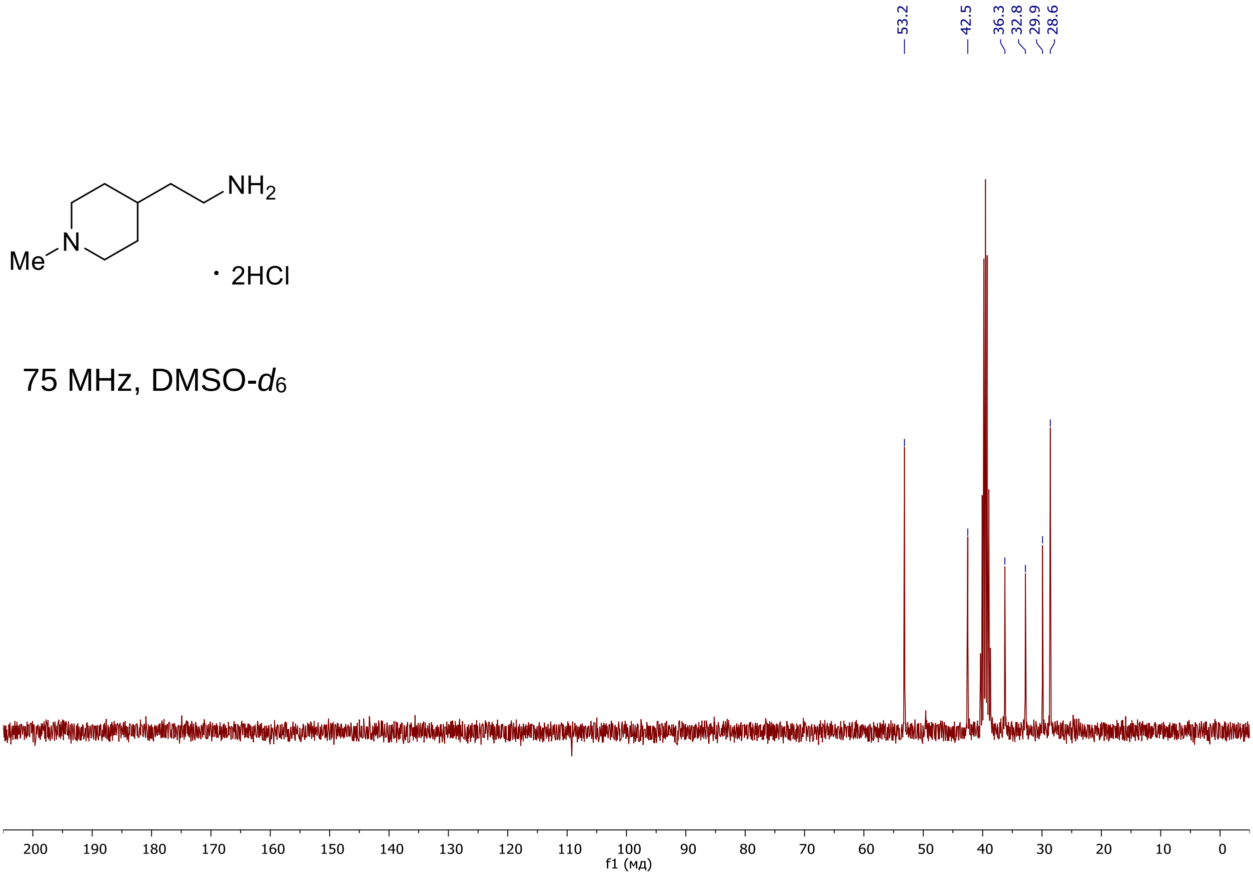

Calculated ADMET report from www.swissadme.ch for compound **18**

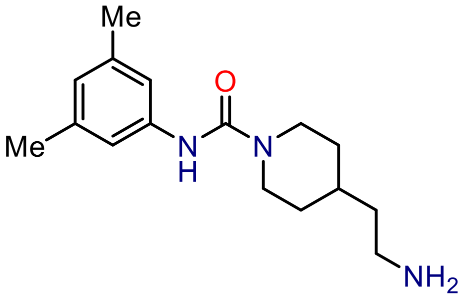

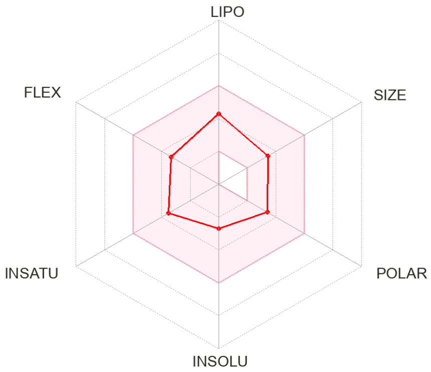

SMILES	NCCC1CCN(CC1)C(=O)Nc1cc(C)cc(c1)C

Physicochemical Properties

Formula	$C_{16}H_{25}N_3O$
Molecular weight	275.39 g/mol
Num. heavy atoms	20
Num. arom. heavy atoms	6
Fraction Csp3	0.56
Num. rotatable bonds	5
Num. H-bond acceptors	2
Num. H-bond donors	2
Molar Refractivity	87.32
TPSA	58.36 Å²

Lipophilicity

Log $P_{o/w}$ (iLOGP)	2.92
Log $P_{o/w}$ (XLOGP3)	2.00
Log $P_{o/w}$ (WLOGP)	2.32
Log $P_{o/w}$ (MLOGP)	2.45
Log $P_{o/w}$ (SILICOS-IT)	2.19
Consensus Log $P_{o/w}$	2.38

Water Solubility

Log *S* (ESOL)	-2.70
Solubility	5.50e-01 mg/ml ; 2.00e-03 mol/l
Class	Soluble
Log *S* (Ali)	-2.85
Solubility	3.87e-01 mg/ml ; 1.41e-03 mol/l
Class	Soluble
Log *S* (SILICOS-IT)	-4.12
Solubility	2.11e-02 mg/ml ; 7.66e-05 mol/l
Class	Moderately soluble

Pharmacokinetics

GI absorption	High
BBB permeant	Yes
P-gp substrate	Yes
CYP1A2 inhibitor	No
CYP2C19 inhibitor	No
CYP2C9 inhibitor	No
CYP2D6 inhibitor	Yes
CYP3A4 inhibitor	No
Log K_p (skin permeation)	-6.56 cm/s

Druglikeness

Lipinski	Yes; 0 violation
Ghose	Yes
Veber	Yes
Egan	Yes
Muegge	Yes
Bioavailability Score	0.55

Medicinal Chemistry

PAINS	0 alert
Brenk	0 alert
Leadlikeness	Yes
Synthetic accessibility	2.17
